# Ranitidine Use and Incident Cancer in a Multinational Cohort

**DOI:** 10.1001/jamanetworkopen.2023.33495

**Published:** 2023-09-19

**Authors:** Seng Chan You, Seung In Seo, Thomas Falconer, Chen Yanover, Talita Duarte-Salles, Sarah Seager, Jose D. Posada, Nigam H. Shah, Phung-Anh Nguyen, Yeesuk Kim, Jason C. Hsu, Mui Van Zandt, Min-Huei Hsu, Hang Lak Lee, Heejoo Ko, Woon Geon Shin, Nicole Pratt, Rae Woong Park, Christin G. Reich, Marc A. Suchard, George Hripcsak, Chan Hyuk Park, Daniel Prieto-Alhambra

**Affiliations:** 1 Department of Biomedical Systems Informatics, Yonsei University College of Medicine, Seoul, Korea; 2 Institute for Innovation in Digital Healthcare, Yonsei University, Seoul, Korea; 3 Department of Internal Medicine, Kangdong Sacred Heart Hospital, Hallym University College of Medicine, Seoul, Korea; 4 Institute for Liver and Digestive Diseases, Hallym University, Chuncheon, Korea; 5 Department of Biomedical Informatics, Columbia University, New York, New York; 6 KI Research Institute, Kfar Malal, Israel; 7 Fundació Institut Universitari per a la recerca a l’Atenció Primària de Salut Jordi Gol i Gurina, Barcelona, Spain; 8 IQVIA, Cambridge, Massachusetts; 9 Department of Medicine, Stanford University School of Medicine, Stanford, California; 10 Graduate Institute of Data Science, College of Management, Taipei Medical University, Taiwan; 11 Department of Orthopaedic Surgery, College of Medicine, Hanyang University, Seoul, Korea; 12 International PhD Program in Biotech and Healthcare Management, College of Management, Taipei Medical University, Taipei, Taiwan; 13 Department of Internal Medicine, Hanyang University College of Medicine, Seoul, Korea; 14 College of Medicine, The Catholic University of Korea, Seoul, Korea; 15 Quality Use of Medicines and Pharmacy Research Centre, Clinical and Health Sciences, University of South Australia, Adelaide, Australia; 16 Department of Biomedical Informatics, Ajou University School of Medicine, Suwon, Korea; 17 Department of Biomedical Informatics, Ajou University School of Medicine, Suwon, Gyeonggi-do, Korea; 18 Department of Biostatistics, Fielding School of Public Health, University of California, Los Angeles; 19 VA Informatics and Computing Infrastructure, US Department of Veterans Affairs, Salt Lake City, Utah; 20 Medical Informatics Services, New York-Presbyterian Hospital, New York, New York; 21 Department of Internal Medicine, Hanyang University Guri Hospital, Hanyang University College of Medicine, Guri, Korea; 22 Pharmaco- and Device Epidemiology, Centre for Statistics in Medicine, Nuffield Department of Orthopaedics, Rheumatology, and Musculoskeletal Sciences, University of Oxford, Oxford, United Kingdom; 23 Department of Medical Informatics, Erasmus Medical Center University, Rotterdam, Netherlands

## Abstract

**Importance:**

Ranitidine, the most widely used histamine-2 receptor antagonist (H_2_RA), was withdrawn because of N-nitrosodimethylamine impurity in 2020. Given the worldwide exposure to this drug, the potential risk of cancer development associated with the intake of known carcinogens is an important epidemiological concern.

**Objective:**

To examine the comparative risk of cancer associated with the use of ranitidine vs other H_2_RAs.

**Design, Setting, and Participants:**

This new-user active comparator international network cohort study was conducted using 3 health claims and 9 electronic health record databases from the US, the United Kingdom, Germany, Spain, France, South Korea, and Taiwan. Large-scale propensity score (PS) matching was used to minimize confounding of the observed covariates with negative control outcomes. Empirical calibration was performed to account for unobserved confounding. All databases were mapped to a common data model. Database-specific estimates were combined using random-effects meta-analysis. Participants included individuals aged at least 20 years with no history of cancer who used H_2_RAs for more than 30 days from January 1986 to December 2020, with a 1-year washout period. Data were analyzed from April to September 2021.

**Exposure:**

The main exposure was use of ranitidine vs other H_2_RAs (famotidine, lafutidine, nizatidine, and roxatidine).

**Main Outcomes and Measures:**

The primary outcome was incidence of any cancer, except nonmelanoma skin cancer. Secondary outcomes included all cancer except thyroid cancer, 16 cancer subtypes, and all-cause mortality.

**Results:**

Among 1 183 999 individuals in 11 databases, 909 168 individuals (mean age, 56.1 years; 507 316 [55.8%] women) were identified as new users of ranitidine, and 274 831 individuals (mean age, 58.0 years; 145 935 [53.1%] women) were identified as new users of other H_2_RAs. Crude incidence rates of cancer were 14.30 events per 1000 person-years (PYs) in ranitidine users and 15.03 events per 1000 PYs among other H_2_RA users. After PS matching, cancer risk was similar in ranitidine compared with other H_2_RA users (incidence, 15.92 events per 1000 PYs vs 15.65 events per 1000 PYs; calibrated meta-analytic hazard ratio, 1.04; 95% CI, 0.97-1.12). No significant associations were found between ranitidine use and any secondary outcomes after calibration.

**Conclusions and Relevance:**

In this cohort study, ranitidine use was not associated with an increased risk of cancer compared with the use of other H_2_RAs. Further research is needed on the long-term association of ranitidine with cancer development.

## Key Points

**Question:**

Is use of ranitidine associated with higher risk for incident cancer compared with other histamine-2 (H_2_) receptor antagonists (H_2_RAs)?

**Findings:**

In this cohort study including 1 183 999 individuals from 11 large databases across Europe, North America, and Asia, risk of cancer among ranitidine users did not differ from users of other H_2_RAs. Ranitidine use was not associated with an increased risk of esophageal, stomach, or colorectal cancer, or 13 other subtypes of cancer.

**Meaning:**

These findings suggest that a history of ranitidine use is not associated with an increased risk of cancer compared with use of other H_2_ receptor antagonists, but further research is needed on the long-term effects of ranitidine on cancer development.

## Teaser

This cohort study examines the comparative risk of cancer associated with the use of ranitidine vs other histamine-2 receptor antagonists.

## Introduction

Ranitidine is a histamine-2 receptor antagonist (H_2_RA) that has been widely used to treat gastroesophageal reflux disease and peptic ulcer disease.^1^ In the US, more than 14 million ranitidine prescriptions were made annually in 2013 to 2018.^2^ In 2018, ranitidine was the third most prescribed gastrointestinal medication.^3^ In September 2019, the US Food and Drug Administration (FDA) found that some ranitidine medicines contained N-nitrosodimethylamine (NDMA), a known human carcinogen.^4,5^ In April 2020, the FDA requested manufacturers to withdraw all prescription and over-the-counter ranitidine-containing drugs, and the Committee for Medicinal Products for Human Use of the European Medicines Agency (EMA) recommended the suspension of all ranitidine medicines in the European Union. The FDA found that NDMA concentrations increased over time in some ranitidine products stored at temperatures higher than room temperature, resulting in exposure of consumers to unacceptable levels of the carcinogen^6^; the EMA also reached a similar conclusion.^7^

Given the worldwide popularity of ranitidine, exposure of several populations to NDMA and the potential risk of cancer development are important epidemiological concerns, and cancer screening may be required in individuals with previous prolonged exposure to ranitidine. However, the risk of cancer among individuals who used NDMA-contaminated ranitidine has not been fully evaluated. Although some studies have attempted to address this issue, the results are probably underpowered and lack generalizability due to using a single data source.^8,9,10^ Hence, a large-scale, multinational, multicenter cohort study was conducted to determine whether ranitidine use was associated with increased cancer risk. We aimed to generate robust evidence for the association of cancer development in adult individuals without previous cancer history with the use of ranitidine and other H_2_RAs.

## Methods

For this cohort study, each site received an institutional review board approval or obtained a waiver for the analysis of deidentified data according to institutional governance guidelines. The study is reported according to the Strengthening the Reporting of Observational Studies in Epidemiology (STROBE) reporting guideline.

### Study Design and Data Sources

This federated international network cohort study was facilitated by the Observational Health Data Sciences and Informatics open science collaboration.^11^ The eligible individuals were identified by reviewing the routinely collected data in electronic health records and health claim data from the US, the UK, Germany, France, Spain, South Korea, and Taiwan. All data sources were standardized based on the Observational Medical Outcomes Partnership common data model, version 5.3.^12^ On the basis of a network of standardized databases, a series of distributed network analyses were conducted per previous studies.^13,14^ In accordance with a prespecified statistical analysis plan, an end-to-end R package was developed and distributed across the participating databases. This executable analytical software package is available elsewhere.^15^ For interpretation and database-level meta-analysis, only predesigned tabular statistical results from the data sources without patient-level information were shared with the coordinating centers. The predefined study protocol was registered in the European Union Post-Authorisation Studies Register.

The contributing data sources were the IQVIA US Ambulatory Electronic Medical Research (AmbEMR; US), Columbia University Irving Medical Center data warehouse (CUIMC; US), Stanford Medicine Research Data Repository (STARR; US), UK’s IQVIA Medical Research Data (IMRD; UK), IQVIA Disease Analyzer Germany (DA Germany; Germany), Information System for Research in Primary Care (SIDIAP; Spain), IQVIA Longitudinal Patient Database (LPD; France), Korean National Health Insurance System–National Sample Cohort (NHIS-NSC; South Korea), Ajou University School of Medicine (AUSOM; South Korea), Kandong Sacred Heart Hospital (KDH; South Korea), Hanyang University Medical Center (HUMIC; South Korea), and Taipei Medical University Clinical Research Database (TMUCRD; Taiwan). A detailed description of the databases is available in eAppendix 1 in Supplement 1.

### Study Cohorts and Exposure

The study included adult patients aged 20 years or older who used ranitidine for more than 30 days with at least 1 year of exposure-free observation period prior to cohort entry. The comparator cohort was defined as adult patients who used other H_2_RAs (ie, famotidine, roxatidine, or lafutidine) with a 1-year washout period. Users of cimetidine and nizatidine were excluded from the primary comparator group, as previous studies have suggested that cimetidine may have anticancer effects^16^ and nizatidine is contaminated with NDMA.^17,18^ Patients with a history of cancer, exposure to other H_2_RAs for up to 1 year prior to cohort entry (for the target cohort), or ranitidine use for up to 1 year prior to cohort entry (for the comparator cohort) were excluded. Because of the use of ranitidine-containing combination drugs in Korea, patients exposed to sucralfate or bismuth within 1 month prior to the entry date were also excluded to minimize the imbalance between the ranitidine group and other H_2_RA groups.

The index date was defined as the date of H_2_RA treatment initiation. The end of the treatment duration was defined as the end of exposure to the drug of interest, allowing a 30-day gap between consecutive prescriptions. The study design is presented in eAppendix 2 in Supplement 1.

### Outcomes

The primary outcome was the development of any cancer types, excluding nonmelanoma skin cancer. Secondary outcomes included the development of any cancer types (including nonmelanoma skin cancer and excluding thyroid cancer) or the 16 cancer subtypes analyzed separately (breast cancer; prostate cancer; lung cancer; colorectal cancer; bladder cancer; liver cancer; leukemia; pancreatic cancer; stomach cancer; lip, oral cavity, and pharynx cancer; thyroid cancer; corpus uteri cancer; ovary cancer; esophageal cancer; gall bladder and biliary tract cancer; and cervix uteri cancer). A list of the diagnostic codes used for outcome ascertainment is provided in eAppendix 3 in Supplement 1.

### Statistical Analysis

Propensity score (PS) matching was performed to reduce potential confounding due to an imbalance in the baseline observed covariates between the target and the comparator cohorts. A large set of covariates was used to estimate the large-scale PSs, including age, sex, race, ethnicity, year and month of cohort entry, all recorded medications, medical history, procedures performed, and the Charlson Comorbidity Index score in the year prior to the index date. The information on race and ethnicity was derived from the databases. In AmbEMR, race was classified as Asian, White, and African American, with allowances for missing values; ethnicity was categorized as Hispanic or Latino, and Not Hispanic or Latino. Race and ethnicity were included in analysis because they may influence treatment decisions.

Comparator cohorts were constructed by performing 1:1 PS matching with a caliper of 0.2 SDs of the logic of the PS. Database-specific PSs were estimated using L_1_ regularized logistic regression tuned by 10-fold cross-validation.^19,20^ Cox proportional hazard models were fitted to estimate the hazard ratios (HRs) and 95% CIs according to exposure using the CohortMethod^21^ R package for each data source. A prespecified 2-sided *P* < .05 was considered significant.

For primary analysis, the result from each data source that passed the prespecified diagnostic criteria was aggregated using random-effects meta-analyses to calculate the summary HR.^22^ The study diagnostic criteria included empirical equipoise and sufficient balance of covariates after PS adjustment. Empirical equipoise was achieved if most patients’ preference scores (a transformation of the PS after adjusting for different prevalence of treatments) in both groups were between 0.3 and 0.7. If the absolute standardized mean difference of any covariate was greater than 0.1, the balance between the target and comparator cohorts was considered insufficient, and the analyses was excluded from primary meta-analysis.

Multiple sensitivity analyses were performed using different times of risk, definitions of the study population, outcomes, and statistical approaches. First, 4 different times of risk were defined: intention-to-treat (ITT), which followed patients until the end of data availability; ITT with a 1-year lag; during treatment, which followed people from 1 day after the index date until the completion of the treatment, allowing up to a 30-day gap between prescriptions; and during treatment with a 1-year lag. Second, 3 statistical models were applied in addition to the 1:1 PS matching: variable-ratio PS matching with a maximum ratio of 1:10, PS stratification into deciles, and unadjusted. The predefined setting for primary analysis was 1:1 PS matching with ITT and a 1-year lag. Third, negative control outcomes and empirical calibration were used to quantify and adjust for the impact of potential residual confounding due to unobserved covariates. Negative control outcomes were health events not causally associated with the target or comparator exposures, with an expected true HR equal to 1.^23^ A total of 119 negative control outcomes were considered (eAppendix 4 in Supplement 1). The comparative risks and CIs were empirically calibrated according to the empirical null distribution derived from the negative control outcomes.^24^ Fourth, for the analyses of the 18 secondary outcomes, statistical significance was reported after a Bonferroni correction to address multiplicity. Fifth, the interaction effects of cumulative drug dose were estimated.

All analyses were performed using R statistical software version 4.0.2 (R Project for Statistical Computing). Data were analyzed from April to September 2021.

## Results

### Cohort Selection

During the period from January 1986 to December 2020, a total of 1 183 999 study-eligible new users of ranitidine (909 168 individuals; mean age, 56.1 years; 507 316 [55.8%] women) or other H_2_RAs (274 831 individuals; mean age, 58.0 years; 145 935 [53.1%] women) were identified, including 758 683 individuals in US databases, 338 957 individuals in European databases, and 86 359 individuals in Asian databases. Among the 909 168 enrolled participants using ranitidine, the crude incidence rate of cancer was 14.30 events per 1000 person-years (PYs), and among 274 831 enrolled participants using other H_2_RAs, the crude incidence rate of cancer was 15.03 events per 1000 PYs. In the French database, other H_2_RA users were not identified; 590 cimetidine users corresponding to 6185 ranitidine users were identified.

### Cohort Characteristics

**Figure 1.**
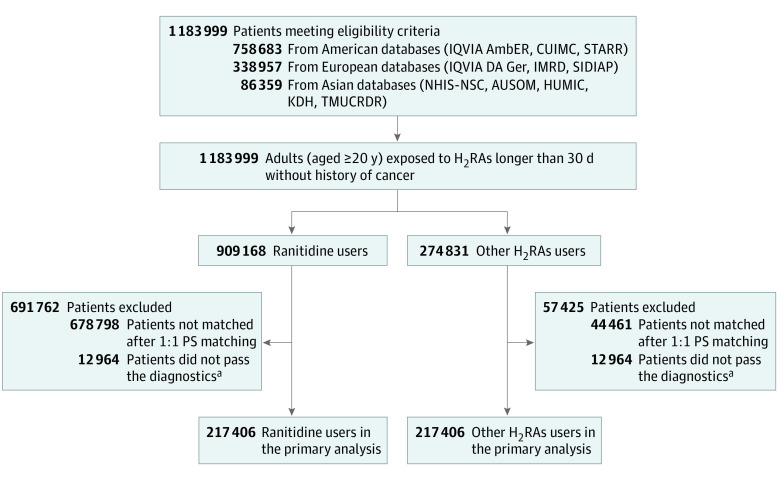
Patient Selection Flowchart AmbEMR indicates IQVIA US Ambulatory Electronic Medical Research; AUSOM, Ajou University School of Medicine; H_2_RA, histamine-2 receptor antagonist; CUIMC, Columbia University Irving Medical Center data warehouse; HUMIC, Hanyang University Medical Center; IMRD, UK’s IQVIA Medical Research Data; IQVIA DA Ger, IQVIA Disease Analyzer Germany; KDH, Kandong Sacred Heart Hospital; NHIS-NSC, Korean National Health Insurance System-National Sample Cohort; SIDIAP, The Information System for Research in Primary Care; STARR, Stanford University database warehouse; TMUCRD, Taipei Medical University Clinical Research Database. ^a^Diagnostic criteria indicate satisfaction of both empirical equipoise and balance after 1:1 propensity score (PS) matching; the study diagnostic criteria included empirical equipoise and sufficient balance of covariates after PS adjustment, and the empirical equipoise was satisfied when the preference scores of most patients (a transformation of the propensity score adjusting for different prevalence of treatments) in both groups were between 0.3 and 0.7. The balance between the target and comparator cohorts was considered sufficient if the absolute standardized mean difference of all covariates was not greater than 0.1.

The baseline characteristics of the participants from each database before and after large-scale PS matching are presented in eTables 1 through 12 in Supplement 1. After PS matching, 217 406 ranitidine users and 217 406 other H_2_RA users were included in the primary analysis from a total of 1 183 999 patients across the 11 databases, after excluding those whose results did not pass the study diagnostic criteria (Figure 1). No relevant imbalance (absolute standardized mean difference >0.1) was observed in the data from AmbEMR, CUIMC, SIDIAP, or NHIS-NSC, whereas empirical equipoise was identified in the data from AmbEMR, CUIMC, SIDIAP, NHIS-NSC, DA Germany, and IMRD. In the end, data from 4 databases (AmbEMR, CUIMC, SIDIAP, and NHIS-NSC) were included in the primary analysis. The cohort balance diagnostic criteria is presented in eFigure 1 in Supplement 1, and preference score distributions for each database are plotted in eFigure 2 in Supplement 1. Among 217 406 propensity-matched pairs in the primary analysis (mean age, 59.4 years; 139 921 [64.4%] women), 69 174 participants (31.8%) in the ranitidine group and 67 707 participants (31.1%) in the other H_2_RAs group had a history of gastroesophageal reflux disease, whereas 5657 participants (2.6%) in the ranitidine group and 5377 participants (2.5%) in the other H_2_RAs group had a history of peptic ulcer (Table 1).

**Table 1.** Baseline Characteristics of the Participants

Characteristics	Before propensity score matching			After propensity score matching^a^		
	Patients, No. (%)		SMD	Patients, No. (%)		SMD
	Ranitidine (n = 657 463)	Other H_2_RAs (n = 246 178)		Ranitidine (n = 217 406)	Other H_2_RAs (n = 217 406)	
Age, y^b^						
20-24	18 269 (2.8)	6799 (2.8)	<0.01	5356 (2.5)	5120 (2.4)	0.01
25-29	23 070 (3.5)	8052 (3.3)	0.01	6400 (2.9)	6197 (2.9)	0.01
30-34	30 365 (4.6)	9881 (4.0)	0.03	8222 (3.8)	8247 (3.8)	<0.01
35-39	34 837 (5.3)	10 208 (4.1)	0.05	8905 (4.1)	8870 (4.1)	<0.01
40-44	39 735 (6.0)	13 372 (5.4)	0.03	11 238 (5.2)	11 538 (5.3)	0.01
45-49	47 987 (7.3)	16 963 (6.9)	0.02	15 252 (7.0)	15 056 (6.9)	<0.01
50-54	58 191 (8.9)	21 366 (8.7)	0.01	18 560 (8.5)	19 189 (8.8)	0.01
55-59	66 452 (10.1)	25 527 (10.4)	0.01	23 064 (10.6)	22 704 (10.4)	0.01
60-64	71 306 (10.8)	27 207 (11.1)	0.01	24 158 (11.1)	24 474 (11.3)	<0.01
65-69	75 505 (11.5)	28 410 (11.5)	<0.01	26 153 (12.0)	25 787 (11.9)	0.01
70-74	77 917 (11.9)	29 668 (12.1)	0.01	28 329 (13.0)	28 447 (13.1)	<0.01
75-79	66 885 (10.2)	28 402 (11.5)	0.04	26 798 (12.3)	27 172 (12.5)	0.01
80-84	36 283 (5.5)	17 567 (7.1)	0.07	13 064 (6.0)	12 866 (5.9)	<0.01
Sex						
Women	428 309 (65.1)	157 259 (63.9)	0.03	139 921 (64.4)	139 949 (64.4)	<0.01
Men	229 154 (34.9)	88 919 (36.1)	0.03	77 485 (35.6)	77 255 (35.6)	<0.01
Medical history^c^						
Gastroesophageal reflux disease	215 696 (32.8)	71 368 (29.0)	0.08	69 174 (31.8)	67 707 (31.1)	0.01
Peptic ulcer disease	18 067 (2.7)	5717 (2.3)	0.03	5657 (2.6)	5377 (2.5)	0.01
Gastritis	59 891 (9.1)	20 314 (8.3)	0.03	18 329 (8.4)	18 272 (8.4)	<0.01
Crohn disease	2808 (0.4)	1184 (0.5)	0.01	1043 (0.5)	1032 (0.5)	<0.01
Chronic liver disease	10 193 (1.6)	4036 (1.6)	0.01	3408 (1.6)	3429 (1.6)	<0.01
Hypertensive disorder	23 6172 (40.0)	102 250 (41.5)	0.03	95 424 (43.9)	95 609 (44.0)	<0.01
Osteoarthritis	109 663 (16.7)	40 181 (16.3)	0.01	37 069 (17.1)	37 237 (17.1)	<0.01
Diabetes	105 928 (16.1)	42 193 (17.1)	0.03	39 256 (18.1)	38 982 (17.9)	<0.01
Depressive disorder	103 565 (15.8)	36 866 (15.0)	0.02	34 285 (15.8)	34 455 (15.8)	<0.01
Renal impairment	39 469 (6.0)	19 999 (8.1)	0.08	16 373 (7.5)	16 573 (7.6)	<0.01
Rheumatoid arthritis	10 504 (1.6)	4611 (1.9)	0.02	4152 (1.9)	4163 (1.9)	<0.01
Dementia	8710 (1.3)	3784 (1.5)	0.02	3095 (1.4)	3286 (1.5)	0.01
Medication use^d^						
Antiinflammatory and antirheumatic products	325 078 (49.4)	134 356 (54.6)	0.10	119 176 (54.8)	117 693 (54.1)	0.01
Lipid modifying agents	271 699 (41.3)	105 902 (43.0)	0.03	98 684 (45.4)	98 526 (45.3)	<0.01
Antidepressants	228 040 (34.7)	82 313 (33.4)	0.03	75 508 (34.7)	75 202 (34.6)	<0.01
Antithrombotic agents	201 383 (30.6)	89 912 (36.5)	0.13	80 699 (37.1)	81 078 (37.3)	<0.01
Immunosuppressants	24 361 (3.7)	12 322 (5.0)	0.06	10 219 (4.7)	10 043 (4.6)	<0.01

Abbreviations: H_2_RA, histamine-2 receptor antagonist; SMD, standardized mean difference.

^a^ To account for the baseline differences between groups, propensity score–based matching was performed. The propensity scores were calculated for each database independently based on the available demographic characteristics, as well as the medical parameters, medication use, procedure exposure history, and baseline laboratory values of each database. Here, we report the aggregated balance before and after matching only for limited covariates from the three databases. The balanced data before and after PS adjustment for more than 9000 baseline covariates in each database are available elsewhere.^15^

^b^ Participants aged older than 85 years were omitted.

^c^ Medical history was identified based on the coded medical diagnosis within 1 year prior to cohort entry.

^d^ Medication use was identified by reviewing the medication records within 1 year prior to cohort entry. Both Anatomical Therapeutic Chemical class-level and ingredient-level drug use were considered to fit the propensity score model. Only class-level balances of drugs before and after PS matching are reported in this table.

### Association of Ranitidine Use With Overall Cancer Risk, Except for Nonmelanoma Skin Cancer

**Figure 2.**
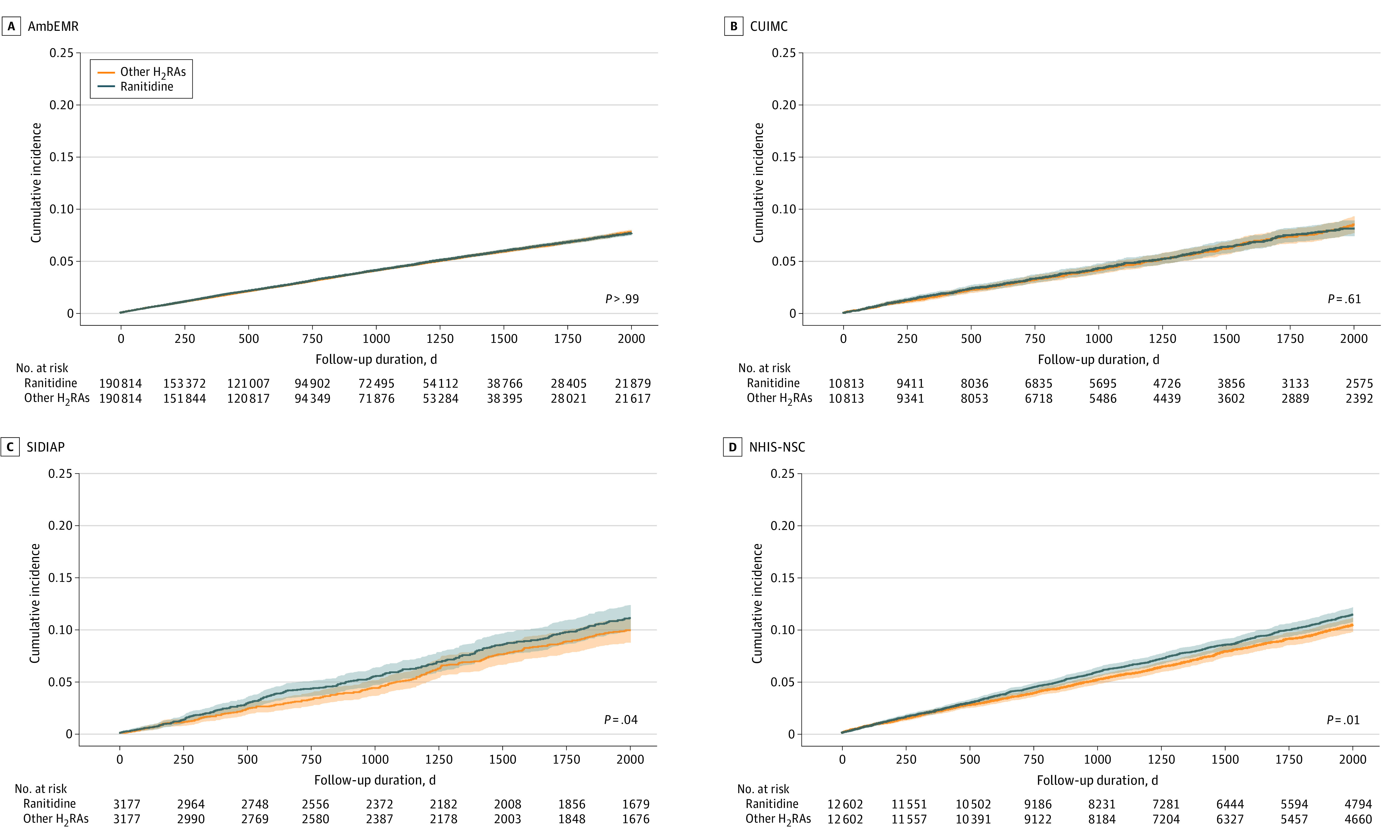
Kaplan-Meier Plots for the Risk of All Cancer Except Nonmelanoma Skin Cancer Associated With Ranitidine Users and Other Histamine-2 Receptor Antagonist (H_2_RA) Users *P*-values in survival curves were estimated using Cox proportional hazard regression models. Shading in the survival curves indicates 95% CIs. AmbEMR indicates IQVIA US Ambulatory Electronic Medical Research; CUIMC, Columbia University Irving Medical Center data warehouse; NHIS-NSC, Korean National Health Insurance System-National Sample Cohort; SIDIAP, The Information System for Research in Primary Care.

**Figure 3.**
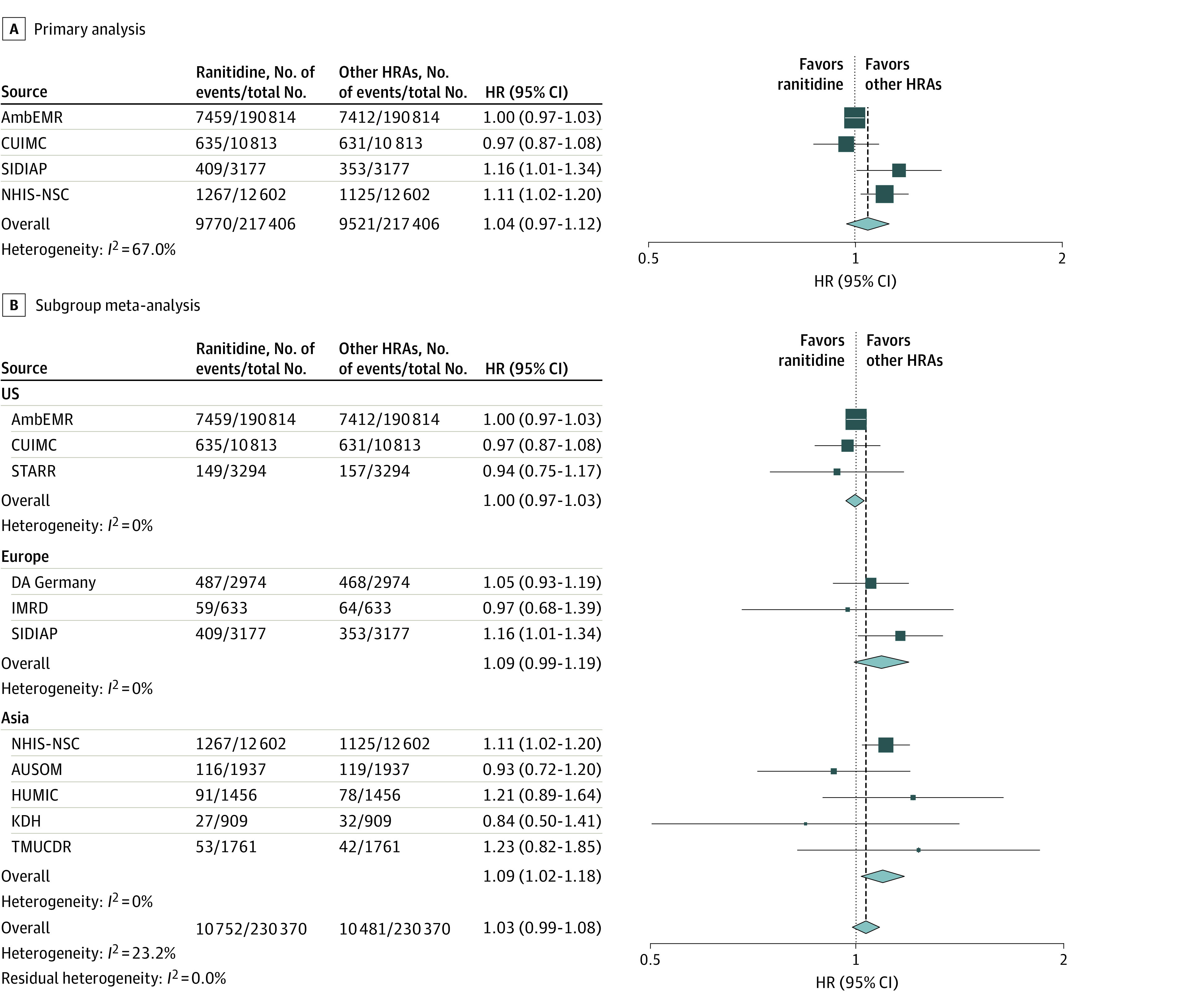
Risk of All Cancer Except Nonmelanoma Skin Cancer Between Ranitidine and Other H_2_ Receptor Antagonist (H_2_RA) Users in Meta-Analysis The summary hazard ratios were calculated using a random-effects model. A, Meta-analysis of results passing diagnostics (primary analysis). B, subgroup meta-analysis for primary outcome using results from all available data sources based on geographic region. Size of the data marker indicates the weight of the study; error bars, 95% CIs. AmbEMR indicates IQVIA US Ambulatory Electronic Medical Research; AUSOM, Ajou University School of Medicine; CUIMC, Columbia University Irving Medical Center data warehouse; DA Germany, IQVIA Disease Analyzer Germany; HUMIC, Hanyang University Medical Center; IMRD, UK’s IQVIA Medical Research Data; KDH, Kandong Sacred Heart Hospital; NHIS-NSC, Korean National Health Insurance System-National Sample Cohort; SIDIAP, The Information System for Research in Primary Care; STARR, Stanford University database warehouse; TMUCRD, Taipei Medical University Clinical Research Database.

The Kaplan-Meier curves for the primary outcome (overall cancer risk, except for nonmelanoma skin cancer) between ranitidine and other H_2_RA users are presented in Figure 2. No differences in risk were observed between the ranitidine and H_2_RA users in the AmbEMR (HR, 1.00; 95% CI: 0.97-1.03; mean follow-up, 2.6 years) and CUIMC (HR, 0.97; 95% CI, 0.87-1.08; mean follow-up, 3.6 years) databases (Figure 3A). Conversely, ranitidine users exhibited a higher risk of cancer than other H_2_RA users in the SIDIAP (HR, 1.16; 95% CI, 1.01-1.34; mean follow-up, 5.8 years) and NHIS-NSC (HR, 1.11; 95% CI, 1.02-1.20; mean follow-up, 4.5 years) databases. In the primary meta-analysis of the results from 4 databases, the hazard of cancer was not statistically significant (HR, 1.04; 95% CI, 0.97-1.12) (Figure 3A). The meta-analytic risk of the primary outcome across the 11 databases was similar (HR, 1.03; 95% CI, 0.99-1.08) (Figure 3B). Moreover, a subgroup meta-analysis of the primary outcomes for data from the US, Europe, and Asia was performed separately and found no difference in the risk of primary outcome between ranitidine users and other H_2_RA users in Western countries (US: HR, 1.00; 95% CI, 0.97-1.03; Europe: HR, 1.09; 95% CI, 0.99-1.19); however, the risk of primary outcome was higher among ranitidine users than in other H_2_RA users in Asian countries (HR, 1.09; 95% CI, 1.02-1.18) (Figure 3B).

Kaplan-Meier curves for the primary outcome between ranitidine users and other H_2_RA users who did not pass the study diagnostic criteria are presented in eFigure 3 in Supplement 1. In a subgroup meta-analysis stratifying results based on follow-up duration (eFigure 4 in Supplement 1), we observed no significant difference in the risk of primary outcome between ranitidine users and other H_2_RA users, regardless of whether the follow-up duration was 5 years or longer (HR, 1.08; 95% CI, 0.99-1.17) or less than 5 years (HR, 1.02; 95% CI, 0.96-1.08). Furthermore, there was no interaction between the cumulative dose of H_2_RAs and the comparative risk of the primary outcome between ranitidine vs other H_2_RAs users (eTable 13 in Supplement 1).

### Association of Ranitidine Use With Overall Cancer and Site-Specific Cancer Risk

The results for secondary outcomes are presented in Table 2 and eFigure 5 in Supplement 1. No significant differences were observed between ranitidine users and other H_2_RA users in terms of risk of all cancer types (excluding thyroid cancer) and 16 individual cancer subtypes after Bonferroni correction (Table 2).

**Table 2.** Cancer Risks for Ranitidine Users Compared With Other H_2_ Receptor Antagonist (H_2_RA) Users

Outcomes	Incidence, events per 1000 PYs		HR (95% CI)		Raw *P* value	Corrected *P* value^b^
	Ranitidine	Other H_2_RAs	Uncalibrated	Calibrated^a^		
All cancers excluding nonmelanoma skin cancer	15.92	15.65	1.04 (0.97-1.12)	1.02 (0.94-1.10)		
All cancers	18.11	17.82	1.04 (0.97-1.13)	1.02 (0.94-1.10)	.24	>.99
All cancers, excluding thyroid cancer	17.74	17.44	1.05 (0.97-1.12)	1.02 (0.94-1.10)	.21	>.99
Breast cancer	1.85	2.01	0.84 (0.69-1.02)	0.82 (0.67-0.99)	.08	>.99
Prostate cancer	1.83	1.87	0.98 (0.90-1.07)	0.96 (0.87-1.05)	.68	>.99
Lung cancer	1.45	1.36	1.07 (0.97-1.17)	1.04 (0.94-1.14)	.17	>.99
Colorectal cancer	1.12	1.11	1.00 (0.90-1.11)	0.97 (0.87-1.08)	.95	>.99
Bladder cancer	0.59	0.59	0.89 (0.68-1.16)	0.87 (0.66-1.13)	.40	>.99
Liver cancer	0.59	0.58	1.00 (0.86-1.15)	0.97 (0.84-1.12)	.95	>.99
Leukemia	0.52	0.54	1.12 (0.77-1.63)	1.09 (0.75-1.59)	.56	>.99
Pancreatic cancer	0.50	0.51	0.97 (0.79-1.18)	0.94 (0.77-1.15)	.75	>.99
Stomach cancer	0.37	0.29	1.17 (0.88-1.54)	1.13 (0.86-1.50)	.28	>.99
Lip, oral cavity, and pharynx cancer	0.27	0.28	1.00 (0.76-1.33)	0.98 (0.74-1.30)	.97	>.99
Thyroid cancer	<0.44^c^	<0.44^c^	1.01 (0.85-1.19)	0.98 (0.83-1.16)	.93	>.99
Corpus uteri cancer	<0.34^c^	0.28	1.20 (0.95-1.52)	1.17 (0.92-1.48)	.13	>.99
Ovary cancer	<0.26^c^	<0.21^c^	1.26 (1.00-1.58)	1.22 (0.97-1.54)	.05	.88
Esophageal cancer	<0.16^c^	0.15	1.08 (0.82-1.43)	1.05 (0.79-1.40)	.59	>.99
Gallbladder and biliary tract cancer	<0.16^c^	0.14	1.14 (0.86-1.53)	1.11 (0.83-1.49)	.36	>.99
Cervix uteri cancer	<0.11^c^	<0.13^c^	0.88 (0.64-1.22)	0.86 (0.62-1.19)	.45	>.99

Abbreviations: HR, hazard ratio; PY, person-year.

^a^ HRs and 95% CIs were empirically calibrated based on the results from the negative control outcome to address systematic bias.

^b^ *P* values were adjusted using the Bonferroni correction for multiple comparisons. The Bonferroni-corrected *P *value for multiple comparisons was not calculated for a single primary outcome.

^c^ If the number of outcomes was less than the prespecified minimum count (n = 5), the exact number was masked before aggregation to minimize the reidentification risk of a patient.

### Sensitivity Analyses of Comparison Between Ranitidine and Other H_2_RA Users

The analysis of negative control outcomes found that 107 of 113 ranitidine uses (94.7%) and 111 of 113 H_2_RA users (98.2%) had 95% CIs that covered 1 before and after empirical calibration (eFigure 6 in Supplement 1). The results of the sensitivity analyses with diverse time windows, 3 different PS adjustments, and additional empirical calibration to assess the robustness of the findings of the primary outcome analysis are shown in eFigure 7 in Supplement 1. After empirical calibration of the results, the significant positive associations between ranitidine use and cancer incidence based on the individual databases for the primary outcome were not maintained.

During treatment analysis revealed a significantly negative association between ranitidine use and the primary outcome in the AmbEMR database and a positive association in the NHIS-NSC database. Most results did not exhibit any significant associations after empirical calibration.

### Comparison of Risk of Primary Outcome Between Ranitidine and Individual H_2_RA Users

Only the results obtained from the analysis of the AmbEMR database passed the diagnostic criteria for comparison between ranitidine and cimetidine use. The risk for the primary outcome did not increase among ranitidine users compared with that among cimetidine users (HR, 1.02; 95% CI, 0.93-1.12) (eFigure 8 in Supplement 1). No significant difference was observed in the risk of the primary outcome between ranitidine and famotidine users (HR, 1.02; 95% CI, 0.98-1.06) and between ranitidine and nizatidine users (HR, 1.05; 95% CI, 0.94-1.17).

## Discussion

This cohort study found no consistent association of ranitidine use with cancer risk compared with use of other H_2_RAs. To our knowledge, this is the first international multidatabase study and the largest and most comprehensive analysis of the association between ranitidine use and cancer risk, including more than 900 000 users of ranitidine and more than 270 000 users of active comparators from the US, Europe, and Asia. Ranitidine users were compared with a large set of active comparators (H_2_RAs other than ranitidine as a group and individual H_2_RAs, including cimetidine, famotidine, lafutidine, nizatidine, and roxatidine), and the analyses elucidated robust findings using various statistical approaches (ITT vs during treatment, with or without lag period; 1:1, variable-ratio PS matching, or PS stratification), multiple cancer types, and composite outcomes.

Although ranitidine use was not associated with increased cancer risk in the meta-analysis, the results from South Korean and Spanish administrative data sources or the meta-analysis from Asia demonstrated an increase in cancer risk (<20%) in the ranitidine group compared with the other H_2_RAs group. This inconsistency in the results prevented us from ruling out a potential association between ranitidine use and cancer development, particularly in certain ethnic groups or health care systems. Nonetheless, the positive associations between ranitidine use and cancer incidence did not exist after empirical calibration was done to address systematic bias or after diverse sensitivity analyses with diverse balancing methods or time windows.

Dietary NDMA intake has been reported to be associated with an increased risk of gastrointestinal cancer, especially rectal cancer, in a prospective cohort study.^25^ In our large-scale multinational study, ranitidine use was not associated with an increased risk of esophageal, stomach, or colorectal cancers or the other 13 subtypes of cancer. These findings were also consistent with the results of 2 previous studies conducted in South Korea and the UK.^9,10^ They reported that ranitidine use was not associated with increased overall cancer risk compared with famotidine^10^ or omeprazole use.^9^ Although these studies have performed investigations similar to the current one, they were all limited by small sample sizes, short follow-up duration, and unmeasured bias due to limited covariate adjustments.

The observed lack of significant association between ranitidine use and cancer risk may be due to low levels of NDMA in ranitidine products. Although a large amount of NDMA would be harmful, NDMA levels in ranitidine found in preliminary tests conducted by the FDA barely exceeded the amount found in common foods.^4^ The results of 2 recent FDA studies^24,25^ support our findings. In vitro analyses revealed that ranitidine is not converted to NDMA in the gastric fluid under physiological conditions.^26^ Additionally, the oral administration of ranitidine did not increase the 24-hour urinary excretion of NDMA in healthy individuals.^27^ Similar levels of NDMA contamination were previously found in valsartan. In 2018, before the issue of NDMA-contaminated ranitidine was raised, the EMA announced that some valsartan products were contaminated with NDMA,^28^ and certain batches were recalled by many countries. A Danish nationwide cohort study found no association of NDMA-exposed valsartan use with incident cancer.^8^

Our study had several strengths. First, we included a large sample size of more than 1 000 000 participants from multiple countries and different data sources, thereby minimizing the risk of database-specific confounding and bias. Second, the use of active comparators and new user cohorts is an additional strength of this study. Third, robust methods were used to account for the confounding factors, including large-scale PS matching using several covariates. Fourth, the impact of unmeasured confounders and systematic errors was measured and reduced using negative control outcomes and empirical calibration, respectively. Fifth, the follow-up duration was relatively short. However, targeting only long-term or heavy users of H_2_RAs may lead to immortal and inevitable time bias.^29^ Furthermore, based on the results from the during treatment setting pertaining to only active drug users during follow-up, the risk for cancer did not differ between ranitidine and other H_2_RA users. Sixth, transparent distributed network analyses were performed following a predefined registered protocol, and all analytical codes were made publicly available under open science principles. This allows independent researchers to review our codes and replicate our findings. Additionally, these standardized analyses will allow rapid reanalysis involving the same or even more institutes once long-term follow-up data are available.

### Limitations

This study has several limitations. First, ranitidine use was determined based on the prescription or dispensation data rather than the data on actual use, including potential over-the-counter purchases. This could lead to information bias.^30^ However, a prospective study on ranitidine is no longer feasible since this drug has already been withdrawn from most markets worldwide. To evaluate the risk of cancer in individuals who have been exposed to ranitidine, a large-scale, retrospective, multinational cohort study is recognized as the criterion standard based on regulatory guidelines.^31^ Second, our study could not measure the NDMA levels derived from dietary sources, which may be a confounder or an effect modifier. The amount of NDMA in dietary sources varies depending on the geographic region and related dietary factors.^32^ However, our study included many participants from 3 different continents. Therefore, potential bias due to the unmeasured level of NDMA from dietary sources may be minimal, although existence of unmeasured confounder cannot be entirely excluded. Third, although this is the largest study to compare cancer risk between ranitidine users and active comparators to our knowledge, it may not be large enough to identify the subtle differences in cancer risk. Moreover, the EMA concluded that a 7-year daily intake of NDMA-contaminated valsartan may cause 1 additional case of cancer over the lifetime of 5000 people.^33^ The FDA had a similar conclusion (ie, a 4-year daily intake of NDMA-contaminated valsartan may cause 1 additional case of cancer over the lifetime of 8000 people).^34^ Fourth, the study period was relatively short and may underestimate the cancer risk associated with the longer-term use of ranitidine. Hence, future studies should perform a long-term follow-up. Fifth, the operational definition of cancer using diagnostic codes may introduce potential misclassification bias, although previous validation studies have shown generally consistent recording of cancer diagnoses.^35,36^

## Conclusion

The findings of this large international new-user active comparator cohort study found that despite known NDMA contamination in ranitidine, there was no statistically significant evidence that exposure to this drug was associated with increased risk of cancer. These findings do not support proactive cancer screening or surveillance among individuals previously exposed to ranitidine, and they may provide reassurance to previous ranitidine users. Nonetheless, further studies with longer follow-up periods are required to confirm these findings.

## Notes

Supplement 1. **eAppendix 1.** Data Source **eAppendix 2.** Graphical Overview of the Cohorts and Study Design **eAppendix 3.** Individual Outcome Definitions **eAppendix 4.** Falsification End Points **eTable 1.** Baseline Characteristics of Patients in the AmbEMR **eTable 2.** Baseline Characteristics of Patients in the CUIMC **eTable 3.** Baseline Characteristics of Patients in the STARR **eTable 4.** Baseline Characteristics of Patients in the DA Germany **eTable 5.** Baseline Characteristics of Patients in the IMRD **eTable 6.** Baseline Characteristics of Patients in the SIDIAP **eTable 7.** Baseline Characteristics of Patients in the NHIS-NSC **eTable 8.** Baseline Characteristics of Patients in the AUSOM **eTable 9.** Baseline Characteristics of Patients in the KDH **eTable 10.** Baseline Characteristics of Patients in the HUMIC **eTable 11.** Baseline Characteristics of Patients in the TMUCRD **eTable 12.** Study Population Before and After PS Matching **eTable 13.** Hazard Ratio of Subgroups Based on Cumulative Dose **eFigure 1.** Covariate Balance Plot Before and After Propensity Score Matching **eFigure 2.** Preference Score Distribution Before Propensity Score Matching **eFigure 3.** Kaplan-Meier Plots for Primary End Point Between Ranitidine and Other H_2_ Receptor Antagonists in Data Sources That Failed to Pass Diagnostics **eFigure 4.** Subgroup Meta-Analysis for Primary Outcome Using Results From All Available Data Sources Based on Follow-Up Duration **eFigure 5.** The Risk of the Secondary Outcomes Between Ranitidine and Other H_2_ Receptor Antagonists **eFigure 6.** Systematic Error Control of Effect Estimation in the Meta-Analysis **eFigure 7.** Sensitivity Analyses for Risk of Primary End Point Between Ranitidine and Other H_2_ Receptor Antagonists Using a Meta-Analysis and Various Time-at-Risk, Statistics, and Empirical Calibration **eFigure 8.** The Risk of the Primary Outcome (All Cancer Except Nonmelanoma Skin Cancer) Between Ranitidine and Cimetidine, Famotidine, and Nizatidine Users

Supplement 2.
Data Sharing Statement

## References

[1] Lipsy RJ, Fennerty B, Fagan TC. Clinical review of histamine2 receptor antagonists. Arch Intern Med. 1990;150(4):745-751. doi:10.1001/archinte.1990.00390160023006 1970232

[2] ClinCalc. Ranitidine: drug usage statistics, United States, 2013-2020. Accessed December 18, 2022. https://clincalc.com/DrugStats/Drugs/Ranitidine

[3] ClinCalc. The top 300 drugs of 2021. Accessed December 18, 2022. https://clincalc.com/DrugStats/Top300Drugs.aspx

[4] US Food and Drug Administration. Statement alerting patients and health care professionals of NDMA found in samples of ranitidine. News release. September 13, 2019. Accessed December 18, 2022. https://www.fda.gov/news-events/press-announcements/statement-alerting-patients-and-health-care-professionals-ndma-found-samples-ranitidine

[5] International Agency for Research on Cancer. Overall Evaluations of Carcinogenicity: An Updating of IARC Monographs Volumes 1 to 42. International Agency for Research on Cancer; 1987. 3482203

[6] US Food and Drug Administration. FDA requests removal of all ranitidine products (Zantac) from the market. News release. April 1, 2020. Accessed December 18, 2022. https://www.fda.gov/news-events/press-announcements/fda-requests-removal-all-ranitidine-products-zantac-market

[7] European Medicines Agency. Nitrosamine impurities. Accessed December 18, 2022. https://www.ema.europa.eu/en/human-regulatory/post-authorisation/referral-procedures/nitrosamine-impurities

[8] Pottegård A, Kristensen KB, Ernst MT, Johansen NB, Quartarolo P, Hallas J. Use of N-nitrosodimethylamine (NDMA) contaminated valsartan products and risk of cancer: Danish nationwide cohort study. BMJ. 2018;70(6):757-763. doi:10.1136/bmj.k3851 30209057 PMC6134800

[9] Kantor ED, O’Connell K, Du M, . Ranitidine use and cancer risk: results from UK Biobank. Gastroenterology. 2021;160(5):1856-1859.e5. doi:10.1053/j.gastro.2020.12.037 33385434 PMC8035224

[10] Yoon HJ, Kim J-H, Seo GH, Park H. Risk of cancer following the use of N-nitrosodimethylamine (NDMA) contaminated ranitidine products: a nationwide cohort study in South Korea. J Clin Med. 2021;10(1):153. doi:10.3390/jcm10010153 33466237 PMC7795144

[11] Hripcsak G, Duke JD, Shah NH, . Observational Health Data Sciences and Informatics (OHDSI): opportunities for observational researchers. Stud Health Technol Inform. 2015;216:574-578. 26262116 PMC4815923

[12] Overhage JM, Ryan PB, Reich CG, Hartzema AG, Stang PE. Validation of a common data model for active safety surveillance research. J Am Med Inform Assoc. 2012;19(1):54-60. doi:10.1136/amiajnl-2011-000376 22037893 PMC3240764

[13] You SC, Rho Y, Bikdeli B, . Association of ticagrelor vs clopidogrel with net adverse clinical events in patients with acute coronary syndrome undergoing percutaneous coronary intervention. JAMA. 2020;324(16):1640-1650. doi:10.1001/jama.2020.16167 33107944 PMC7592033

[14] Suchard MA, Schuemie MJ, Krumholz HM, . Comprehensive comparative effectiveness and safety of first-line antihypertensive drug classes: a systematic, multinational, large-scale analysis. Lancet. 2019;394(10211):1816-1826. doi:10.1016/S0140-6736(19)32317-7 31668726 PMC6924620

[15] You SC, Seo SI, Park CH. Ranitidine Cancer Risk. Accessed August 15, 2023. https://github.com/ohdsi-studies/RanitidineCancerRisk

[16] Kubecova M, Kolostova K, Pinterova D, Kacprzak G, Bobek V. Cimetidine: an anticancer drug? Eur J Pharm Sci. 2011;42(5):439-444. doi:10.1016/j.ejps.2011.02.004 21329756

[17] Iwagami M, Kumazawa R, Miyamoto Y, . Risk of cancer in association with ranitidine and nizatidine vs other h2 blockers: analysis of the Japan medical data center claims database 2005–2018. Drug Saf. 2021;44(3):361-371. doi:10.1007/s40264-020-01024-0 33247391

[18] US Food and Drug Administration. Laboratory tests—ranitidine. Accessed December 18, 2022. https://www.fda.gov/drugs/drug-safety-and-availability/laboratory-tests-ranitidine

[19] Suchard MA, Simpson SE, Zorych I, Ryan P, Madigan D. Massive parallelization of serial inference algorithms for a complex generalized linear model. ACM Trans Model Comput Simul. 2013;23(1):1-17. doi:10.1145/2414416.2414791 25328363 PMC4201181

[20] Tian Y, Schuemie MJ, Suchard MA. Evaluating large-scale propensity score performance through real-world and synthetic data experiments. Int J Epidemiol. 2018;47(6):2005-2014. doi:10.1093/ije/dyy120 29939268 PMC6280944

[21] CohortMethod. Accessed August 15, 2023. https://github.com/OHDSI/CohortMethod

[22] DerSimonian R, Laird N. Meta-analysis in clinical trials. Control Clin Trials. 1986;7(3):177-188. doi:10.1016/0197-2456(86)90046-2 3802833

[23] Lipsitch M, Tchetgen Tchetgen E, Cohen T. Negative controls: a tool for detecting confounding and bias in observational studies. Epidemiology. 2010;21(3):383-388. doi:10.1097/EDE.0b013e3181d61eeb 20335814 PMC3053408

[24] Schuemie MJ, Hripcsak G, Ryan PB, Madigan D, Suchard MA. Empirical confidence interval calibration for population-level effect estimation studies in observational healthcare data. Proc Natl Acad Sci U S A. 2018;115(11):2571-2577. doi:10.1073/pnas.1708282114 29531023 PMC5856503

[25] Loh YH, Jakszyn P, Luben RN, Mulligan AA, Mitrou PN, Khaw KT. N-nitroso compounds and cancer incidence: the European Prospective Investigation into Cancer and Nutrition (EPIC)–Norfolk Study. Am J Clin Nutr. 2011;93(5):1053-1061. doi:10.3945/ajcn.111.012377 21430112

[26] Gao Z, Karfunkle M, Ye W, . In vitro analysis of N-nitrosodimethylamine (NDMA) formation from ranitidine under simulated gastrointestinal conditions. JAMA Netw Open. 2021;4(6):e2118253. doi:10.1001/jamanetworkopen.2021.18253 34181009 PMC8239951

[27] Florian J, Matta MK, DePalma R, . Effect of oral ranitidine on urinary excretion of N-nitrosodimethylamine (NDMA): a randomized clinical trial. JAMA. 2021;326(3):240-249. doi:10.1001/jama.2021.9199 34180947 PMC8240005

[28] Farrukh MJ, Tariq MH, Malik O, Khan TM. Valsartan recall: global regulatory overview and future challenges. Ther Adv Drug Saf. Published online January 18, 2019. doi:10.1177/2042098618823458 30728946 PMC6351967

[29] Suissa S. Immortal time bias in pharmaco-epidemiology. Am J Epidemiol. 2008;167(4):492-499. doi:10.1093/aje/kwm324 18056625

[30] Pottegård A, Christensen Rd, Houji A, . Primary non-adherence in general practice: a Danish register study. Eur J Clin Pharmacol. 2014;70(6):757-763. doi:10.1007/s00228-014-1677-y 24756147

[31] European Network of Centres for Pharmacoepidemiology and Pharmacovigilance. ENCePP guide on methodological standards in pharmacoepidemiology. Accessed December 18, 2022. https://www.encepp.eu/standards_and_guidances/methodologicalGuide.shtml

[32] Tricker AR, Preussmann R. Carcinogenic N-nitrosamines in the diet: occurrence, formation, mechanisms and carcinogenic potential. Mutat Res. 1991;259(3-4):277-289. doi:10.1016/0165-1218(91)90123-4 2017213

[33] European Medicines Agency. Update on review of recalled valsartan medicines: preliminary assessment of possible risk to patients. News release. February 8, 2018. Accessed December 18, 2022. https://www.ema.europa.eu/en/news/update-review-recalled-valsartan-medicines-preliminary-assessment-possible-risk-patients

[34] US Food and Drug Administration. Laboratory analysis of valsartan products. Accessed December 18, 2022. https://www.fda.gov/drugs/drug-safety-and-availability/laboratory-analysis-valsartan-products

[35] Kao WH, Hong JH, See LC, . Validity of cancer diagnosis in the National Health Insurance database compared with the linked National Cancer Registry in Taiwan. Pharmacoepidemiol Drug Saf. 2018;27(10):1060-1066. doi:10.1002/pds.4267 28815803

[36] Dregan A, Moller H, Murray-Thomas T, Gulliford MC. Validity of cancer diagnosis in a primary care database compared with linked cancer registrations in England. Population-based cohort study. Cancer Epidemiol. 2012;36(5):425-429. doi:10.1016/j.canep.2012.05.013 22727737

