# Supplementary material for: Ranitidine Use and Incident Cancer in a Multinational Cohort
Source: JAMA Netw Open. 2023 Sep 19;6(9):e2333495. doi: 10.1001/jamanetworkopen.2023.33495 (PMC10509724; doi:10.1001/jamanetworkopen.2023.33495)
Supplement: Supplement 1. — eAppendix 1. Data Source eAppendix 2. Graphical Overview of the Cohorts and Study Design eAppendix 3. Individual Outcome Definitions eAppendix 4. Falsification End Points eTable 1. Baseline Characteristics of Patients in the AmbEMR eTable 2. Baseline Characteristics of Patients in the CUIMC eTable 3. Baseline Characteristics of Patients in the STARR eTable 4. Baseline Characteristics of Patients in the DA Germany eTable 5. Baseline Characteristics of Patients in the IMRD eTable 6. Baseline Characteristics of Patients in the SIDIAP eTable 7. Baseline Characteristics of Patients in the NHIS-NSC eTable 8. Baseline Characteristics of Patients in the AUSOM eTable 9. Baseline Characteristics of Patients in the KDH eTable 10. Baseline Characteristics of Patients in the HUMIC eTable 11. Baseline Characteristics of Patients in the TMUCRD eTable 12. Study Population Before and After PS Matching eTable 13. Hazard Ratio of Subgroups Based on Cumulative Dose eFigure 1. Covariate Balance Plot Before and After Propensity Score Matching eFigure 2. Preference Score Distribution Before Propensity Score Matching eFigure 3. Kaplan-Meier Plots for Primary End Point Between Ranitidine and Other H2 Receptor Antagonists in Data Sources That Failed to Pass Diagnostics eFigure 4. Subgroup Meta-Analysis for Primary Outcome Using Results From All Available Data Sources Based on Follow-Up Duration eFigure 5. The Risk of the Secondary Outcomes Between Ranitidine and Other H2 Receptor Antagonists eFigure 6. Systematic Error Control of Effect Estimation in the Meta-Analysis eFigure 7. Sensitivity Analyses for Risk of Primary End Point Between Ranitidine and Other H2 Receptor Antagonists Using a Meta-Analysis and Various Time-at-Risk, Statistics, and Empirical Calibration eFigure 8. The Risk of the Primary Outcome (All Cancer Except Nonmelanoma Skin Cancer) Between Ranitidine and Cimetidine, Famotidine, and Nizatidine Users [file jamanetwopen-e2333495-s001.pdf]

Supplemental Online Content

You SC, Seo SI, Falconer T, et al. Ranitidine use and incident cancer in a multinational cohort. *JAMA Netw Open*. 2023;6(9):e2333495. doi:10.1001/jamanetworkopen.2023.33495

eAppendix 1. Data Source
eAppendix 2. Graphical Overview of the Cohorts and Study Design
eAppendix 3. Individual Outcome Definitions
eAppendix 4. Falsification End Points
eTable 1. Baseline Characteristics of Patients in the AmbEMR
eTable 2. Baseline Characteristics of Patients in the CUIMC
eTable 3. Baseline Characteristics of Patients in the STARR
eTable 4. Baseline Characteristics of Patients in the DA Germany
eTable 5. Baseline Characteristics of Patients in the IMRD
eTable 6. Baseline Characteristics of Patients in the SIDIAP
eTable 7. Baseline Characteristics of Patients in the NHIS-NSC
eTable 8. Baseline Characteristics of Patients in the AUSOM
eTable 9. Baseline Characteristics of Patients in the KDH
eTable 10. Baseline Characteristics of Patients in the HUMIC
eTable 11. Baseline Characteristics of Patients in the TMUCRD
eTable 12. Study Population Before and After PS Matching
eTable 13. Hazard Ratio of Subgroups Based on Cumulative Dose
eFigure 1. Covariate Balance Plot Before and After Propensity Score Matching
eFigure 2. Preference Score Distribution Before Propensity Score Matching
eFigure 3. Kaplan-Meier Plots for Primary End Point Between Ranitidine and Other H2 Receptor Antagonists in Data Sources That Failed to Pass Diagnostics
eFigure 4. Subgroup Meta-Analysis for Primary Outcome Using Results From All Available Data Sources Based on Follow-Up Duration
eFigure 5. The Risk of the Secondary Outcomes Between Ranitidine and Other H2 Receptor Antagonists
eFigure 6. Systematic Error Control of Effect Estimation in the Meta-Analysis
eFigure 7. Sensitivity Analyses for Risk of Primary End Point Between Ranitidine and Other H2 Receptor Antagonists Using a Meta-Analysis and Various Time-at-Risk, Statistics, and Empirical Calibration
eFigure 8. The Risk of the Primary Outcome (All Cancer Except Nonmelanoma Skin Cancer) Between Ranitidine and Cimetidine, Famotidine, and Nizatidine Users

This supplemental material has been provided by the authors to give readers additional information about their work.

© 2023 You SC et al. *JAMA Network Open.*

eAppendix 1. Data Source

IQVIA US Ambulatory EMR (AmbEMR), US
The IQVIA US Ambulatory EMR database consists of longitudinal, de-identified electronic health records originating from ambulatory clients. The data contains detailed clinical information that captures important health outcomes such as lab test results and vital signs. It also covers administered drugs including prescription and over-the-counter medicines, vaccines, large-molecule biologic therapies, route of administration, days suppl and refill information.

Columbia University Irving Medical Center data warehouse (CUIMC), US
The clinical data warehouse of NewYork-Presbyterian Hospital/Columbia University Irving Medical Center, New York, NY (a major academic medical center) based on its current and previous electronic health record systems, with data spanning over 30 years and including over 6 million patients.

Stanford Medicine Research Data Repository (STARR), US
STAnford medicine Research data Repository, a clinical data warehouse containing live Epic data from Stanford Health Care (a major academic medical center), the Stanford Children's Hospital, the University Healthcare Alliance and Packard Children's Health Alliance clinics and other auxiliary data from Hospital applications such as radiology PACS. STARR platform is developed and operated by Stanford Medicine Research IT team and is made possible by Stanford School of Medicine Research Office.[1]

The Information System for Research In Primary Care (SIDIAP), Spain
Electronic health records from primary care partially linked to inpatient data. SIDIAP is also linked to a pharmacy dispensations and primary care laboratories. Healthcare is universal and taxpayer funded in the region, and primary care physicians are gatekeepers for all care and responsible for repeat prescriptions. This study was approved by the Clinical Research Ethics Committee of the IDIAPJGol (project code: 20/121-P)."

IQVIA Disease Analyzer Germany (DA Germany), Germany
Anonymized patient records collected from Patient Management software used by general practitioners and selected specialists to document patients' medical records within their officebased practice during a visit.

IQVIA UK Intergrated Medical Research Data (IMRD), UK
IMRD-UK contains primary care Electronic Medical Records from the UK. It incorporates data from THIN, A Cegedim Database. Reference made to THIN is intended to be descriptive of the data asset licensed by IQVIA.

IQVIA Longitudinal Patient Database France (LPD France), France
The IQVIA OMOP Longitudinal Patient Database France (LPD France) database consists of anonymized patient records collected from Patient Management software used by Doctors during an office visit to document patients' clinical records. The total database consists of 1200 GPs, 7.8 million patients, 620 specialists across 8 specialties (cardiology, neurology, psychiatry, pulmonology, gastroenterology, gynecology, diabetology & rheumatology)

Korean National Health Insurance System-National Sample Cohort (NHIS-NSC), South Korea
Korea National Health Insurance Service / National Sample Cohort (NHIS/NSC) is the national administrative claims database covering the South Korea population. It contains a 2% population sample cohort from 2002 - 2013.[2]

Ajou University School of Medicine (AUSOM), South Korea
The Ajou University School of Medicine (AUSOM) database consists of electronic health record data from a Korean tertiary teaching hospital with 1,108 patient beds, 33 medical departments, and 23 operating rooms. Ajou University Medical Center adopted a computerized provider order entry system in 1994 and a comprehensive electronic health record system in March 2010. The AUSOM database includes the medical records of 3.11 million patients collected from 1995-2019.[3]

Kandong Sacred Heart Hospital (KDH), South Korea
The Kangdong Sacred Heart Hospital (KDH) database consists of electronic health record data from Korean general hospital. The KDH database includes the medical records of 1.68 million patients collected from 2004-2019.

© 2023 You SC et al. *JAMA Network Open.*

Hanyang University Medical Center (HUMIC), South Korea
The Hanyang University Hospital (HUMIC) database consists of electronic health record data from Korean tertiary teaching hospital. The HUMIC database includes the medical records of 1.78 million patients collected from 2001-2018.

Taipei Medical University Clinical Research Database (TMUCRD)
Taipei Medical University has been integrating the electronic medical records databases of TMU's three affiliated hospitals (Taipei Medical University Hospital, Wanfang Hospital and Shuang Ho Hospital) to form the Taipei Medical University Clinical Research Database (TMUCRD) Since 2015. It combines various electronic medical records data of the three hospitals, including structured data (such as patient's basic information, medical information, test reports, diagnosis results, treatment procedures, surgery and medication status) and unstructured data (such as physicians records, pathology reports, medical imaging reports), and compiled them into analyzable data. The data period covered by TMUCRD is from 1998 to 2020. Since Shuang Ho Hospital joined the TMU System in 2008, the scope of the database includes the complete data of the three hospitals. The data content includes 13 categories, 63 data tables and 2,506 fields. In addition, the various data tables can be mutually linked. As of 2020, the database has accumulated the medical information of nearly 3.79 million patients across Taiwan.

© 2023 You SC et al. *JAMA Network Open.*

eAppendix 2. Graphical overview of the cohorts and study design

OHDSI's ATALS is an open source software tool (http://www.ohdsi.org/web/atlas/#/home) for researchers to conduct scientific analyses on standardized observational data converted to the OMOP Common Data model v5 or higher. Researchers can create cohorts by defining groups of people based on an exposure to a drug or diagnosis of a particular condition using healthcare data.

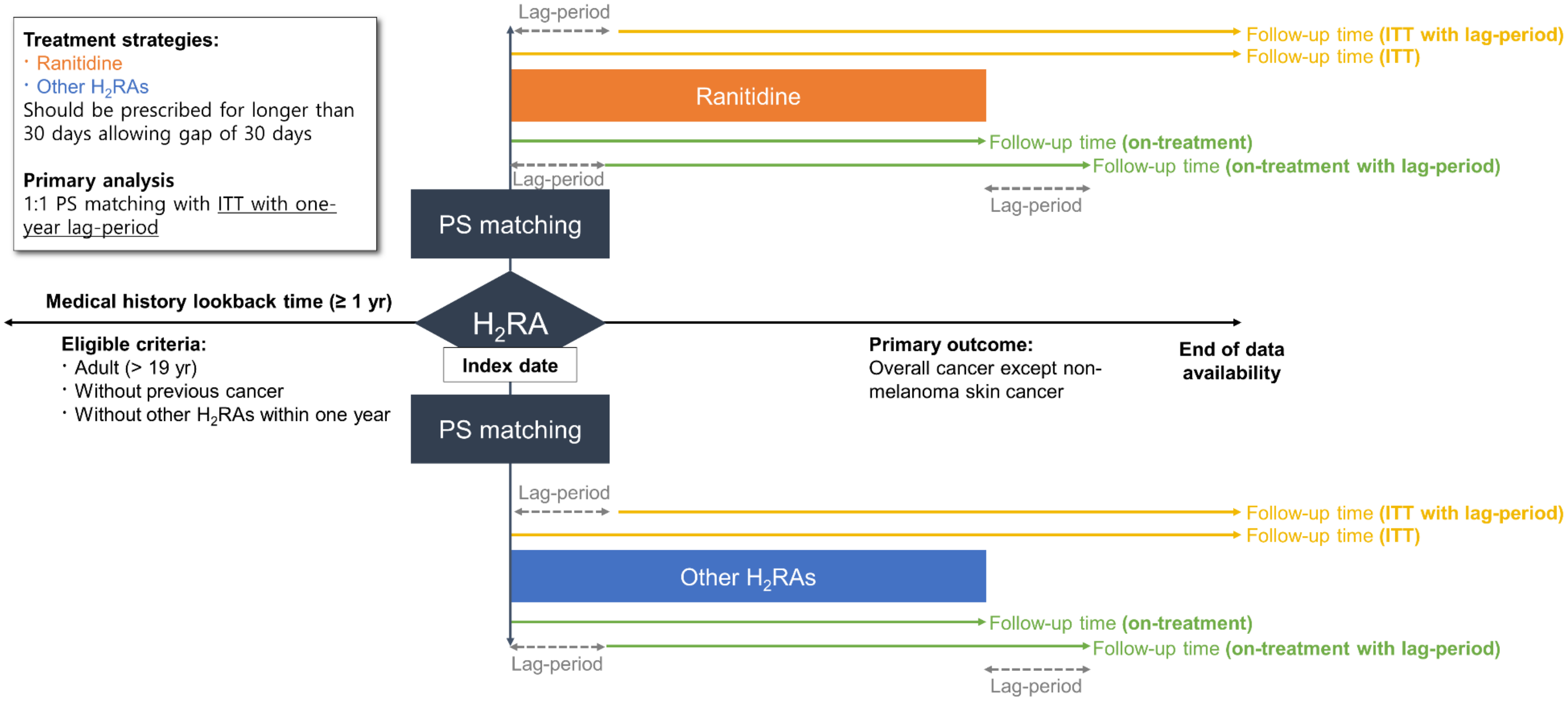

A parameterized SQL translation for cohort generation in any OMOP CDM v5 database is available in:https://github.com/ohdsi-studies/RanitidineCancerRisk/tree/master/inst/sql/sql_server

© 2023 You SC et al. *JAMA Network Open.*

eAppendix 3. Individual outcome definitions

From the GLOBOCAN estimates, we identified a wide array of 36 cancer types with varying incidence rates worldwide.[4] Among them, we constructed the definition of 16 cancer types for which observational data based on diagnosis codes, specifically the ICD-9-CM or ICD-10 codes, were deemed to be reliable and consistent. For each outcome, we developed an operational phenotype definition. Where possible, concept sets originated with published code lists (ICD-9-CM and ICD-10). We developed definition of outcome cohorts and query to extract them using ATLAS, the OHDSI open-source platform (https://github.com/OHDSI/atlas).

Outcome	ICD-9-CM	ICD-10
Overall cancer except non-melanoma skin cancer		
Overall cancer except thyroid cancer		
Overall cancer		
Lip, oral cavity and pharynx cancer	140-149; 160-161[5]	C00-C14[6]
Esophagus cancer	150[7]	C15[6]
Stomach cancer	151[7]	C16[6]
Colon and rectum cancer	153.x; 154.0-154.1, 154.8[8]	C18-C21[6]
Liver cancer	155[9,10]	C22[6]
Pancreas cancer	157[11]	C25[6]
Lung cancer	162.x[8,12]	C33-C34[6]
Breast cancer	174.x[8,12]	C50[6]
Cervix uteri cancer	180[12]	C53[6]
Corpus uteri cancer	182[13]	C54[6]
Ovary cancer	183[14]	C56[6]
Prostate cancer	185[12]	C61[6] [15]
Bladder cancer	188[16]	C67[6]
Leukemia	204-205[17]	C91-C95[6]
Thyroid cancer	193[18]	C73[15]
Gall bladder and biliary tract cancer	156[11]	C23-C24[15]

© 2023 You SC et al. *JAMA Network Open.*

eAppendix 4. Falsification endpoints

Falsification endpoints (Negative control outcomes) are concepts known to not be associated with the target or comparator cohorts, such that we can assume the true relative risk between the two cohorts is 1. Falsification endpionts are selected using a similar process to that outlined by Voss et al[19]. Once potential negative control candidates were selected, manual clinical review to exclude any pairs that may still be in a causal relationship or similar to the study outcome was be performed to select the top concepts by patient exposure. The final list of 119 negative outcomes is described below.

OMOP Concept ID	Outcome Name
443698	Abnormal anal Papanicolaou smear
443585	Abrasion and/or friction burn of multiple sites
380818	Acquired deformity of head
31668	Acquired deformity of neck
4319325	Acquired deformity of trunk
432411	Acquired equinus deformity of foot
439673	Acute hepatitis B with delta-agent (coinfection) without hepatic coma
441481	Adult victim of abuse
4218106	Alcoholism
4303805	Allergic reaction to bite and/or sting
4101660	Amputated below knee
4198962	Amputated thumb
4171556	Ankle ulcer
77650	Aseptic necrosis of bone
439237	Assault
141797	Black piedra
79232	Burn of ankle
4172458	Candidiasis of skin
42709838	Cellulitis of lower limb
439674	Chronic viral hepatitis B without delta-agent
4047787	Colles' fracture
134734	Compartment syndrome
72995	Contracture of joint of hand
80492	Contracture of knee joint
439666	Contracture of multiple joints
199978	Contusion of lower limb
433071	Contusion of multiple sites
201606	Crohn's disease
75389	Current tear of lateral cartilage AND/OR meniscus of knee
80242	Current tear of medial cartilage AND/OR meniscus of knee

© 2023 You SC et al. *JAMA Network Open.*

73575	Deformity of toe
436906	Disease caused by rickettsiae
4135080	Dislocation of radial head
78834	Effusion of joint of hand
4247710	Effusion of joint of pelvic region
72407	Effusion of joint of shoulder region
4150043	Epididymitis
197607	Excessive and frequent menstruation
374801	Foreign body in ear
4131595	Fracture of radius
441487	Frostbite
40481632	Ganglion cyst
74855	Genital herpes simplex
437744	Heat exhaustion
440021	Herpes simplex without complication
437489	Herpes zoster with complication
440329	Herpes zoster without complication
435511	Hypercalcemia
77364	Hypermobility of coccyx
74731	Hypertrophic osteoarthropathy
440129	Hypertrophy of nasal turbinates
440072	Hypogammaglobulinemia
4344500	Impingement syndrome of shoulder region
434872	Infection by Trichomonas
440053	Infestation by insect
4057662	Infestation by Phthirus
4168222	Intra-abdominal and pelvic swelling, mass and lump
72994	Jaccoud's syndrome
78512	Joint contracture of the ankle and/or foot
78228	Joint derangement
77072	Joint effusion of ankle AND/OR foot
72404	Joint stiffness
435903	Juvenile osteochondrosis of foot
438527	Juvenile osteochondrosis of lower extremity, excluding foot
435633	Juvenile osteochondrosis of upper extremity
4115991	Knee joint effusion
435516	Lipoprotein deficiency disorder

© 2023 You SC et al. *JAMA Network Open.*

4297984	Local infection of wound
440638	Lyme disease
438067	Malaria
438297	Mechanical complication of cardiac device, implant AND/OR graft
432798	Mechanical complication of internal orthopedic device, implant AND/OR graft
137967	Muscle, ligament and fascia disorders
4271024	Musculoskeletal fibromatosis
4209423	Nicotine dependence
201792	Nongonococcal urethritis
72413	Nontraumatic rupture of muscle
4215978	Onychomycosis
140648	Onychomycosis due to dermatophyte
4129408	Open wound of ankle
4053600	Open wound of elbow
77139	Open wound of finger without complication
444426	Open wound of foot except toes without complication
137426	Open wound of forearm without complication
77421	Open wound of hand except fingers without complication
4051004	Open wound of scalp
4129404	Open wound of upper arm
438120	Opioid dependence
4171915	Orchitis
315361	Orthopnea
74080	Orthostatic proteinuria
75920	Osteitis condensans
437359	Osteochondritis dissecans
378160	Otorrhea
77356	Pathological dislocation of joint
375292	Perforation of tympanic membrane
253796	Pneumothorax
4295261	Postmenopausal state
4094448	Pregnancy test negative
198715	Premature menopause
199876	Prolapse of female genital organs
4295888	Prolapse of intestine
194997	Prostatitis

© 2023 You SC et al. *JAMA Network Open.*

4245252	Raised prostate specific antigen
4345332	Spinal instability
4195698	Tenosynovitis
4339088	Testicular mass
80946	Tinea manus
4163280	Tinea of perianal region
133141	Tinea pedis
440268	Toxic effect of carbon monoxide
81930	Transient arthropathy
74719	Ulcer of foot
443593	Ulcer of thigh
4092565	Uterine prolapse
435131	Victim of neglect
261599	Vocal cord paralysis
132834	White piedra
435723	Wound seroma

© 2023 You SC et al. *JAMA Network Open.*

eTable 1. Baseline characteristics of patients in the AmbEMR

Characteristic	Before propensity score matching			After propensity score matching		
	Ranitidine	Other H_2RAs	Std. diff	Ranitidine	Other H_2RAs	Std. diff
Age, mean	58.3	59.7	0.09	59.9	59.9	<0.01
Age group[a]						
20 - 24	2.8	2.4	0.03	2.3	2.2	0.01
25 - 29	3.3	2.9	0.02	2.8	2.7	<0.01
30 - 34	4.1	3.7	0.02	3.6	3.6	<0.01
35 - 39	4.8	4.3	0.02	4.1	4.1	<0.01
40 - 44	5.6	5.1	0.02	4.9	5.0	<0.01
45 - 49	7.1	6.6	0.02	6.7	6.6	<0.01
50 - 54	8.9	8.5	0.01	8.3	8.6	0.01
55 - 59	10.5	10.5	<0.01	10.7	10.5	0.01
60 - 64	11.3	11.3	<0.01	11.2	11.4	0.01
65 - 69	12.1	11.9	0.01	12.3	12.1	<0.01
70 - 74	12.8	12.7	<0.01	13.6	13.7	<0.01
75 - 79	11.0	12.4	0.04	13.1	13.3	0.01
80 - 84	5.6	7.8	0.09	6.4	6.3	<0.01
Inclusion year						
2007	1.6	0.9	0.06	1.5	1.4	0.01
2008	1.8	1.1	0.06	1.7	1.6	<0.01
2009	2.8	1.6	0.08	2.6	2.4	0.01

© 2023 You SC et al. *JAMA Network Open.*

2010	3.3	2.3	0.06	3.4	3.4	<0.01
2011	4.5	3.3	0.06	5.0	4.8	0.01
2012	6.1	4.8	0.06	6.9	6.9	<0.01
2013	6.4	5.4	0.04	7.0	7.1	<0.01
2014	6.1	5.3	0.04	6.4	6.5	<0.01
2015	10.3	8.6	0.06	11.2	11.1	<0.01
2016	14.9	12.4	0.07	15.3	15.6	0.01
2017	14.4	12.5	0.06	14.4	14.5	<0.01
2018	13.8	11.4	0.07	12.6	12.5	<0.01
2020	1.9	17.4	0.54	0.3	0.3	0.01
Race[b]						
Asian	1.6	1.7	0.01	1.6	1.7	0.01
White	73.0	70.3	0.06	72.6	72.5	<0.01
African American	8.4	9.8	0.05	9.2	9.4	0.01
Ethnicity[c]						
Hispanic or Latino	0.8	0.7	0.01	0.8	0.8	<0.01
Not Hispanic or Latino	82.9	81.8	0.03	83.3	83.6	0.01
Gender						
Women	66.3	64.3	0.04	64.9	64.9	<0.01
Men	33.7	35.7	0.04	35.1	35.1	<0.01
Medical history[d]						
Gastroesophageal reflux disease	39.7	31.3	0.18	34.2	33.4	0.02

© 2023 You SC et al. *JAMA Network Open.*

Peptic ulcer	1.7	1.5	0.01	1.7	1.6	<0.01
Gastritis	4.9	4.5	0.02	4.7	4.6	<0.01
Crohn's disease	0.5	0.5	<0.01	0.5	0.5	<0.01
Chronic liver disease	1.2	1.2	<0.01	1.2	1.2	<0.01
Hypertensive disorder	41.9	42.7	0.02	45.1	45.2	<0.01
Osteoarthritis	15.9	16.6	0.02	17.3	17.4	<0.01
Diabetes mellitus	16.6	17.4	0.02	18.4	18.3	<0.01
Depressive disorder	17.3	15.9	0.04	16.7	16.8	<0.01
Renal impairment	6.3	8.4	0.08	8.0	8.1	<0.01
Rheumatoid arthritis	1.7	1.8	0.01	1.8	1.8	<0.01
Dementia	1.1	1.5	0.04	1.4	1.5	<0.01
Human immunodeficiency virus infection	0.2	0.3	0.02	0.3	0.3	0.01
Charlson comorbidity index, score	0.9	1.0	0.10	1.0	1.0	<0.01
Medication use[e]						
Antiinflammatory and antirheumatic products	49.2	54.7	0.11	54.9	54.2	0.01
Lipid modifying agents	44.7	46.0	0.03	48.2	48.1	<0.01
Antidepressants	38.9	36.1	0.06	37.2	37.0	<0.01
Antithrombotic agents	29.7	35.9	0.13	36.5	36.8	<0.01
Immunosuppressants	4.1	5.2	0.06	4.9	4.8	<0.01

Values are presented as proportion of the patients (%)unless otherwise indicated.

To account for baseline differences between the two groups, PS-based matching was used. PSs were calculated in each database independently, based on available demographic characteristics, as well as the medical, medication, procedure exposure history, and baseline laboratory values of each database. Here, we reported the aggregated balance before and after matching only for limited covariates from three databases. The whole balance data

© 2023 You SC et al. *JAMA Network Open.*

before and after PS adjustment for more than 9,000 baseline covariates in each database are available at https://github.com/OHDSI/ShinyDeploy/tree/master/RanitidineCancerRisk/data.

[a]Age groups over 85 were omitted.

[b]The race is reported based on the captured information in the database allowing missing values
[c]The ethnicity is reported based on the captured information in the database allowing missing values
[d]Medical history was identified by coded medical diagnosis within 1 year prior to the cohort entry.
[e]Medication use was identified by medication records within 1 year prior to the cohort entry. Both ATC class-level and ingredient-level drug uses were used to fit the PS model. The only class-level balances of drugs before and after PS matching is reported in this table.

Abbreviation: Std.diff, standardized difference; PS, propensity score; AmbEMR, IQVIA US Ambulatory EMR

© 2023 You SC et al. *JAMA Network Open.*

eTable 2. Baseline characteristics of patients in the CUIMC

Characteristic	Before propensity score matching			After propensity score matching		
	Ranitidine	Other H_2RAs	Std. diff	Ranitidine	Other H_2RAs	Std. diff
Age, mean	57.0	53.6	0.18	56.2	56.5	0.02
Age group[a]						
20 - 24	4.5	6.9	0.10	5.3	4.5	0.04
25 - 29	4.3	7.5	0.14	5.0	5.0	<0.01
30 - 34	5.3	6.8	0.06	5.6	5.6	<0.01
40 - 44	5.8	6.3	0.02	5.8	5.9	0.01
45 - 49	7.1	6.8	0.01	7.1	7.0	<0.01
50 - 54	8.9	8.6	0.01	8.7	9.4	0.02
55 - 59	10.3	9.0	0.04	9.8	9.8	<0.01
60 - 64	10.4	9.0	0.05	10.1	10.1	<0.01
65 - 69	10.9	8.9	0.07	10.3	10.3	<0.01
70 - 74	10.3	8.1	0.08	9.9	9.9	<0.01
75 - 79	7.4	6.2	0.05	7.3	7.2	<0.01
80 - 84	5.1	4.3	0.04	5.0	4.7	0.01
Gender						
Women	68.4	66.8	0.03	70.0	69.5	0.01
Men	31.6	33.2	0.03	30.0	30.5	0.01
Inclusion year						
1998	0.3	0.1	0.04	0.3	0.2	0.02

© 2023 You SC et al. *JAMA Network Open.*

2002	0.1	0.3	0.03	0.2	0.2	0.02
2003	0.5	0.8	0.03	0.7	0.9	0.02
2004	0.3	0.8	0.06	0.6	0.7	0.02
2005	0.3	0.6	0.04	0.4	0.7	0.04
2006	0.3	0.4	0.01	0.5	0.6	0.02
2007	0.8	0.5	0.03	1.1	0.8	0.03
2008	2.2	1.3	0.07	2.1	2.0	0.01
2009	2.5	1.9	0.04	2.5	2.1	0.03
2010	3.8	2.9	0.05	3.8	3.5	0.01
2011	6.7	6.3	0.02	6.7	6.5	0.01
2012	7.4	7.4	<0.01	7.7	7.4	0.01
2013	7.4	8.2	0.03	8.0	7.5	0.02
2014	7.5	8.8	0.05	7.9	8.0	<0.01
2015	10.7	10.6	<0.01	11.4	11.6	<0.01
2016	12.3	11.6	0.02	12.9	13.4	0.01
2017	13.2	11.6	0.05	13.0	14.0	0.03
2018	12.7	9.7	0.10	11.6	11.7	<0.01
2019	9.2	9.5	0.01	7.9	8.0	<0.01
Race[b]						
Asian	1.3	0.8	0.05	1.1	0.9	0.01
Black or African American	7.6	7.9	0.01	7.6	7.6	<0.01
White	28.0	22.9	0.12	26.4	26.7	0.01

© 2023 You SC et al. *JAMA Network Open.*

American Indian or Alaska Native	0.1	0.1	<0.01	0.1	0.1	0.01
Asian Indian	0.2	0.2	0.01	0.2	0.2	0.01
Laotian	0.6	0.5	0.01	0.7	0.5	0.02
Other Pacific Islander	0.8	0.5	0.03	0.7	0.7	0.01
Ethnicity[c]						
Hispanic or Latino	21.4	21.7	0.01	22.1	21.6	0.01
Not Hispanic or Latino	27.4	22.8	0.11	25.5	26.1	0.01
Medical history[d]						
Gastroesophageal reflux disease	22.9	18.3	0.11	20.9	21.6	0.01
Peptic ulcer	1.9	2.0	0.01	1.9	1.9	<0.01
Gastritis	5.0	6.7	0.07	5.5	5.3	0.01
Crohn's disease	0.4	0.5	<0.01	0.5	0.4	0.02
Chronic liver disease	2.3	3.2	0.05	2.3	2.4	0.01
Hypertensive disorder	37.6	36.1	0.03	38.0	38.3	<0.01
Osteoarthritis	14.5	13.3	0.03	15.1	15.0	<0.01
Diabetes mellitus	16.0	16.5	0.01	16.5	16.4	<0.01
Depressive disorder	10.0	10.6	0.02	11.1	11.2	<0.01
Renal impairment	9.8	10.3	0.02	7.0	7.2	0.01
Rheumatoid arthritis	1.2	1.1	<0.01	1.3	1.4	0.01
Dementia	1.5	1.9	0.03	1.7	1.7	<0.01
Charlson comorbidity index, score	1.7	1.8	0.04	1.7	1.7	<0.01
Medication use[e]						

© 2023 You SC et al. *JAMA Network Open.*

Antiinflammatory and antirheumatic products	47.7	51.4	0.07	51.6	51.2	0.01
Lipid modifying agents	34.9	31.4	0.07	34.1	34.5	0.01
Antidepressants	24.3	20.8	0.08	23.7	23.9	<0.01
Antithrombotic agents	30.0	33.7	0.08	31.2	30.7	0.01
Immunosuppressants	10.0	6.4	0.13	6.4	6.6	0.01

Values are presented as proportion of the patients (%) unless otherwise indicated.

To account for baseline differences between the two groups, PS-based matching was used. PSs were calculated in each database independently, based on available demographic characteristics, as well as the medical, medication, procedure exposure history, and baseline laboratory values of each database. Here, we reported the aggregated balance before and after matching only for limited covariates from three databases. The whole balance data before and after PS adjustment for more than 9,000 baseline covariates in each database are available at https://github.com/OHDSI/ShinyDeploy/tree/master/RanitidineCancerRisk/data.

[a]Age groups over 85 were omitted.

[b]The race is reported based on the captured information in the database allowing missing values
[c]The ethnicity is reported based on the captured information in the database allowing missing values
[d]Medical history was identified by coded medical diagnosis within 1 year prior to the cohort entry.
[e]Medication use was identified by medication records within 1 year prior to the cohort entry. Both ATC class-level and ingredient-level drug uses were used to fit the PS model. The only class-level balances of drugs before and after PS matching is reported in this table.

Abbreviation: Std.diff, standardized difference; PS, propensity score; CUIMC, Columbia University Irving Medical Center data warehouse

© 2023 You SC et al. *JAMA Network Open.*

eTable 3. Baseline characteristics of patients in the STARR

Characteristic	Before propensity score matching			After propensity score matching		
	Ranitidine	Other H_2RAs	Std. diff	Ranitidine	Other H_2RAs	Std. diff
Age, mean	52.9	51.3	0.09	52.8	53.2	0.02
Age group[a]						
20 - 24	5.5	6.4	0.04	6.3	5.4	0.04
25 - 29	5.9	7.2	0.05	6.3	6.6	0.01
30 - 34	7.5	8.9	0.05	7.5	7.6	<0.01
35 - 39	7.7	8.7	0.04	7.3	8.0	0.03
40 - 44	7.8	8.0	0.01	7.9	6.7	0.04
45 - 49	8.3	7.8	0.02	7.6	7.1	0.02
50 - 54	9.2	8.5	0.02	9.0	8.7	0.01
55 - 59	9.5	9.0	0.01	9.1	9.5	0.01
60 - 64	8.6	8.5	<0.01	8.7	9.3	0.02
65 - 69	9.9	8.2	0.06	9.1	9.7	0.02
70 - 74	7.7	7.2	0.02	8.1	8.7	0.02
75 - 79	6.0	5.1	0.04	6.1	5.9	0.01
80 - 84	3.9	3.7	0.01	4.0	3.7	0.02
Gender						
Women	64.7	62.8	0.04	67.1	67.5	0.01
Men	35.3	37.2	0.04	32.9	32.5	0.01
Inclusion year						

© 2023 You SC et al. *JAMA Network Open.*

2008	0.8	2.4	0.12	1.3	1.5	0.02
2009	2.5	3.9	0.07	3.4	2.8	0.04
2010	3.1	4.1	0.05	3.4	3.4	<0.01
2011	3.0	4.2	0.07	3.0	2.8	0.01
2012	4.8	4.1	0.04	4.0	3.7	0.01
2013	6.5	5.0	0.06	5.5	5.1	0.02
2014	9.2	5.7	0.14	6.7	7.0	0.01
2015	9.0	7.5	0.05	9.6	10.1	0.02
2016	14.5	8.6	0.18	13.9	14.2	0.01
2017	16.5	9.4	0.21	14.8	15.4	0.01
2018	16.8	11.8	0.14	15.8	16.7	0.02
2019	12.6	15.6	0.09	17.5	16.6	0.02
2020	0.7	17.8	0.62	1.0	0.8	0.02
Race[b]						
Asian	20.2	17.3	0.07	19.1	19.8	0.02
Black or African American	5.8	7.4	0.06	7.6	7.3	0.01
White	47.8	44.9	0.06	45.2	45.8	0.01
Native Hawaiian or Other Pacific Islander	1.1	1.5	0.04	1.2	1.3	<0.01
American Indian or Alaska Native	0.4	0.3	0.01	0.4	0.3	0.01
Ethnicity[c]						
Hispanic or Latino	15.0	22.2	0.18	17.0	16.5	0.01
Not Hispanic or Latino	77.5	73.7	0.09	75.8	76.1	0.01

© 2023 You SC et al. *JAMA Network Open.*

Medical history[d]						
Gastroesophageal reflux disease	49.3	29.5	0.41	41.8	44.2	0.05
Peptic ulcer	2.0	1.9	0.01	2.1	2.2	0.01
Gastritis	7.3	6.5	0.03	6.7	7.8	0.04
Crohn's disease	0.6	0.5	0.01	0.6	0.3	0.04
Chronic liver disease	2.2	3.5	0.08	2.2	2.4	0.02
Hypertensive disorder	32.6	36.1	0.07	35.9	35.5	0.01
Osteoarthritis	12.1	11.9	0.01	12.4	12.5	<0.01
Diabetes mellitus	12.0	16.5	0.13	13.9	14.0	<0.01
Depressive disorder	12.4	14.4	0.06	12.4	13.2	0.03
Renal impairment	5.5	16.9	0.36	7.0	6.6	0.01
Rheumatoid arthritis	1.5	1.5	<0.01	1.5	1.4	0.01
Dementia	0.8	1.4	0.05	1.1	1.1	<0.01
Charlson comorbidity index, score	1.0	1.5	0.26	1.1	1.0	0.03
Medication use[e]						
Antiinflammatory and antirheumatic products	18.8	21.1	0.06	20.6	20.4	0.01
Lipid modifying agents	21.3	23.1	0.04	23.9	23.6	0.01
Antidepressants	18.2	20.0	0.04	18.8	18.3	0.01
Antithrombotic agents	11.5	31.5	0.50	14.8	13.0	0.05
Immunosuppressants	5.1	11.7	0.24	5.6	4.9	0.03

Values are presented as proportion of the patients (%) unless otherwise indicated.

To account for baseline differences between the two groups, PS-based matching was used. PSs were calculated in each database independently, based on available demographic characteristics, as well as the medical, medication, procedure exposure history, and baseline laboratory values of each

© 2023 You SC et al. *JAMA Network Open.*

database. Here, we reported the aggregated balance before and after matching only for limited covariates from three databases. The whole balance data before and after PS adjustment for baseline covariates in each database are available at https://github.com/OHDSI/ShinyDeploy/tree/master/RanitidineCancerRisk/data.

[a]Age groups over 85 were omitted.

[b]The race is reported based on the captured information in the database allowing missing values
[c]The ethnicity is reported based on the captured information in the database allowing missing values
[d]Medical history was identified by coded medical diagnosis within 1 year prior to the cohort entry.
[e]Medication use was identified by medication records within 1 year prior to the cohort entry. Both ATC class-level and ingredient-level drug uses were used to fit the PS model. The only class-level balances of drugs before and after PS matching is reported in this table.

Abbreviation: Std.diff, standardized difference; PS, propensity score; STARR, Stanford Medicine Research Data Repository

© 2023 You SC et al. *JAMA Network Open.*

eTable 4. Baseline characteristics of patients in the DA Germany

Characteristic	Before propensity score matching			After propensity score matching		
	Ranitidine	Other H_2RAs	Std. diff	Ranitidine	Other H_2RAs	Std. diff
Age, mean	58.9	57.9	0.06	58.0	58.1	0.01
Age group[a]						
20 - 24	2.1	2.1	<0.01	2.4	2.0	0.03
25 - 29	2.8	2.3	0.03	2.2	2.1	<0.01
30 - 34	3.9	3.5	0.02	3.8	3.4	0.02
35 - 39	4.8	5.0	0.01	5.0	4.9	<0.01
40 - 44	6.4	7.9	0.06	7.4	7.7	<0.01
45 - 49	7.9	8.1	0.01	8.7	8.2	0.02
50 - 54	9.5	10.8	0.04	10.5	10.9	0.01
55 - 59	10.8	11.0	0.01	10.1	10.8	0.02
60 - 64	11.2	11.8	0.02	11.3	11.8	0.02
65 - 69	11.6	13.2	0.05	12.4	13.6	0.04
70 - 74	11.2	10.7	0.02	11.8	11.0	0.02
75 - 79	9.4	7.2	0.08	8.0	7.5	0.02
80 - 84	5.6	4.5	0.05	4.8	4.5	0.02
Gender						
Women	58.3	56.4	0.04	54.0	56.2	0.04
Men	41.7	43.6	0.04	46.0	43.8	0.04
Inclusion year						

© 2023 You SC et al. *JAMA Network Open.*

1993	1.1	6.6	0.29	9	6.9	0.08
1994	0.8	5.2	0.26	6.4	5.5	0.04
1995	0.9	3.0	0.15	3.9	3.3	0.04
1996	2.1	2.8	0.04	3.4	3.3	0.01
1997	2.5	1.6	0.06	2	1.8	0.02
1998	2.8	1.5	0.09	1.8	1.7	0.01
1999	2.4	0.8	0.13	0.8	0.9	0.01
2000	2.3	2.1	0.01	2.6	2.4	0.01
2001	1.9	2.1	0.02	2.3	2.4	0.01
2002	2.6	4.7	0.11	5.9	5.4	0.02
2003	3.8	4.9	0.06	5.3	5.6	0.01
2004	3.8	5.0	0.06	5.2	5.8	0.03
2005	4.4	5.4	0.04	6.1	6.3	0.01
2006	5.4	5.4	<0.01	6.1	6.2	0.01
2007	5.1	4.4	0.04	4.3	5.1	0.04
2008	4.1	3.5	0.03	2.9	3.9	0.05
2009	4.2	2.8	0.08	3.1	3.3	0.01
2010	3.8	3.1	0.04	2.6	3.3	0.04
2011	4.1	2.3	0.10	2.7	2.6	<0.01
2012	4.2	3.0	0.06	3.0	3.4	0.03
2013	4.1	3.5	0.03	3.4	4.0	0.04
2014	4.4	2.9	0.08	2.9	3.3	0.02

© 2023 You SC et al. *JAMA Network Open.*

2015	4.0	1.8	0.13	2.4	2.1	0.02
2016	5.9	2.1	0.19	2.4	2.5	<0.01
2017	6.4	2.2	0.21	4.6	2.6	0.11
2018	6.5	2.2	0.21	1.8	2.3	0.03
2019	5.8	3.7	0.10	2.3	3.0	0.04
2020	0.6	11.3	0.46	1.0	1.1	0.01
Medical history[b]						
Gastroesophageal reflux disease	6.4	6.2	0.01	5.1	5.5	0.02
Peptic ulcer	5.6	8.4	0.11	11.4	8.8	0.09
Gastritis	34.2	32.5	0.04	33.7	33.5	<0.01
Crohn's disease	0.4	0.4	<0.01	0.3	0.4	0.03
Chronic liver disease	0.6	0.6	<0.01	0.6	0.6	<0.01
Hypertensive disorder	31.4	26.2	0.12	26.3	27.9	0.04
Osteoarthritis	13.1	13.1	<0.01	14.4	13.9	0.01
Diabetes mellitus	11.5	10.2	0.04	10.8	10.8	<0.01
Depressive disorder	11.9	11.1	0.03	10.8	10.6	<0.01
Renal impairment	2.7	2.2	0.03	1.8	2.1	0.02
Dementia	1.7	1.0	0.06	1.0	0.9	0.01
Charlson comorbidity index, score	1.3	1.2	0.09	1.2	1.1	0.02
Medication use[c]						
Antiinflammatory and antirheumatic products	48.4	45.1	0.07	46.7	46.5	<0.01
Lipid modifying agents	20.5	17.5	0.08	17.2	18.0	0.02

© 2023 You SC et al. *JAMA Network Open.*

Antidepressants	13.2	13.2	<0.01	12.0	12.9	0.03
Antithrombotic agents	24.4	24.1	0.01	24.9	24.3	0.01
Immunosuppressants	1.5	1.7	0.01	1.4	1.6	0.02

Values are presented as proportion of the patients (%) unless otherwise indicated.

To account for baseline differences between the two groups, PS-based matching was used. PSs were calculated in each database independently, based on available demographic characteristics, as well as the medical, medication, procedure exposure history, and baseline laboratory values of each database. Here, we reported the aggregated balance before and after matching only for limited covariates from three databases. The whole balance data before and after PS adjustment for baseline covariates in each database are available at https://github.com/OHDSI/ShinyDeploy/tree/master/RanitidineCancerRisk/data.

[a]Age groups over 85 were omitted.

[b]Medical history was identified by coded medical diagnosis within 1 year prior to the cohort entry.
[c]Medication use was identified by medication records within 1 year prior to the cohort entry. Both ATC class-level and ingredient-level drug uses were used to fit the PS model. The only class-level balances of drugs before and after PS matching is reported in this table.

Abbreviation: Std.diff, standardized difference; PS, propensity score; DA Germany, IQVIA Disease Analyzer Germany

© 2023 You SC et al. *JAMA Network Open.*

eTable 5. Baseline characteristics of patients in the IMRD

Characteristic	Before propensity score matching			After propensity score matching		
	Ranitidine	Other H_2RAs	Std. diff	Ranitidine	Other H_2RAs	Std. diff
Age in year	59.8	62.2	0.15	60.2	61.8	0.10
Age group[a]						
20 - 24	2.2	0.9	0.11	1.4	0.9	0.04
25 - 29	3.2	0.9	0.17	2.1	0.9	0.09
30 - 34	4.4	1.9	0.14	3.8	1.7	0.13
35 - 39	5.4	5.0	0.02	5.5	5.1	0.02
40 - 44	6.2	4.0	0.10	6.8	4.4	0.10
45 - 49	7.3	7.7	0.02	7.9	7.7	0.01
50 - 54	8.3	8.7	0.02	8.4	9.0	0.02
55 - 59	9.1	11.3	0.07	9.5	11.5	0.07
60 - 64	10.5	12.0	0.05	10.7	12.0	0.04
65 - 69	11.0	12.9	0.06	10.0	13.1	0.10
75 - 79	8.9	10.5	0.05	9.0	10.3	0.04
80 - 84	6.8	7.7	0.04	7.6	7.1	0.02
Gender						
Women	59.2	52.4	0.14	49.3	53.1	0.08
Women	40.8	47.6	0.14	50.7	46.9	0.08
Inclusion year						
1996	0.1	<0.8	0.06	<0.8	<0.8	0.03

© 2023 You SC et al. *JAMA Network Open.*

1997	1.0	3.3	0.16	3.9	3.5	0.03
1998	1.8	5.4	0.19	5.8	5.7	0.01
1999	2.2	6.6	0.22	4.4	6.2	0.08
2000	2.9	6.6	0.17	6.6	7.3	0.03
2001	4.4	9.0	0.18	10.7	8.8	0.06
2002	5.1	10.3	0.20	9.2	10.1	0.03
2003	5.7	6.7	0.04	10.6	7.0	0.13
2004	5.0	6.9	0.08	6.8	7.4	0.03
2005	4.5	6.4	0.09	4.7	7.1	0.10
2006	4.0	7.3	0.14	6.8	7.4	0.03
2007	3.7	3.6	0.00	2.8	3.5	0.04
2008	3.9	4.0	0.00	4.6	4.3	0.01
2009	5.8	4.7	0.05	4.7	4.4	0.01
2010	5.4	4.9	0.02	3.3	5.2	0.09
2011	4.9	3.9	0.05	3.6	3.8	0.01
2012	5.4	3.7	0.08	3.2	2.7	0.03
2013	6.0	1.3	0.25	1.7	1.1	0.05
2014	6.3	1.9	0.23	1.9	1.7	0.01
2015	6.3	1.4	0.26	1.4	1.3	0.01
2016	5.8	<0.8	0.30	1.3	<0.8	0.09
2017	5.7	<0.8	0.31	1.1	<0.8	0.07
2018	4.0	<0.8	0.22	<0.8	<0.8	0.03

© 2023 You SC et al. *JAMA Network Open.*

Race[b]						
White	26.8	21.5	0.12	19	22.0	0.07
Asian Indian	0.4	<0.8	0.02	<0.8	<0.8	0.02
Chinese	0.1	<0.8	0.07	<0.8	<0.8	0.03
Pakistani	0.3	<0.8	0.03	<0.8	<0.8	0.06
Black	0.2	<0.8	0.02	<0.8	<0.8	0.03
Medical history[c]						
Gastroesophageal reflux disease	3.1	2.1	0.06	2.5	2.2	0.02
Gastritis	3.9	2.4	0.08	2.7	2.1	0.04
Hypertensive disorder	3.4	4.4	0.06	3.2	4.7	0.08
Osteoarthritis	4.0	4.3	0.01	4.4	4.6	0.01
Diabetes mellitus	2.9	3.0	0.01	2.1	2.8	0.05
Depressive disorder	3.7	2.4	0.07	2.5	2.4	0.01
Hyperlipidemia	1.3	2.9	0.11	1.4	3.0	0.11
Renal impairment	2.8	1.6	0.09	1.7	1.6	0.01
Rheumatoid arthritis	0.5	<0.8	0.03	<0.8	<0.8	0.03
Charlson comorbidity index, score	1.0	0.9	0.12	0.8	0.9	0.03
Medication use[d]						
Antiinflammatory and antirheumatic products	34.1	31.1	0.06	28.8	31.1	0.05
Lipid modifying agents	29.1	23.8	0.12	19.1	22.4	0.08
Antidepressants	28.4	21.5	0.16	17.5	21.2	0.09
Antithrombotic agents	30.3	26.6	0.08	23.9	25.1	0.03

© 2023 You SC et al. *JAMA Network Open.*

Immunosuppressants	2.6	1.6	0.07	1.7	1.3	0.04

Values are presented as proportion of the patients (%) unless otherwise indicated.

To account for baseline differences between the two groups, PS-based matching was used. PSs were calculated in each database independently, based on available demographic characteristics, as well as the medical, medication, procedure exposure history, and baseline laboratory values of each database. Here, we reported the aggregated balance before and after matching only for limited covariates from three databases. The whole balance data before and after PS adjustment for baseline covariates in each database are available at https://github.com/OHDSI/ShinyDeploy/tree/master/RanitidineCancerRisk/data.

[a]Age groups over 85 were omitted.

[b]The race is reported based on the captured information in the database allowing missing values
[c]Medical history was identified by coded medical diagnosis within 1 year prior to the cohort entry.
[d]Medication use was identified by medication records within 1 year prior to the cohort entry. Both ATC class-level and ingredient-level drug uses were used to fit the PS model. The only class-level balances of drugs before and after PS matching is reported in this table.

Abbreviation: Std.diff, standardized difference; PS, propensity score; IMRD, UK's IQVIA Medical Research Data

© 2023 You SC et al. *JAMA Network Open.*

eTable 6. Baseline characteristics of patients in the SIDIAP

Characteristic	Before propensity score matching			After propensity score matching		
	Ranitidine	Other H_2RAs	Std. diff	Ranitidine	Other H_2RAs	Std. diff
Age in year	57.6	63.1	0.31	65.6	65.1	0.03
Age group[a]						
20 - 24	2.4	1.7	0.05	1.3	1.0	0.02
25 - 29	4.2	1.9	0.13	1.2	1.2	<0.01
30 - 34	6.6	2.5	0.19	1.7	2.1	0.03
35 - 39	7.8	4.3	0.15	3.5	3.6	<0.01
40 - 44	7.2	5.3	0.08	2.8	3.9	0.06
45 - 49	7.1	6.3	0.03	5.5	5.3	0.01
50 - 54	7.6	7.4	<0.01	6.5	7.1	0.02
55 - 59	8.1	8.5	0.01	8.3	8.6	0.01
60 - 64	9.1	10.6	0.05	11.7	11.2	0.01
65 - 69	9.6	11.2	0.05	12.5	12.6	<0.01
70 - 74	9.1	11.0	0.06	11.4	11.1	0.01
75 - 79	8.3	11.2	0.10	14.4	12.9	0.04
80 - 84	6.7	9.2	0.09	9.8	10.6	0.03
Gender						
Women	62.5	54.7	0.16	50.6	51.6	0.02
Men	37.5	45.3	0.16	49.4	48.4	0.02
Inclusion year						

© 2023 You SC et al. *JAMA Network Open.*

2007	4.6	7.0	0.10	10.6	9.8	0.03
2008	6.0	7.7	0.06	10.6	11.0	0.01
2009	6.6	6.4	0.01	9.0	9.3	0.01
2010	9.0	13.2	0.13	20.7	19.0	0.04
2011	6.9	8.4	0.06	13.6	12.5	0.03
2012	6.3	3.8	0.11	5.3	5.9	0.03
2013	6.7	3.7	0.14	5.0	5.6	0.03
2014	8.7	3.5	0.22	5.2	5.4	0.01
2015	9.2	3.1	0.26	4.2	4.8	0.03
2016	9.4	3.5	0.24	4.9	5.3	0.02
2017	9.7	3.0	0.27	4.5	4.8	0.01
2018	9.6	3.2	0.26	5.0	4.9	<0.01
2019	7.2	20.5	0.40	1.4	1.7	0.02
Medical history[b]						
Gastroesophageal reflux disease	9.2	10.8	0.05	4.9	6.0	0.05
Peptic ulcer	4.2	6.0	0.08	7.2	6.8	0.01
Gastritis	10.4	8.9	0.05	6.6	7.5	0.03
Crohn's disease	0.2	0.3	0.02	0.3	0.3	0.01
Chronic liver disease	1.7	1.8	0.01	1.6	1.9	0.02
Hypertensive disorder	34.2	41.2	0.14	46.1	44.9	0.02
Osteoarthritis	21.2	24.4	0.08	22.8	23.6	0.02
Diabetes mellitus	14.4	18.0	0.10	21.6	20.1	0.04

© 2023 You SC et al. *JAMA Network Open.*

Depressive disorder	12.1	12.6	0.02	11.0	11.2	<0.01
Renal impairment	5.3	6.6	0.06	5.9	5.9	<0.01
Rheumatoid arthritis	0.3	0.4	0.02	0.3	0.3	0.01
Dementia	2.1	2.1	<0.01	2.4	2.4	<0.01
Charlson comorbidity index, score	0.8	1.0	0.14	1.1	1.1	0.04
Medication use[c]						
Antiinflammatory and antirheumatic products	48.0	44.6	0.07	43.9	43.7	<0.01
Lipid modifying agents	34.6	38.5	0.08	47.2	44.1	0.06
Antidepressants	24.3	25.4	0.02	23.7	24.2	0.01
Antithrombotic agents	29.1	39.4	0.22	50.9	46.9	0.08
Immunosuppressants	1.7	1.8	0.01	1.6	1.4	0.01

Values are presented as proportion of the patients (%) unless otherwise indicated.

To account for baseline differences between the two groups, PS-based matching was used. PSs were calculated in each database independently, based on available demographic characteristics, as well as the medical, medication, procedure exposure history, and baseline laboratory values of each database. Here, we reported the aggregated balance before and after matching only for limited covariates from three databases. The whole balance data before and after PS adjustment for more than 9,000 baseline covariates in each database are available at https://github.com/OHDSI/ShinyDeploy/tree/master/RanitidineCancerRisk/data.

[a]Age groups over 85 were omitted.

[b]Medical history was identified by coded medical diagnosis within 1 year prior to the cohort entry.
[c]Medication use was identified by medication records within 1 year prior to the cohort entry. Both ATC class-level and ingredient-level drug uses were used to fit the PS model. The only class-level balances of drugs before and after PS matching is reported in this table.

Abbreviation: Std.diff, standardized difference; PS, propensity score; SIDIAP, The Information System for Research In Primary Care

© 2023 You SC et al. *JAMA Network Open.*

eTable 7. Baseline characteristics of patients in the NHIS-NSC

Characteristic	Before propensity score matching			After propensity score matching		
	Ranitidine	Other H_2RAs	Std. diff	Ranitidine	Other H_2RAs	Std. diff
Age in year	53.9	53.5	0.02	53.3	53.1	0.01
Age group[a]						
20 - 24	2.9	3.2	0.02	2.8	3.2	0.02
25 - 29	3.9	3.8	<0.01	3.8	3.7	0.01
30 - 34	5.3	5.6	0.01	5.5	5.6	<0.01
35 - 39	7.0	7.2	<0.01	7.7	7.4	0.01
40 - 44	9.1	9.5	0.01	9.3	9.8	0.02
45 - 49	11.4	11.7	0.01	12.1	12.2	<0.01
50 - 54	12.7	11.9	0.02	12.5	12.2	0.01
55 - 59	11.1	10.6	0.01	10.5	10.6	<0.01
60 - 64	10.2	10.0	<0.01	10.5	10.1	0.01
65 - 69	8.8	9.5	0.03	9.3	9.4	<0.01
70 - 74	7.5	7.4	<0.01	7.5	7.0	0.02
75 - 79	5.2	5.2	<0.01	4.4	4.8	0.02
Gender						
Women	54.4	55.7	0.03	54.8	55.2	0.01
Men	45.6	44.3	0.03	45.2	44.8	0.01
Inclusion year						
2003	8.1	12.7	0.15	13.8	12.6	0.04

© 2023 You SC et al. *JAMA Network Open.*

2004	6.4	10.6	0.15	11.0	10.1	0.03
2005	6.5	10.2	0.14	10.4	10.0	0.01
2006	6.4	9.6	0.12	10.3	9.9	0.02
2007	7.3	8.9	0.06	9.0	9.5	0.01
2008	8.7	9.2	0.02	9.5	10.0	0.02
2009	10.4	8.0	0.08	8.4	9.0	0.02
2010	10.1	7.7	0.08	8.4	8.8	0.01
2011	14.2	10.2	0.12	11.4	11.7	0.01
2012	11.7	7.4	0.15	7.7	8.4	0.02
Medical history[b]						
Gastroesophageal reflux disease	14.0	11.5	0.08	11.9	11.5	0.01
Peptic ulcer	14.9	14.7	<0.01	15.7	15.1	0.01
Gastritis	75.3	68.8	0.14	67.9	68.9	0.02
Crohn's disease	0.1	0.2	0.01	0.2	0.2	0.01
Chronic liver disease	6.3	6.4	<0.01	6.5	6.5	<0.01
Hypertensive disorder	32.6	30.6	0.04	30.1	30.1	<0.01
Osteoarthritis	13.7	14.0	0.01	13.5	13.2	0.01
Diabetes mellitus	14.6	13.7	0.03	13.3	13.1	<0.01
Depressive disorder	7.3	6.9	0.01	6.9	6.6	0.01
Renal impairment	1.7	1.3	0.04	1.3	1.2	0.02
Rheumatoid arthritis	5.0	4.4	0.03	4.5	4.5	<0.01
Dementia	2.0	1.5	0.04	1.3	1.3	<0.01

© 2023 You SC et al. *JAMA Network Open.*

Charlson comorbidity index, score	2.7	2.4	0.13	2.3	2.4	0.02
Medication use[c]						
Antiinflammatory and antirheumatic products	59.8	59.3	0.01	59.1	58.3	0.02
Lipid modifying agents	14.9	12.9	0.06	12.1	12.8	0.02
Antidepressants	10.4	10.5	<0.01	9.6	9.9	0.01
Antithrombotic agents	51.9	49.3	0.05	48.1	48.0	<0.01
Immunosuppressants	1.3	0.9	0.03	1.0	1.0	<0.01

Values are presented as proportion of the patients (%) unless otherwise indicated.

To account for baseline differences between the two groups, PS-based matching was used. PSs were calculated in each database independently, based on available demographic characteristics, as well as the medical, medication, procedure exposure history, and baseline laboratory values of each database. Here, we reported the aggregated balance before and after matching only for limited covariates from three databases. The whole balance data before and after PS adjustment for more than 9,000 baseline covariates in each database are available at https://github.com/OHDSI/ShinyDeploy/tree/master/RanitidineCancerRisk/data.

[a]Age groups over 80 were omitted.

[b]Medical history was identified by coded medical diagnosis within 1 year prior to the cohort entry.
[c]Medication use was identified by medication records within 1 year prior to the cohort entry. Both ATC class-level and ingredient-level drug uses were used to fit the PS model. The only class-level balances of drugs before and after PS matching is reported in this table.

Abbreviation: Std.diff, standardized difference; PS, propensity score; NHIS-NSC, Korean National Health Insurance System-National Sample Cohort

© 2023 You SC et al. *JAMA Network Open.*

eTable 8. Baseline characteristics of patients in the AUSOM

Characteristic	Before propensity score matching			After propensity score matching		
	Ranitidine	Other H_2RAs	Std. diff	Ranitidine	Other H_2RAs	Std. diff
Age in year	55.3	53.9	0.09	56.2	55.7	0.03
Age group[a]						
20 - 24	2.3	2.6	0.02	1.7	1.8	0.01
25 - 29	2.9	3.8	0.05	2.3	2.7	0.02
30 - 34	4.6	5.3	0.03	4.4	4.6	0.01
35 - 39	6.6	7.7	0.04	5.8	6.6	0.03
40 - 44	8.8	10.6	0.06	8.8	9.0	0.01
45 - 49	10.6	9.9	0.02	9.7	9.6	<0.01
50 - 54	12.6	11.1	0.05	11.2	11.0	<0.01
55 - 59	11.6	11.7	<0.01	12.7	12.8	<0.01
60 - 64	10.5	10.5	<0.01	12.6	11.9	0.02
65 - 69	9.5	8.9	0.02	10.5	9.8	0.02
70 - 74	8.5	7.8	0.03	9.4	9.0	0.01
75 - 79	6.0	5.7	0.01	6.1	6.1	<0.01
80 - 84	3.5	2.9	0.03	3.3	3.6	0.01
Gender						
Women	54.9	53.2	0.03	53.1	54.9	0.04
Men	45.1	46.8	0.03	46.9	45.1	0.04
Inclusion year						

© 2023 You SC et al. *JAMA Network Open.*

1996	0.4	1.0	0.08	1.1	1.4	0.02
1997	0.9	2.1	0.09	2.9	2.7	0.01
1998	2.1	2.4	0.02	4.6	4.0	0.03
1999	2.2	7.3	0.24	7.3	5.5	0.07
2000	2.0	9.0	0.31	7.7	6.4	0.05
2001	2.0	13.8	0.45	8.1	6.5	0.06
2002	3.7	4.3	0.03	4.6	5.9	0.06
2003	1.7	1.9	0.02	1.8	1.9	0.01
2004	1.3	0.7	0.06	0.6	0.9	0.03
2005	2.4	0.6	0.16	1.1	0.8	0.03
2006	2.8	0.7	0.17	0.9	0.8	0.01
2007	2.0	0.7	0.11	0.9	0.8	0.01
2008	3.8	0.7	0.21	0.9	0.7	0.02
2009	6.9	0.9	0.31	1.0	1.3	0.03
2010	7.3	2.3	0.24	2.5	3.4	0.05
2011	6.3	5.7	0.03	6.7	7.3	0.03
2012	9.4	4.7	0.18	6.8	6.2	0.03
2013	8.5	4.4	0.17	5.8	6.5	0.03
2014	9.4	4.2	0.21	6.6	8.1	0.06
2015	8.5	2.7	0.25	4.3	5.0	0.03
2016	4.9	4.9	<0.01	5.9	6.6	0.03
2017	4.0	7.8	0.16	6.2	6.1	<0.01

© 2023 You SC et al. *JAMA Network Open.*

2018	4.7	9.9	0.20	8.0	8.1	<0.01
2019	2.8	7.5	0.21	3.6	3.3	0.02
Race[b]						
Korean	99.1	99.2	0.02	99.1	99.0	<0.01
Medical history[c]						
Gastroesophageal reflux disease	12.5	4.6	0.29	5.1	6.7	0.07
Peptic ulcer	4.9	7.6	0.11	7.8	7.2	0.02
Gastritis	11.6	13.0	0.04	12.8	12.8	<0.01
Crohn's disease	0.1	0.1	0.01	<0.3	<0.3	0.04
Chronic liver disease	1.4	2.3	0.06	1.8	1.9	<0.01
Hypertensive disorder	21.1	20.4	0.02	23.2	22.3	0.02
Osteoarthritis	2.6	0.9	0.13	1.0	1.5	0.04
Diabetes mellitus	9.8	8.2	0.06	9.4	9.7	0.01
Depressive disorder	1.6	2.2	0.05	2.0	2.5	0.04
Rheumatoid arthritis	0.8	3.0	0.16	1.3	1.1	0.02
Dementia	1.1	1.2	<0.01	0.8	1.0	0.02
Charlson comorbidity index, score	0.8	0.9	0.03	0.8	0.8	0.01
Medication use[d]						
Antiinflammatory and antirheumatic products	53.7	43.3	0.21	51	51.8	0.02
Lipid modifying agents	17.4	19.6	0.06	26.3	25.5	0.02
Antidepressants	16.3	11.3	0.14	10.7	12.6	0.06
Antithrombotic agents	31.3	30.6	0.02	37.2	36.0	0.02

© 2023 You SC et al. *JAMA Network Open.*

Immunosuppressants	7.3	14.4	0.23	8.4	8.5	<0.01

Values are presented as proportion of the patients (%) unless otherwise indicated.

To account for baseline differences between the two groups, PS-based matching was used. PSs were calculated in each database independently, based on available demographic characteristics, as well as the medical, medication, procedure exposure history, and baseline laboratory values of each database. Here, we reported the aggregated balance before and after matching only for limited covariates from three databases. The whole balance data before and after PS adjustment for baseline covariates in each database are available at https://github.com/OHDSI/ShinyDeploy/tree/master/RanitidineCancerRisk/data.

[a]Age groups over 85 were omitted.

[b]The race is reported based on the captured information in the database allowing missing values
[c]Medical history was identified by coded medical diagnosis within 1 year prior to the cohort entry.
[d]Medication use was identified by medication records within 1 year prior to the cohort entry. Both ATC class-level and ingredient-level drug uses were used to fit the PS model. The only class-level balances of drugs before and after PS matching is reported in this table.

Abbreviation: Std.diff, standardized difference; PS, propensity score; AUSOM, Ajou University School of Medicine

© 2023 You SC et al. *JAMA Network Open.*

eTable 9. Baseline characteristics of patients in the KDH

Characteristic	Before propensity score matching			After propensity score matching		
	Ranitidine	Other H_2RAs	Std. diff	Ranitidine	Other H_2RAs	Std. diff
Age in year	59.1	55.7	0.22	58.9	58.5	0.03
Age group[a]						
25 - 29	2.3	4.0	0.10	3.1	3.0	0.01
30 - 34	3.1	4.1	0.06	2.8	2.5	0.01
35 - 39	4.3	4.9	0.03	4.2	4.0	0.01
40 - 44	5.8	6.7	0.04	4.4	6.7	0.10
45 - 49	9.6	8.4	0.04	8.7	9.5	0.03
50 - 54	11.5	10.7	0.03	10.9	10.3	0.02
55 - 59	11.8	13.8	0.06	13.6	13.9	0.01
60 - 64	12.3	12.5	0.01	14.0	12.2	0.05
65 - 69	10.3	10.4	<0.01	11.8	11.6	0.01
70 - 74	10.1	7.7	0.08	10.1	9.8	0.01
75 - 79	7.9	6.6	0.05	7.4	7.8	0.02
80 - 84	5.3	3.5	0.09	4.6	5.2	0.03
Gender						
Women	56.4	56.8	0.01	58.9	58.3	0.01
Men	43.6	43.2	0.01	41.1	41.7	0.01
Inclusion year						
2003	7.7	0.7	0.35	3.7	3.5	0.01

© 2023 You SC et al. *JAMA Network Open.*

2004	9.6	0.3	0.44	1.3	1.5	0.02
2005	2.8	0.5	0.18	1.2	1.7	0.04
2006	7.2	1.8	0.26	4.6	6.3	0.07
2007	7.2	1.6	0.27	4.3	3.9	0.02
2008	7.2	1.5	0.28	3.5	3.1	0.03
2009	7.4	1.2	0.31	2.9	3.1	0.01
2010	6.4	3.9	0.12	8.5	9.6	0.04
2011	6.2	4.7	0.07	8.0	9.8	0.06
2012	7.0	3.7	0.14	7.4	7.0	0.01
2013	6.3	9.1	0.11	11.4	8.9	0.08
2014	6.3	16.2	0.32	9.9	14.1	0.13
2015	5.0	14.9	0.34	10.5	8.3	0.08
2016	4.2	13.1	0.32	8.9	7.3	0.06
2017	3.6	9.5	0.24	6.6	6.5	<0.01
2018	3.5	10.3	0.27	6.5	4.8	0.07
Medical history[b]						
Gastroesophageal reflux disease	12.4	10.1	0.07	11.0	12.8	0.05
Peptic ulcer	15.1	4.4	0.37	8.0	10.1	0.07
Gastritis	16.3	17.6	0.04	18.5	18.9	0.01
Chronic liver disease	3.6	0.9	0.18	2.8	2.2	0.04
Hypertensive disorder	11.9	8.6	0.11	12.7	14.4	0.05
Osteoarthritis	1.3	2.1	0.06	2.8	1.7	0.07

© 2023 You SC et al. *JAMA Network Open.*

Diabetes mellitus	4.9	2.5	0.13	3.4	4.1	0.04
Depressive disorder	2.9	1.6	0.09	1.9	2.3	0.03
Renal impairment	3.9	2.1	0.11	3.1	3.6	0.03
Rheumatoid arthritis	1.0	0.2	0.11	1.1	<0.6	0.09
Dementia	1.6	1.3	0.02	1.4	1.1	0.03
Charlson comorbidity index, score	1.0	0.7	0.27	0.9	1.0	0.03
Medication use[c]						
Antiinflammatory and antirheumatic products	48.5	58.9	0.21	57.3	54.8	0.05
Lipid modifying agents	19.9	14.1	0.16	20.7	23.1	0.06
Antidepressants	16.3	21.4	0.13	18.3	17.8	0.01
Antithrombotic agents	28.4	30.8	0.05	30.3	30.8	0.01
Immunosuppressants	3.5	9.8	0.26	6.1	5.2	0.04

Values are presented as proportion of the patients (%) unless otherwise indicated.

To account for baseline differences between the two groups, PS-based matching was used. PSs were calculated in each database independently, based on available demographic characteristics, as well as the medical, medication, procedure exposure history, and baseline laboratory values of each database. Here, we reported the aggregated balance before and after matching only for limited covariates from three databases. The whole balance data before and after PS adjustment for baseline covariates in each database are available at https://github.com/OHDSI/ShinyDeploy/tree/master/RanitidineCancerRisk/data.

[a]Age groups over 85 were omitted.

[b]Medical history was identified by coded medical diagnosis within 1 year prior to the cohort entry.
[c]Medication use was identified by medication records within 1 year prior to the cohort entry. Both ATC class-level and ingredient-level drug uses were used to fit the PS model. The only class-level balances of drugs before and after PS matching is reported in this table.

Abbreviation: Std.diff, standardized difference; PS, propensity score; KDH, Kandong Sacred Heart Hospital

© 2023 You SC et al. *JAMA Network Open.*

eTable 10. Baseline characteristics of patients in the HUMIC

Characteristic	Before propensity score matching			After propensity score matching		
	Ranitidine	Other H_2RAs	Std. diff	Ranitidine	Other H_2RAs	Std. diff
Age in year	55.1	53.7	0.09	55.3	55.1	0.02
Age group[a]						
20 - 24	4.3	3.9	0.02	2.8	4.1	0.07
25 - 29	5.0	4.4	0.03	3.9	3.7	0.01
30 - 34	4.9	5.7	0.04	5.3	4.3	0.04
35 - 39	5.4	6.0	0.03	5.2	5.4	0.01
40 - 44	6.3	7.7	0.06	6.8	7.1	0.01
45 - 49	8.0	9.3	0.05	9.0	8.4	0.02
50 - 54	10.3	12.4	0.06	11.3	12.2	0.03
55 - 59	12.0	12.2	0.01	12.2	11.9	0.01
60 - 64	12.3	12.6	0.01	13.0	13.7	0.02
65 - 69	11.5	9.7	0.06	12.2	10.9	0.04
75 - 79	6.1	4.8	0.06	5.4	5.6	0.01
80 - 84	3.5	2.4	0.06	2.9	3.1	0.01
Gender						
Women	63.4	56.9	0.13	62.2	63.1	0.02
Men	36.6	43.1	0.13	37.8	36.9	0.02
Inclusion year						
2002	8.0	14.7	0.21	11.9	16.6	0.13

© 2023 You SC et al. *JAMA Network Open.*

2003	4.7	4.9	0.01	6.2	5.8	0.02
2004	4.4	2.9	0.08	3.4	3.0	0.02
2005	4.6	4.5	<0.01	5.4	4.7	0.03
2006	3.9	4.5	0.03	4.9	3.6	0.06
2007	4.0	5.0	0.05	5.6	5.1	0.02
2009	7.3	6.7	0.02	6.3	6.1	0.01
2010	7.4	6.2	0.05	7.4	5.6	0.07
2011	8.3	7.7	0.02	7.8	7.6	<0.01
2012	7.5	6.7	0.03	7.2	8.2	0.04
2013	6.1	6.0	<0.01	5.6	6.3	0.03
2014	6.3	5.3	0.05	5.1	6.1	0.04
2015	5.4	4.4	0.04	4.7	4.5	0.01
2016	4.9	6.2	0.06	7.4	6.9	0.02
2017	6.1	4.8	0.06	6.0	5.3	0.03
2018	5.9	4.4	0.07	0.9	0.3	0.07
Race[b]						
Korean	98.9	98.6	0.02	98.6	99.2	0.07
Medical history[c]						
Gastroesophageal reflux disease	6.7	9.7	0.11	10.2	9.5	0.02
Peptic ulcer	3.8	2.7	0.06	2.7	3.2	0.03
Gastritis	5.2	11.4	0.23	10.4	7.9	0.09
Chronic liver disease	2.4	14.9	0.46	7.5	5.4	0.08

© 2023 You SC et al. *JAMA Network Open.*

Hypertensive disorder	14.2	12.6	0.05	13.9	15.1	0.03
Osteoarthritis	3.3	1.7	0.11	2.3	2.2	0.01
Diabetes mellitus	9.5	8.2	0.05	9.1	9.4	0.01
Depressive disorder	2.3	2.0	0.02	3.2	2.5	0.04
Hyperlipidemia	7.1	8.9	0.06	10.0	9.2	0.03
Renal impairment	2.7	3.5	0.04	3.0	3.1	0.01
Rheumatoid arthritis	10.7	6.9	0.13	8.5	10.8	0.08
Dementia	2.3	1.4	0.07	1.7	1.8	<0.01
Charlson comorbidity index, score	1.0	0.9	0.04	1.0	0.9	0.03
Medication use[d]						
Antiinflammatory and antirheumatic products	51.0	29.5	0.45	35.2	39.4	0.09
Lipid modifying agents	16.0	17.6	0.04	19.9	19.4	0.01
Antidepressants	10.0	8.3	0.06	10.9	10.7	0.01
Antithrombotic agents	23.4	18.0	0.13	22.7	23.3	0.01
Immunosuppressants	20.7	16.0	0.12	16.5	19.7	0.08

Values are presented as proportion of the patients (%) unless otherwise indicated.

To account for baseline differences between the two groups, PS-based matching was used. PSs were calculated in each database independently, based on available demographic characteristics, as well as the medical, medication, procedure exposure history, and baseline laboratory values of each database. Here, we reported the aggregated balance before and after matching only for limited covariates from three databases. The whole balance data before and after PS adjustment for baseline covariates in each database are available at https://github.com/OHDSI/ShinyDeploy/tree/master/RanitidineCancerRisk/data.

[a]Age groups over 85 were omitted.
[b]The race is reported based on the captured information in the database allowing missing values
[c]Medical history was identified by coded medical diagnosis within 1 year prior to the cohort entry.
[d]Medication use was identified by medication records within 1 year prior to the cohort entry. Both ATC class-level and ingredient-level drug uses were used to fit the PS model. The only class-level balances of drugs before and after PS matching is reported in this table.
Abbreviation: Std.diff, standardized difference; PS, propensity score; HUMIC, Hanyang University Medical Center

© 2023 You SC et al. *JAMA Network Open.*

eTable 11. Baseline characteristics of patients in the TMUCRD

Characteristic	Before propensity score matching			After propensity score matching		
	Ranitidine	Other H_2RAs	Std. diff	Ranitidine	Other H_2RAs	Std. diff
Age in year	56.1	58.0	0.12	56.4	57.1	0.04
Age group[a]						
20 - 24	2.2	1.3	0.07	2.1	1.5	0.04
25 - 29	2.8	2.5	0.02	3.0	2.9	<0.01
30 - 34	4.7	3.8	0.04	4.3	3.1	0.06
35 - 39	6.6	5.1	0.07	6.5	5.3	0.05
40 - 44	7.9	7.0	0.03	7.8	7.4	0.01
45 - 49	9.5	9.1	0.02	9.2	9.8	0.02
50 - 54	11.6	12.3	0.02	11.7	12.6	0.03
55 - 59	13.5	14.1	0.01	13.5	13.8	0.01
60 - 64	12.3	12.2	<0.01	12.9	13.1	<0.01
65 - 69	8.5	9.2	0.02	7.9	9.0	0.04
70 - 74	6.8	7.2	0.01	7.6	7.8	0.01
75 - 79	4.8	6.0	0.05	4.8	5.3	0.02
80 - 84	4.2	5.4	0.06	4.7	4.8	<0.01
Gender						
Women	55.8	53.1	0.05	56.3	54.5	0.04
Men	44.2	46.9	0.05	43.7	45.5	0.04
Inclusion year						

© 2023 You SC et al. *JAMA Network Open.*

2008	<0.3	0.8	0.11	<0.3	0.9	0.10
2009	0.5	3.2	0.20	1.2	1.1	0.01
2010	2.0	14.6	0.47	4.2	3.4	0.04
2011	4.9	18.2	0.42	10.3	7.8	0.09
2012	17.3	26.5	0.22	30.6	27.9	0.06
2013	24.0	24.5	0.01	33.8	32.9	0.02
2014	30.4	12.1	0.46	19.7	26.0	0.15
Medical history[b]						
Gastroesophageal reflux disease	7.9	7.3	0.02	8.7	8.6	<0.01
Chronic liver disease	13.3	12.9	0.01	13.9	13.3	0.01
Hypertensive disorder	17.3	21.5	0.11	19.5	19.4	<0.01
Osteoarthritis	17.3	18.8	0.04	18.3	18.8	0.01
Diabetes mellitus	10.9	12.7	0.06	12.1	12.2	<0.01
Depressive disorder	2.6	3.3	0.04	2.8	3.1	0.02
Renal impairment	1.3	2.7	0.10	1.2	1.7	0.04
Rheumatoid arthritis	0.6	1.4	0.08	0.7	0.9	0.03
Dementia	1.8	2.4	0.04	1.6	2.3	0.04
Charlson comorbidity index, score	1.4	1.5	0.08	1.4	1.5	0.03
Medication use[c]						
Antiinflammatory and antirheumatic products	40.9	49.5	0.17	45.4	44.3	0.02
Lipid modifying agents	15.8	19.2	0.09	18.1	17.9	0.01
Antidepressants	7.7	9.4	0.06	8.3	7.4	0.04

© 2023 You SC et al. *JAMA Network Open.*

Antithrombotic agents	17.8	24.7	0.17	21.1	18.6	0.06
Immunosuppressants	0.4	1.2	0.10	0.4	1.0	0.07

Values are presented as proportion of the patients (%) unless otherwise indicated.

To account for baseline differences between the two groups, PS-based matching was used. PSs were calculated in each database independently, based on available demographic characteristics, as well as the medical, medication, procedure exposure history, and baseline laboratory values of each database. Here, we reported the aggregated balance before and after matching only for limited covariates from three databases. The whole balance data before and after PS adjustment for baseline covariates in each database are available at https://github.com/OHDSI/ShinyDeploy/tree/master/RanitidineCancerRisk/data.

[a]Age groups over 85 were omitted.

[b]Medical history was identified by coded medical diagnosis within 1 year prior to the cohort entry.
[c]Medication use was identified by medication records within 1 year prior to the cohort entry. Both ATC class-level and ingredient-level drug uses were used to fit the PS model. The only class-level balances of drugs before and after PS matching is reported in this table.

Abbreviation: Std.diff, standardized difference; PS, propensity score; TMUCRD, Taipei Medical University Clinical Research Database

© 2023 You SC et al. *JAMA Network Open.*

eTable 12. Study population before and after PS matching

Source	Before matching, n		After matching, n		Follow-up duration, year[a]	
	Ranitidine	Other H_2RAs	Ranitidine	Other H_2RAs	Ranitidine	Other H_2RAs
AmbEMR	496 591	211 429	190 814	190 814	2.61	2.60
CUIMC	16 717	17 909	10 813	10 813	3.72	3.58
STARR	6 388	9 649	3 294	3 294	3.12	3.08
DA Germany	39 930	3 026	2 974	2 974	8.08	8.16
IMRD	178 298	633	633	633	7.12	7.50
SIDIAP	113 755	3 315	3 177	3 177	5.82	5.83
NHIS-NSC	30 400	13 525	12 602	12 602	4.54	4.46
AUSOM	9 083	4 838	1 937	1 937	6.61	6.33
HUMIC	10 503	2 130	1 456	1 456	5.28	5.46
KDH	3 942	3 628	909	909	4.26	4.32
TMUCDR	3 561	4 749	1 761	1 761	3.40	3.34

[a]Follow-up duration of study population after PS matching

Abbreviation: PS, propensity score; AmbEMR, IQVIA US Ambulatory EMR; CUIMC, Columbia University Irving Medical Center data warehouse; STARR, Stanford University database warehouse; DA Germany, IQVIA Disease Analyzer Germany; IMRD, UK's IQVIA Medical Research Data; SIDIAP, The Information System for Research in Primary Care; AUSOM, Ajou University School of Medicine; HUMIC, Hanyang University Medical Center; KDH, Kandong Sacred Heart Hospital; NHIS-NSC, Korean National Health Insurance System-National Sample Cohort; TMUCDR, Taipei Medical University Clinical Research Database; H_2RA, histamine-2 receptor antagonist

© 2023 You SC et al. *JAMA Network Open.*

eTable 13. Interaction of cumulative dose and comparative risk of primary outcome between ranitidine versus other H_2RAs users

Source	Cumulative dose, unit[a]	Ranitidine, n (%)[b]	Other H_2RAs, n (%[b])	HRR[c]	*P* for interaction
AmbEMR	365	10019 (5.3%)	9889 (5.2%)	0.98 (0.85-1.13)	0.75
	730	2052 (1.1%)	2018 (1.1%)	0.93 (0.69-1.24)	0.61
	1095	768 (0.4%)	651 (0.3%)	0.76 (0.47-1.23)	0.27
CUIMC	365	756 (7.0%)	757 (7.0%)	0.75 (0.50-1.11)	0.15
	730	265 (2.5%)	264 (2.4%)	1.00 (0.54-1.86)	1.00
	1095	119 (1.1%)	126 (1.2%)	1.54 (0.63-3.95)	0.36
NHIS-NSC	365	759 (23.9%)	755 (23.8%)	1.12 (0.82-1.54)	0.48
	730	295 (9.3%)	271 (8.5%)	1.07 (0.65-1.78)	0.80
	1095	158 (5.0%)	144 (4.5%)	1.12 (0.58-2.22)	0.74
SIDIAP	365	2008 (15.9%)	1964 (15.6%)	1.08 (0.79-1.49)	0.63
	730	1540 (12.2%)	1533 (12.2%)	1.05 (0.78-1.41)	0.74
	1095	1260 (10.0%)	1240 (9.8%)	1.17 (0.87-1.56)	0.30

[a]Cumulative dose (in units) was determined by summing the values in the *quantity* column of the DRUG_EXPOSURE table in OMOP-CDM version 5 during the follow-up duration. For H_2RAs in tablet form, a value of 1 in the *quantity* column represents one tablet prescribed or taken. Cumulative dose is sum of the *quantity* values recorded during patients' entire follow-up period.

[b]For each subgroup, we have provided the number of subjects within that subgroup (i.e., those taking H2RAs with a cumulative dose greater than the corresponding drug dosage), along with the proportion compared to the total number of patients.

Abbreviations: AmbEMR, IQVIA US Ambulatory EMR; CUIMC, Columbia University Irving Medical Center data warehouse; NHIS-NSC, Korean National Health Insurance System-National Sample Cohort; SIDIAP, The Information System for Research in Primary Care; H_2RA, histamine-2 receptor antagonist, HRR, hazard ratio ratio

© 2023 You SC et al. *JAMA Network Open.*

eFigure 1. Covariate balance plot before and after propensity score matching

1A. Balance in the data sources which passed the diagnostics for the primary analysis

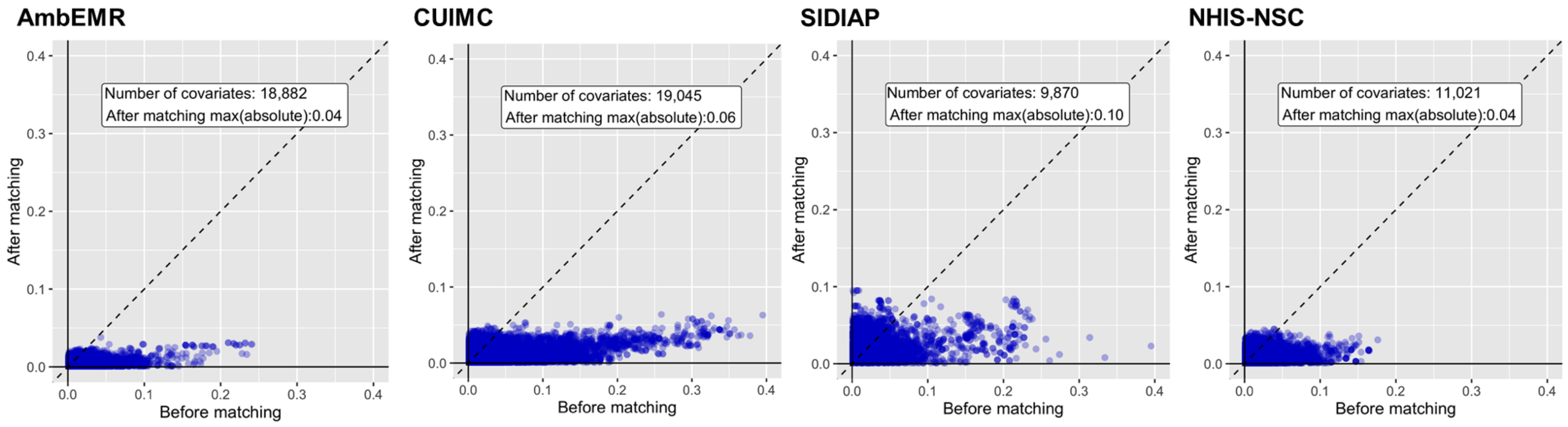

The covariate balances before and after PS matching were depicted. After propensity score matching, every standardized mean difference from more than 9,000 covariates does not exceed 0.1.

Abbreviation: AmbEMR, IQVIA US Ambulatory EMR; CUIMC, Columbia University Irving Medical Center data warehouse; SIDIAP, The Information System for Research In Primary Care; NHIS-NSC, Korean National Health Insurance System-National Sample Cohort

© 2023 You SC et al. *JAMA Network Open.*

1B. Balance in the data sources which did not pass the diagnostics for the primary analysis

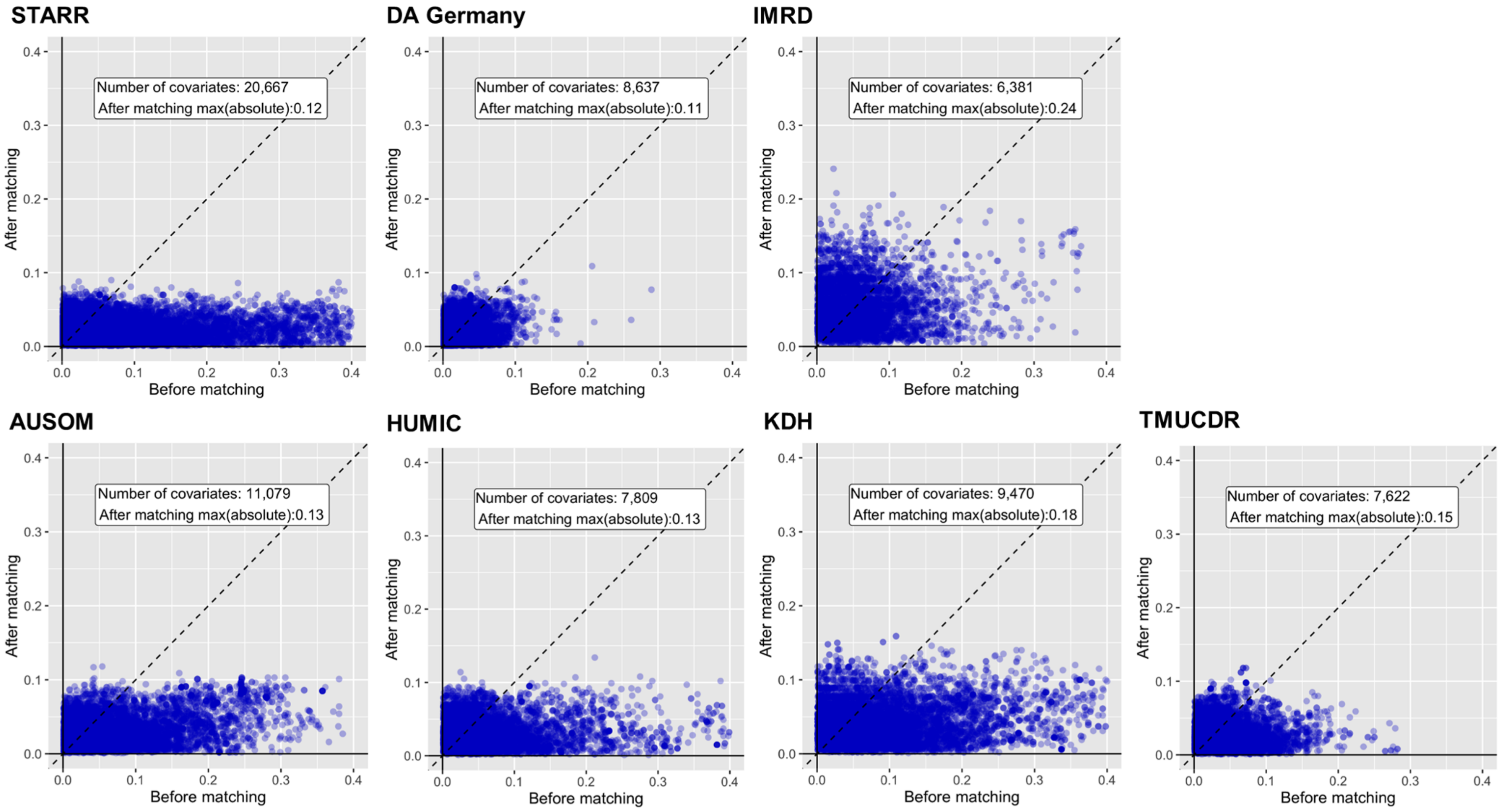

The covariate balances before and after propensity score matching were depicted.

Abbreviations: STARR, Stanford University database warehouse; DA Germany, IQVIA Disease Analyzer Germany; IMRD, UK's IQVIA Medical Research Data; AUSOM, Ajou University School of Medicine; HUMIC, Hanyang University Medical Center; KDH, Kandong Sacred Heart Hospital; TMUCDR, Taipei Medical University Clinical Research Database

© 2023 You SC et al. *JAMA Network Open.*

eFigure 2. Preference score distribution before propensity score matching

2A. Preference score distribution in the data sources which passed the diagnostics for the primary analysis

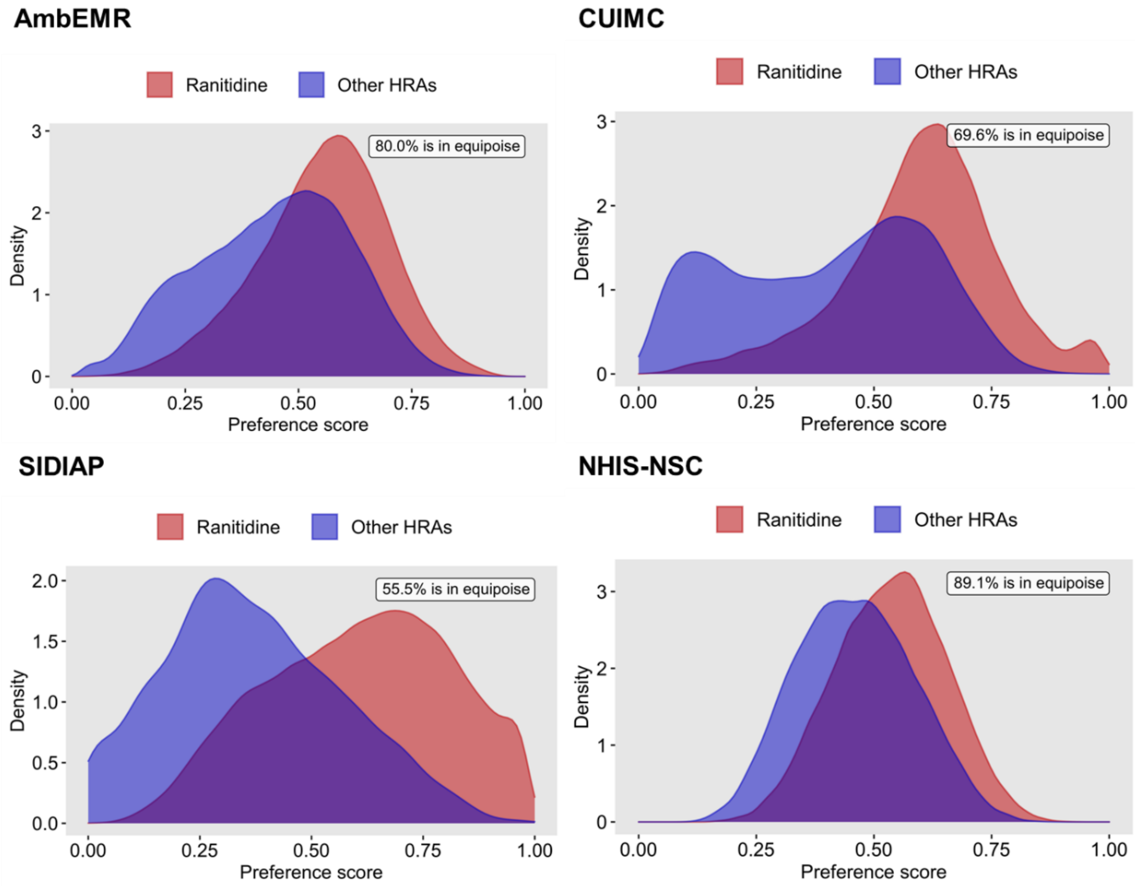

The preference score is a transformation of the propensity score that adjusts for differences in the sizes of the two treatment groups. A higher overlap indicates subjects in the two groups were more similar in terms of their predicted probability of receiving one treatment over the other. This plot shows sufficient equipoise (majority of both

© 2023 You SC et al. *JAMA Network Open.*

distributions being between 0.3 and 0.7) in all four data sources that propensity score matching should be able to create balance without discounting a large proportion of the population, but it shows sufficient difference (non-overlap) that propensity score matching is necessary.
Abbreviation: AmbEMR, IQVIA US Ambulatory EMR; CUIMC, Columbia University Irving Medical Center data warehouse; SIDIAP, The Information System for Research In Primary Care; NHIS-NSC, Korean National Health Insurance System-National Sample Cohort

2B. Preference score distribution in the data sources which did not the diagnostics for the primary analysis

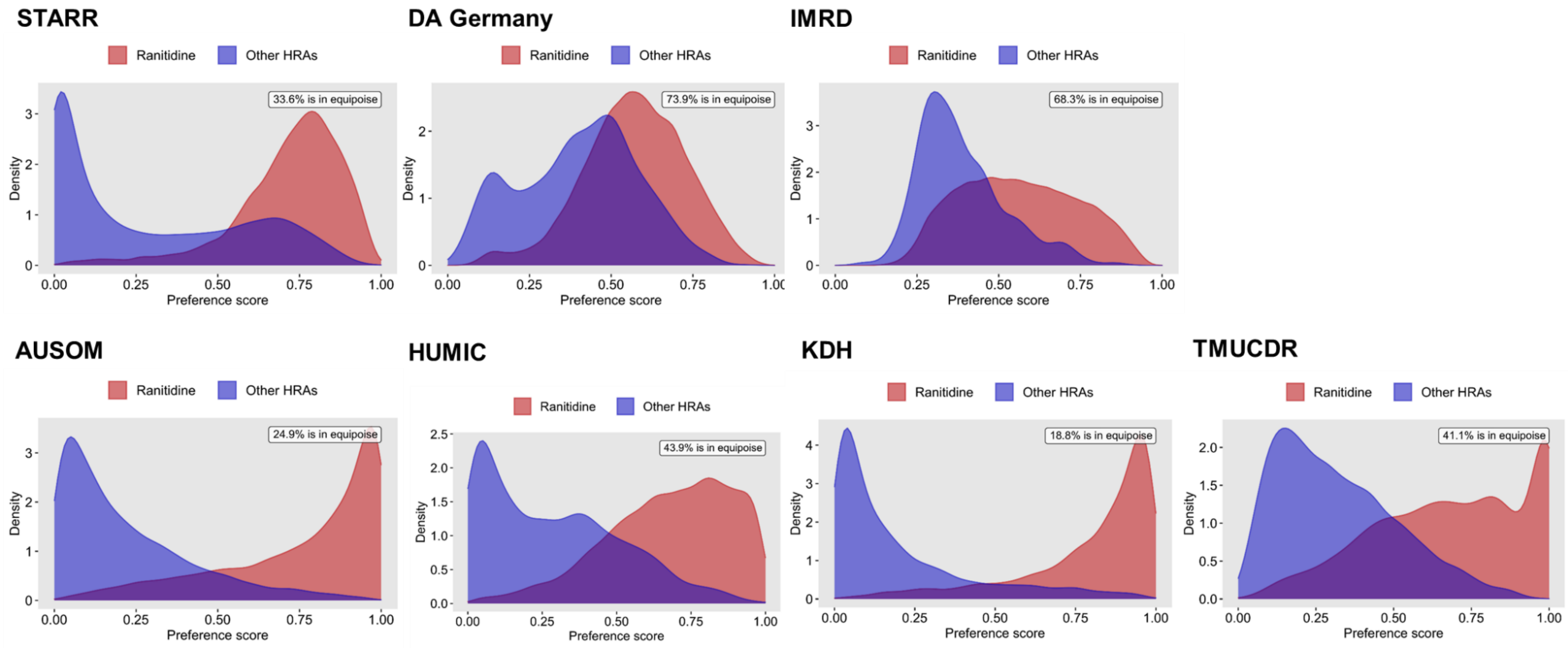

The preference score is a transformation of the propensity score that adjusts for differences in the sizes of the two treatment groups. A higher overlap indicates subjects in the two groups were more similar in terms of their predicted probability of receiving one treatment over the other. Only DA Germany and IMRD show sufficient empirical equipoise (majority of both distributions being between 0.3 and 0.7).

Abbreviations: STARR, Stanford University database warehouse; DA Germany, IQVIA Disease Analyzer Germany; IMRD, UK's IQVIA Medical Research Data; AUSOM, Ajou University School of Medicine; HUMIC, Hanyang University Medical Center; KDH, Kandong Sacred Heart Hospital; TMUCDR, Taipei Medical University Clinical Research Database

© 2023 You SC et al. *JAMA Network Open.*

eFigure 3. Kaplan-Meier plots for primary endpoint between Ranitidine and other H_2 receptor antagonists in data sources which failed to pass diagnostics

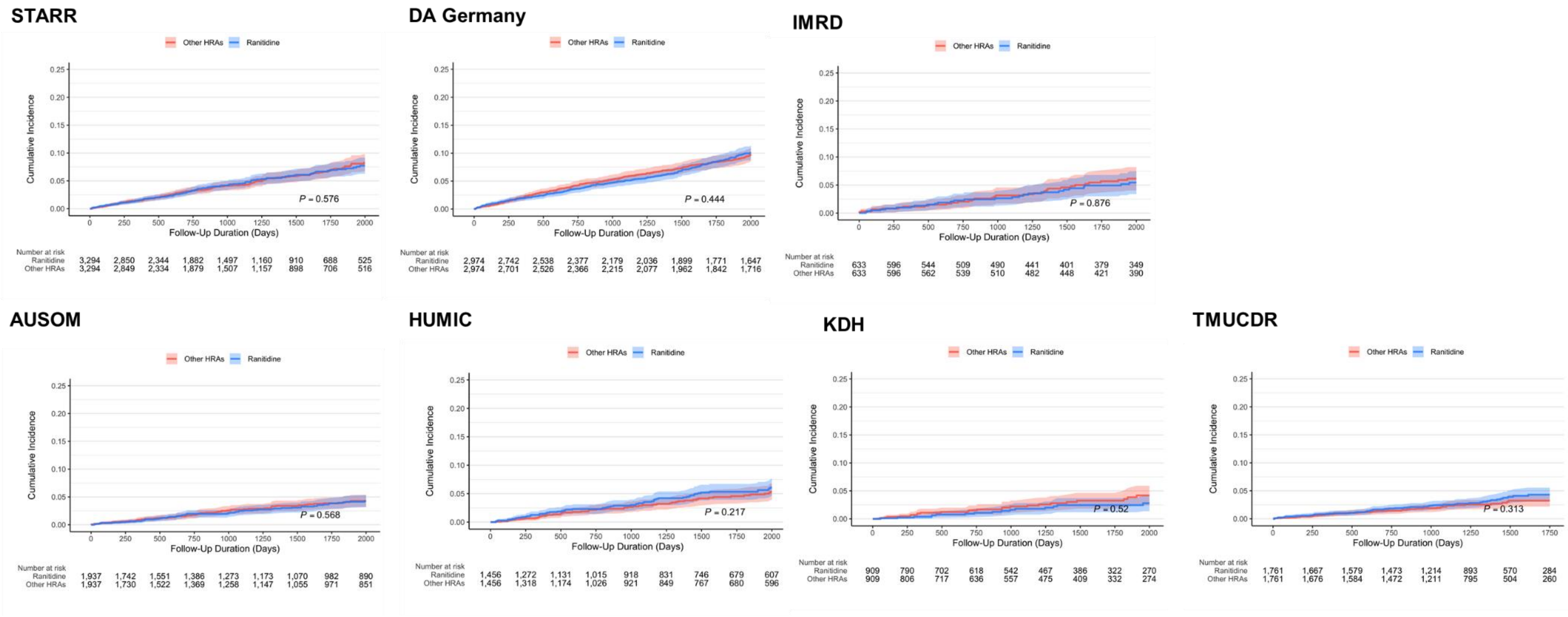

Abbreviations: STARR, Stanford University database warehouse; DA, IQVIA Disease Analyzer; IMRD, UK's IQVIA Medical Research Data; AUSOM, Ajou University School of Medicine; HUMIC, Hanyang University Medical Center; KDH, Kandong Sacred Heart Hospital; TMUCDR, Taipei Medical University Clinical Research Database; HRA, histamine-2 receptor antagonist

© 2023 You SC et al. *JAMA Network Open.*

eFigure 4. Subgroup meta-analysis for primary outcome using results from all available data sources based on follow-up duration

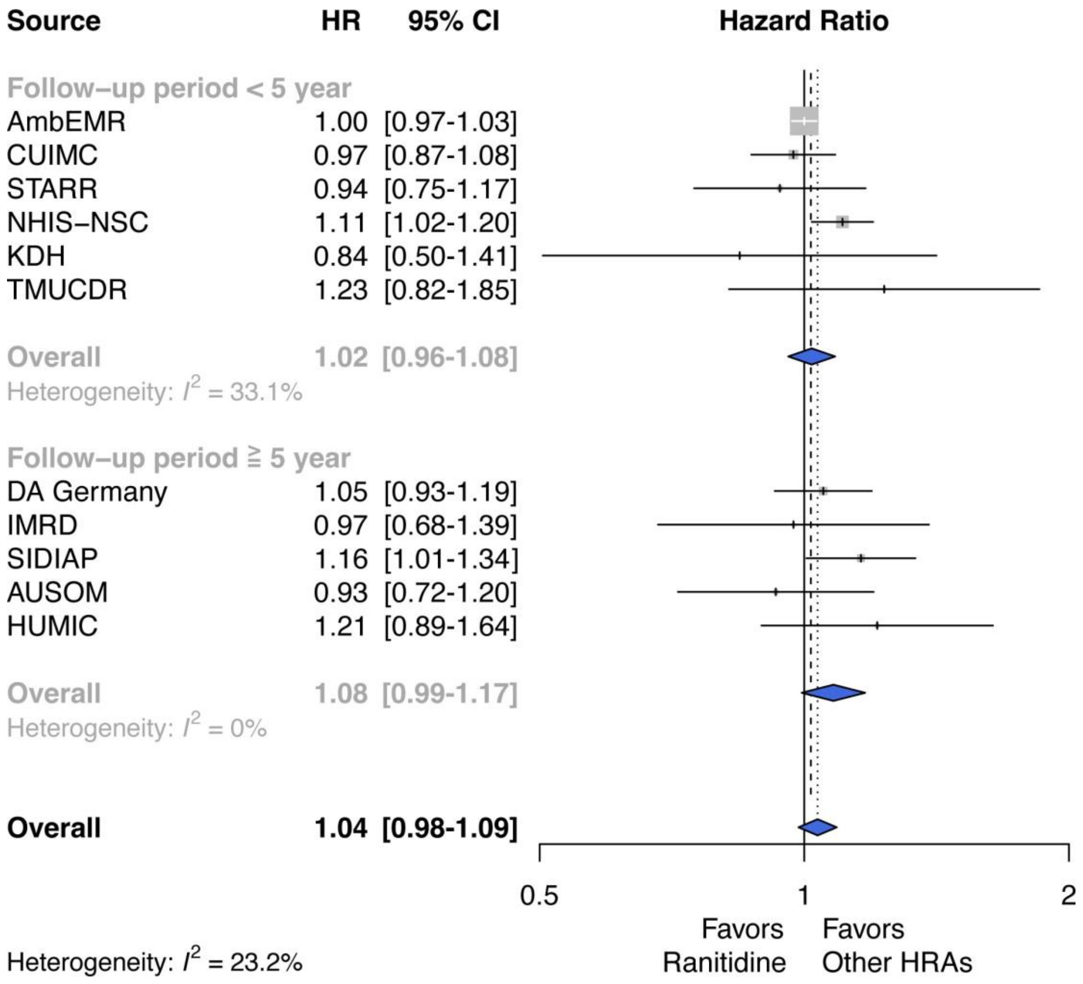

Forest plots depict HR and 95% CI for secondary outcomes. The summary HR were calculated through random-effects model. The hazard ratio greater than 1 means increased risk in the ranitidine group compared with other H_2RAs group. The size of data marker indicates the weight of the study. Error bars indicate 95% CIs.

Abbreviation: AmbEMR, IQVIA US Ambulatory EMR; CUIMC, Columbia University Irving Medical Center data warehouse; SIDIAP, The Information System for Research In Primary Care; NHIS-NSC, Korean National Health Insurance System-National Sample Cohort; HR, hazard ratio; CI, confidence interval; HRA, histamine-2 receptor antagonist

eFigure 5. The risk of the secondary outcomes between ranitidine and other H_2 receptor antagonists

4A. All cancer

Source	Ranitidine Total	Ranitidine Event	Other HRAs Total	Other HRAs Event	HR	95% CI
IQVIA Ambulatory EMR	190,202	8,658	190,202	8,581	1.00	[0.97; 1.03]
CUIMC	10,797	687	10,797	692	0.96	[0.86; 1.07]
SIDIAP	3,175	424	3,175	362	1.17	[1.02; 1.35]
NHIS-NSC	12,602	1,267	12,602	1,125	1.11	[1.02; 1.20]
**Overall**	**216,776**	**11,036**	**216,776**	**10,760**	**1.04**	**[0.97; 1.13]**

Heterogeneity: $I^2 = 70.5\%$

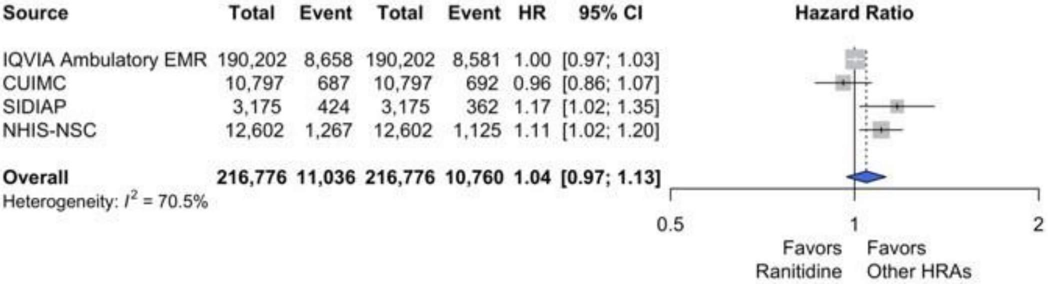

4B. All cancer except thyroid cancer

Source	Ranitidine Total	Ranitidine Event	Other HRAs Total	Other HRAs Event	HR	95% CI
IQVIA Ambulatory EMR	190,304	8,528	190,304	8,445	1.00	[0.97; 1.03]
CUIMC	10,804	680	10,804	678	0.97	[0.87; 1.08]
SIDIAP	3,177	423	3,177	362	1.17	[1.02; 1.35]
NHIS-NSC	12,618	1,198	12,618	1,064	1.10	[1.02; 1.20]
**Overall**	**216,903**	**10,829**	**216,903**	**10,549**	**1.05**	**[0.97; 1.12]**

Heterogeneity: $I^2 = 67.5\%$

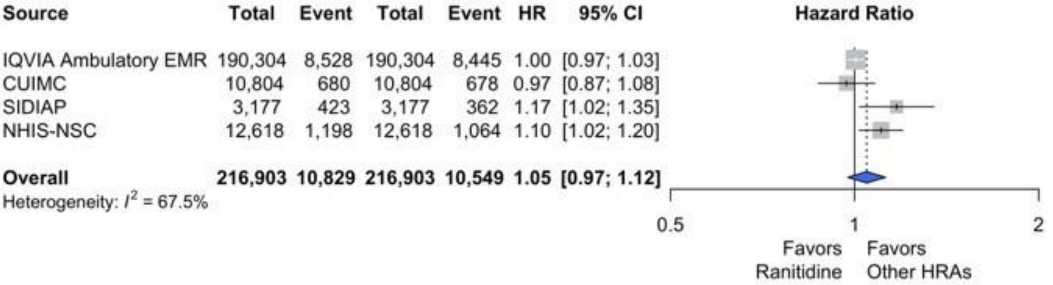

4C. Stomach cancer

Source	Ranitidine Total	Ranitidine Event	Other HRAs Total	Other HRAs Event	HR	95% CI
IQVIA Ambulatory EMR	195,497	57	195,497	58	0.98	[0.68; 1.41]
CUIMC	11,074	8	11,074	8	0.96	[0.35; 2.62]
SIDIAP	3,237	12	3,237	14	0.85	[0.39; 1.85]
NHIS-NSC	12,854	164	12,854	111	1.45	[1.14; 1.84]
**Overall**	**222,662**	**241**	**222,662**	**191**	**1.17**	**[0.88; 1.54]**

Heterogeneity: $I^2 = 32.0\%$

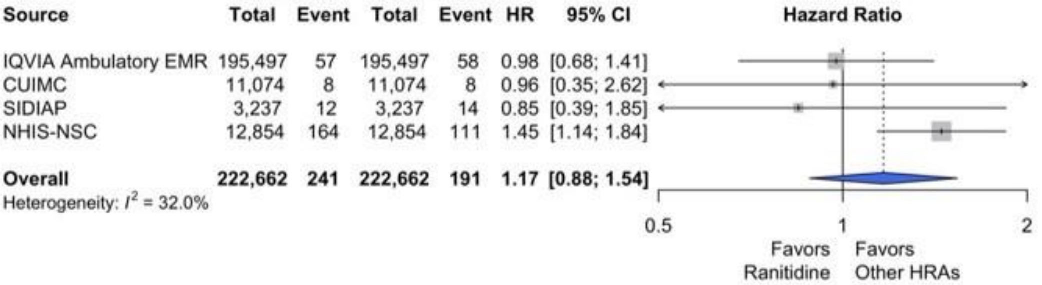

4D. Esophagus cancer

Source	Ranitidine Total	Ranitidine Event	Other HRAs Total	Other HRAs Event	HR	95% CI
IQVIA Ambulatory EMR	195,494	82	195,494	71	1.15	[0.83; 1.58]
CUIMC	11,074	7	11,074	8	0.85	[0.30; 2.39]
SIDIAP	3,238	<5	3,238	6	0.17	[0.02; 1.74]
NHIS-NSC	12,904	13	12,904	12	1.07	[0.48; 2.37]
**Overall**	**222,710**	**97**	**222,710**	**97**	**1.08**	**[0.82; 1.43]**

Heterogeneity: $I^2 = 0.0\%$

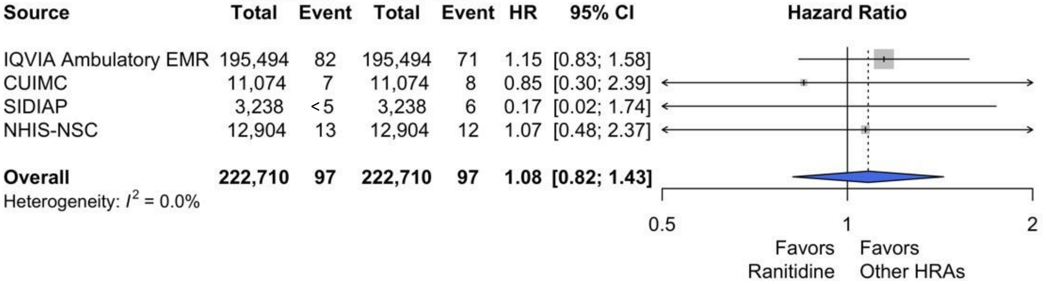

© 2023 You SC et al. *JAMA Network Open.*

4E. Colon and rectum cancer

Source	Ranitidine Total	Ranitidine Event	Other HRAs Total	Other HRAs Event	HR	95% CI
IQVIA Ambulatory EMR	195,257	400	195,257	414	0.96	[0.84; 1.10]
CUIMC	11,060	49	11,060	46	1.03	[0.69; 1.54]
SIDIAP	3,235	46	3,235	42	1.09	[0.71; 1.65]
NHIS-NSC	12,856	234	12,856	219	1.04	[0.87; 1.25]
**Overall**	**222,408**	**729**	**222,408**	**721**	**1.00**	**[0.90; 1.11]**

Heterogeneity: $I^2 = 0.0\%$

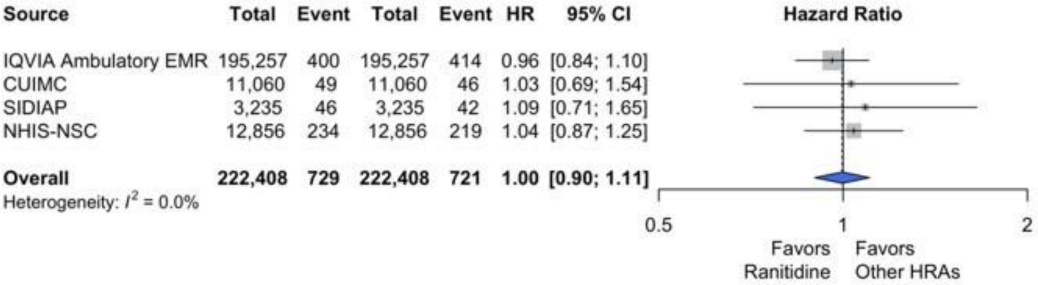

4F. Lung cancer

Source	Ranitidine Total	Ranitidine Event	Other HRAs Total	Other HRAs Event	HR	95% CI
IQVIA Ambulatory EMR	195,128	785	195,128	725	1.08	[0.97; 1.19]
CUIMC	11,057	59	11,057	66	0.86	[0.61; 1.23]
SIDIAP	3,233	42	3,233	38	1.10	[0.71; 1.70]
NHIS-NSC	12,899	60	12,899	50	1.17	[0.80; 1.70]
**Overall**	**222,317**	**946**	**222,317**	**879**	**1.07**	**[0.97; 1.17]**

Heterogeneity: $I^2 = 0.0\%$

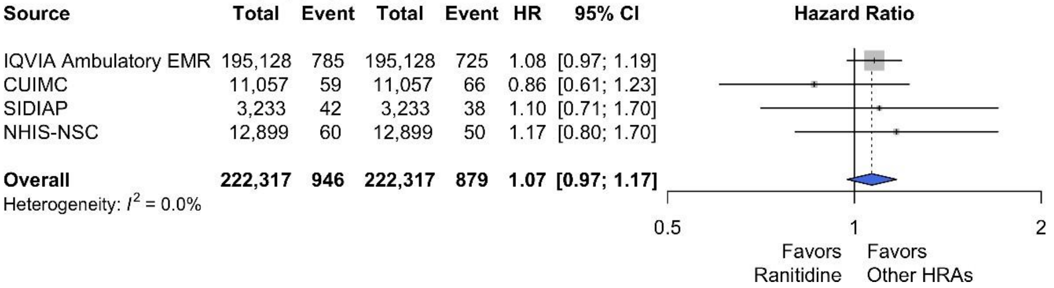

4G. Liver cancer

Source	Ranitidine Total	Ranitidine Event	Other HRAs Total	Other HRAs Event	HR	95% CI
IQVIA Ambulatory EMR	195,488	93	195,488	96	0.96	[0.72; 1.28]
CUIMC	11,070	32	11,070	30	1.02	[0.62; 1.69]
SIDIAP	3,238	8	3,238	7	1.13	[0.40; 3.18]
NHIS-NSC	12,851	253	12,851	246	1.00	[0.84; 1.19]
**Overall**	**222,647**	**386**	**222,647**	**379**	**1.00**	**[0.86; 1.15]**

Heterogeneity: $I^2 = 0.0\%$

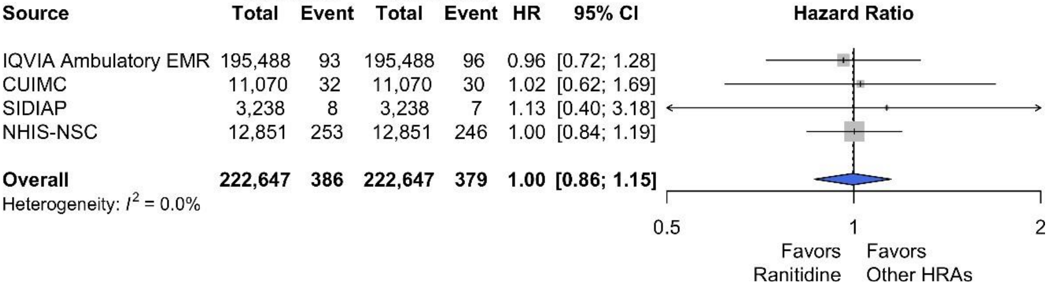

4H. Prostate cancer

Source	Ranitidine Total	Ranitidine Event	Other HRAs Total	Other HRAs Event	HR	95% CI
IQVIA Ambulatory EMR	195,013	830	195,013	869	0.95	[0.86; 1.05]
CUIMC	11,053	71	11,053	70	0.99	[0.71; 1.37]
SIDIAP	3,232	52	3,232	37	1.39	[0.91; 2.12]
NHIS-NSC	12,869	238	12,869	229	1.01	[0.85; 1.22]
**Overall**	**222,167**	**1,191**	**222,167**	**1,205**	**0.98**	**[0.90; 1.07]**

Heterogeneity: $I^2 = 5.2\%$

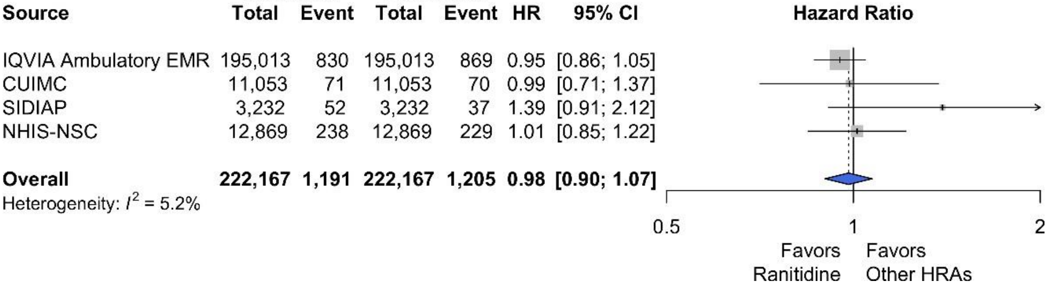

4I. Breast cancer

Source	Ranitidine Total	Ranitidine Event	Other HRAs Total	Other HRAs Event	HR	95% CI
IQVIA Ambulatory EMR	194,947	1,065	194,947	1,114	0.95	[0.87; 1.03]
CUIMC	11,044	81	11,044	104	0.76	[0.57; 1.01]
SIDIAP	3,230	24	3,230	26	0.91	[0.52; 1.59]
NHIS-NSC	12,903	35	12,903	55	0.62	[0.41; 0.95]
**Overall**	**222,124**	**1,205**	**222,124**	**1,299**	**0.84**	**[0.69; 1.02]**

Heterogeneity: $I^2 = 45.7\%$

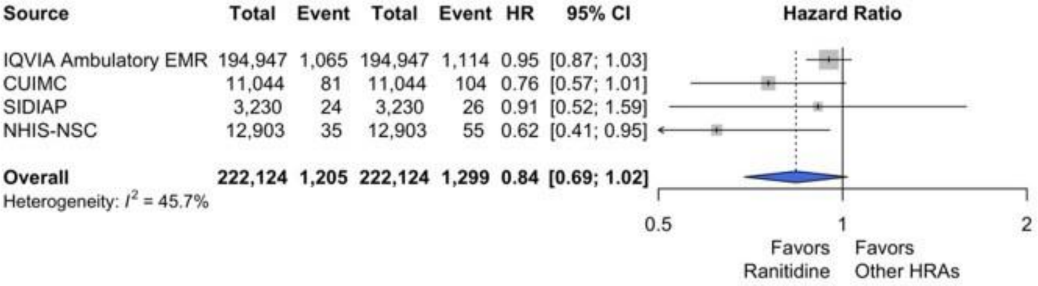

4J. Bladder cancer

© 2023 You SC et al. *JAMA Network Open.*

Source	Ranitidine Total	Ranitidine Event	Other HRAs Total	Other HRAs Event	HR	95% CI
IQVIA Ambulatory EMR	195,382	296	195,382	267	1.10	[0.93; 1.30]
CUIMC	11,072	21	11,072	33	0.62	[0.36; 1.07]
SIDIAP	3,229	36	3,229	40	0.89	[0.57; 1.40]
NHIS-NSC	12,899	32	12,899	41	0.76	[0.48; 1.21]
**Overall**	**222,582**	**385**	**222,582**	**381**	**0.89**	**[0.68; 1.16]**

Heterogeneity: I^2 = 49.2%

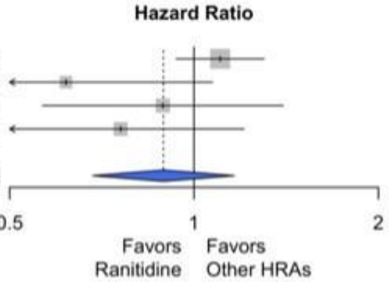

4K. Cervix uteri cancer

Source	Ranitidine Total	Ranitidine Event	Other HRAs Total	Other HRAs Event	HR	95% CI
IQVIA Ambulatory EMR	195,509	45	195,509	47	0.95	[0.63; 1.44]
CUIMC	11,078	5	11,078	10	0.49	[0.16; 1.47]
SIDIAP	3,238	<5	3,238	<5	0.99	[0.04; 25.06]
NHIS-NSC	12,905	20	12,905	22	0.89	[0.48; 1.64]
**Overall**	**222,730**	**65**	**222,730**	**74**	**0.88**	**[0.64; 1.22]**

Heterogeneity: I^2 = 0.0%

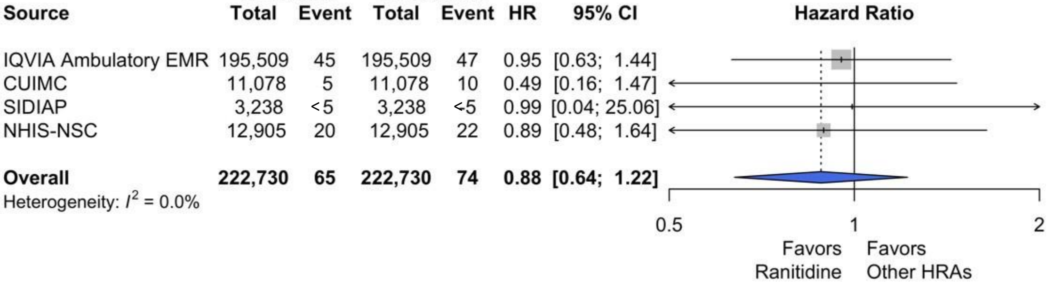

4L. Corpus uteri cancer

Source	Ranitidine Total	Ranitidine Event	Other HRAs Total	Other HRAs Event	HR	95% CI
IQVIA Ambulatory EMR	195,469	190	195,469	146	1.29	[1.04; 1.61]
CUIMC	11,073	23	11,073	21	1.07	[0.59; 1.94]
SIDIAP	3,238	<5	3,238	9	0.44	[0.13; 1.48]
NHIS-NSC	12,907	8	12,907	7	1.12	[0.40; 3.15]
**Overall**	**222,687**	**216**	**222,687**	**183**	**1.20**	**[0.95; 1.52]**

Heterogeneity: I^2 = 6.6%

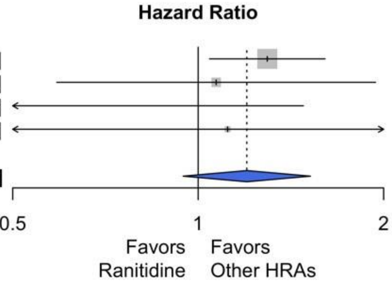

4M. Gall bladder and biliary tract cancer

Source	Ranitidine Total	Ranitidine Event	Other HRAs Total	Other HRAs Event	HR	95% CI
IQVIA Ambulatory EMR	195,531	41	195,531	37	1.10	[0.71; 1.72]
CUIMC	11,076	11	11,076	7	1.50	[0.57; 3.93]
SIDIAP	3,238	<5	3,238	5	0.40	[0.07; 2.25]
NHIS-NSC	12,901	49	12,901	40	1.20	[0.79; 1.82]
**Overall**	**222,746**	**96**	**222,746**	**89**	**1.14**	**[0.86; 1.53]**

Heterogeneity: I^2 = 0.0%

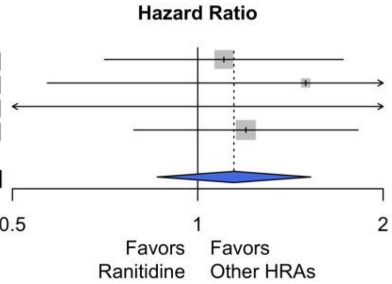

© 2023 You SC et al. *JAMA Network Open.*

4N. Lip, oral cavity and pharynx cancer

Source	Ranitidine Total	Ranitidine Event	Other HRAs Total	Other HRAs Event	HR	95% CI
IQVIA Ambulatory EMR	195,484	106	195,484	123	0.86	[0.66; 1.11]
CUIMC	11,073	23	11,073	24	0.92	[0.51; 1.63]
SIDIAP	3,236	15	3,236	7	2.12	[0.85; 5.29]
NHIS-NSC	12,902	34	12,902	29	1.15	[0.70; 1.89]
**Overall**	**222,695**	**178**	**222,695**	**183**	**1.00**	**[0.76; 1.33]**

Heterogeneity: I^2 = 28.1%

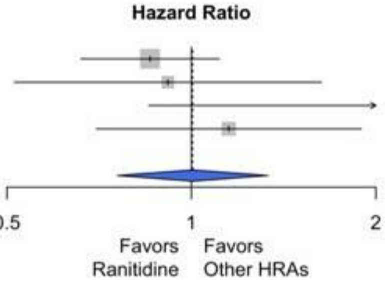

4O. Leukemia

Source	Ranitidine Total	Ranitidine Event	Other HRAs Total	Other HRAs Event	HR	95% CI
IQVIA Ambulatory EMR	195,354	269	195,354	295	0.91	[0.77; 1.07]
CUIMC	11,065	47	11,065	40	1.14	[0.75; 1.74]
SIDIAP	3,237	18	3,237	6	2.98	[1.16; 7.64]
NHIS-NSC	12,904	7	12,904	8	0.86	[0.30; 2.41]
**Overall**	**222,560**	**341**	**222,560**	**349**	**1.12**	**[0.77; 1.63]**

Heterogeneity: I^2 = 55.1%

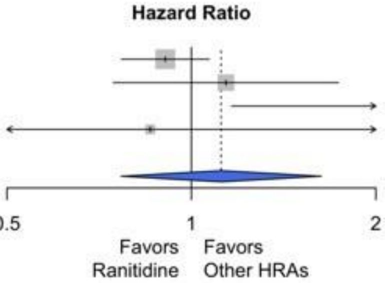

4P. Ovary cancer

Source	Ranitidine Total	Ranitidine Event	Other HRAs Total	Other HRAs Event	HR	95% CI
IQVIA Ambulatory EMR	195,469	103	195,469	84	1.22	[0.91; 1.62]
CUIMC	11,071	14	11,071	12	1.13	[0.52; 2.47]
SIDIAP	3,238	<5	3,238	<5	1.45	[0.21; 9.84]
NHIS-NSC	12,902	50	12,902	35	1.39	[0.90; 2.14]
**Overall**	**222,680**	**162**	**222,680**	**126**	**1.26**	**[1.00; 1.58]**

Heterogeneity: I^2 = 0.0%

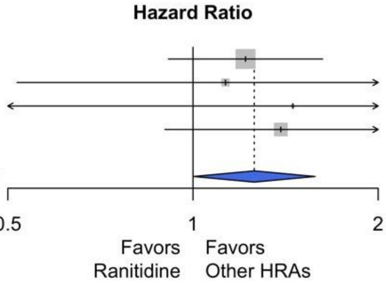

4Q. Pancreas cancer

Source	Ranitidine Total	Ranitidine Event	Other HRAs Total	Other HRAs Event	HR	95% CI
IQVIA Ambulatory EMR	195,468	154	195,468	131	1.17	[0.93; 1.48]
CUIMC	11,068	27	11,068	30	0.87	[0.52; 1.47]
SIDIAP	3,238	13	3,238	14	0.92	[0.43; 1.97]
NHIS-NSC	12,884	133	12,884	154	0.84	[0.66; 1.05]
**Overall**	**222,658**	**327**	**222,658**	**329**	**0.97**	**[0.79; 1.18]**

Heterogeneity: I^2 = 28.9%

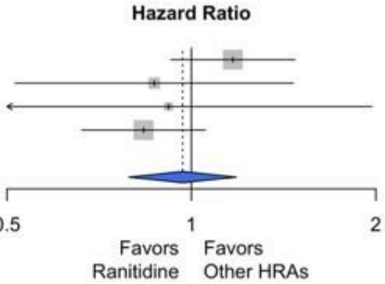

4R. Thyroid cancer

Source	Ranitidine Total	Ranitidine Event	Other HRAs Total	Other HRAs Event	HR	95% CI
IQVIA Ambulatory EMR	195,442	169	195,442	166	1.01	[0.82; 1.25]
CUIMC	11,070	15	11,070	23	0.63	[0.33; 1.22]
SIDIAP	3,236	<5	3,236	<5	1.98	[0.13; 29.61]
NHIS-NSC	12,888	100	12,888	90	1.08	[0.82; 1.44]
**Overall**	**222,636**	**279**	**222,636**	**274**	**1.01**	**[0.85; 1.19]**

Heterogeneity: I^2 = 0.0%

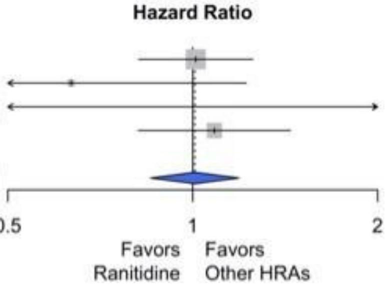

© 2023 You SC et al. *JAMA Network Open.*

Forest plots depict HR and 95% CI for secondary outcomes. The summary HR were calculated through random-effects model. The hazard ratio greater than 1 means increased risk in the ranitidine group compared with other H_2RAs group. The size of data marker indicates the weight of the study. Error bars indicate 95% CIs.

Abbreviation: AmbEMR, IQVIA US Ambulatory EMR; CUIMC, Columbia University Irving Medical Center data warehouse; SIDIAP, The Information System for Research In Primary Care; NHIS-NSC, Korean National Health Insurance System-National Sample Cohort; HR, hazard ratio; CI, confidence interval; HRA, histamine-2 receptor antagonist

© 2023 You SC et al. *JAMA Network Open.*

eFigure 6. Systematic error control of effect estimation in the meta-analysis

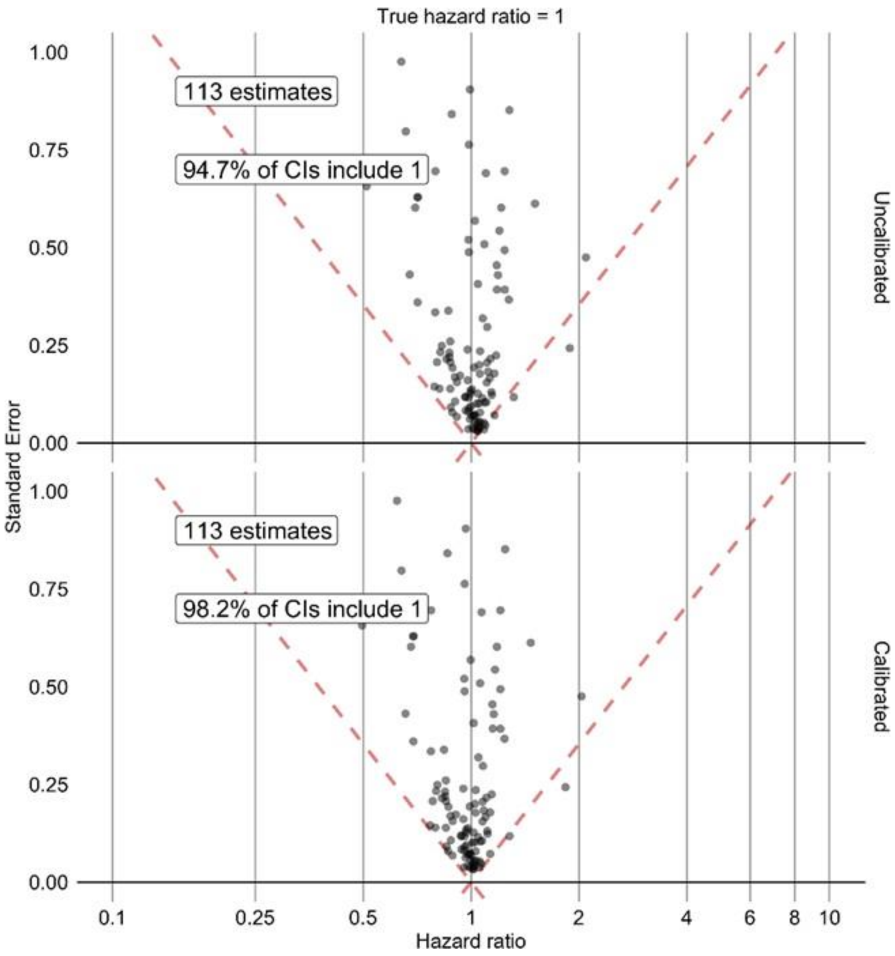

The funnel plots describe the hazard ratio and standard error of each summary estimate of negative control outcomes in the meta-analysis. Top plot is the result before calibration and bottom plot is the result after calibration of confidence interval. Nominal 95% confidence intervals cover 94.7% (107/113) and 98.2% (111/113) before and after calibration of confidence interval, respectively

© 2023 You SC et al. *JAMA Network Open.*

eFigure 7. Sensitivity analyses for risk of primary endpoint between rantidine and other H_2 receptor antagonists using a meta-anlysis and various time-at-risk, statistics, and empiricial calibration

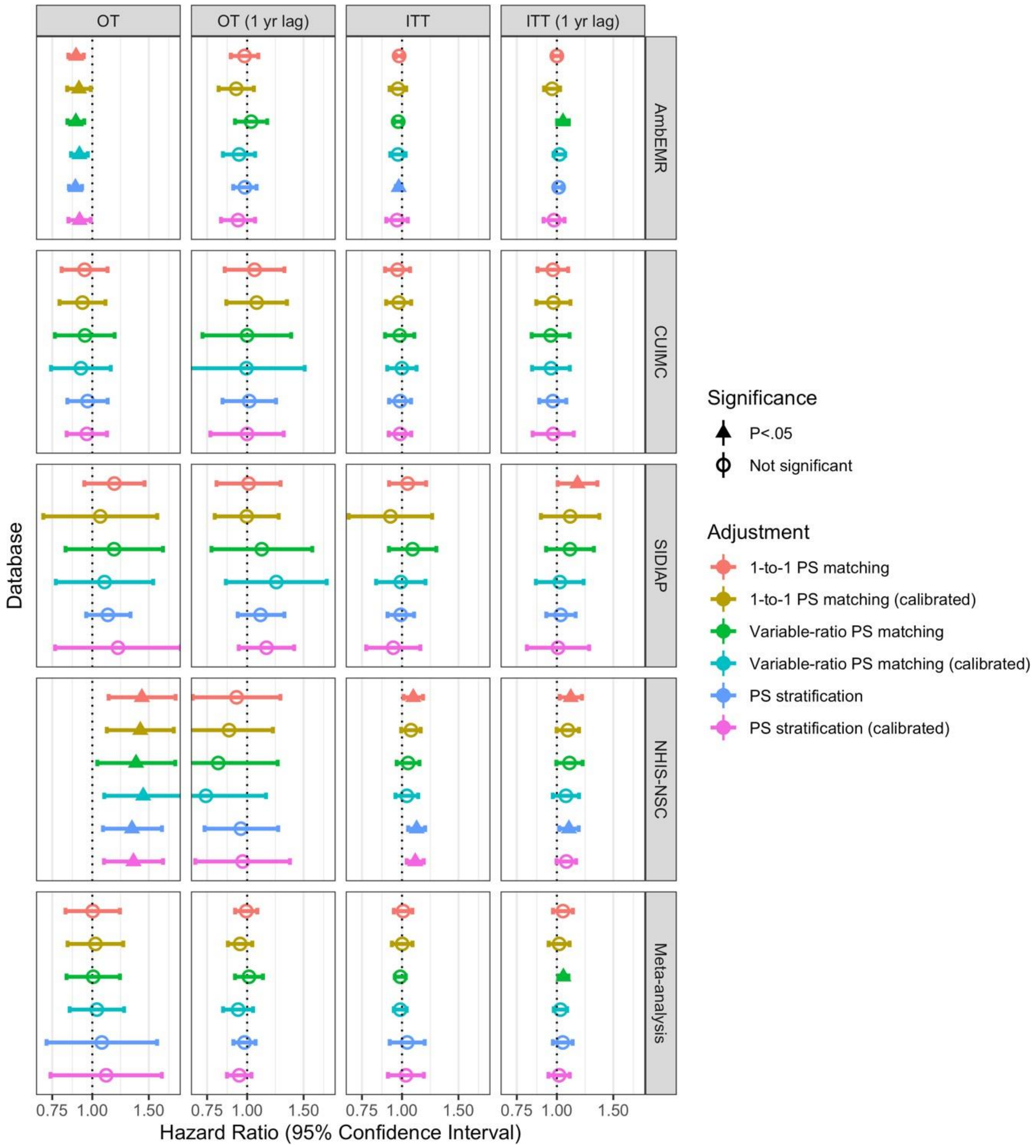

The points indicate HR estimates and the lines their 95% CIs, based on 24 analyses with four different times-at-risk, six different statistical models.. An HR >1 implies a higher risk in the ranitidine group. Open circles represent HRs with CIs that included 1, and triangles represent HRs that were statistically significant. Abbreviations: OT, on treatment analysis; ITT, intention-to-treat analysis; PS, propensity score; HR, hazard ratio; CI, confidence interval; AmbEMR, IQVIA US Ambulatory EMR; CUIMC, Columbia University Irving Medical Center data warehouse; SIDIAP, The Information System for Research In Primary Care; NHIS-NSC, Korean National Health Insurance System-National Sample Cohort

© 2023 You SC et al. *JAMA Network Open.*

eFigure 8. The risk of the primary outcome (all cancer except non-melanoma skin cancer) between ranitidine and cimetidine, famotidine, and nizatidine Users

5A. Ranitidine versus Cimetidine

	Ranitidine		Cimetidine			
**Source**	**Total**	**Event**	**Total**	**Event**	**HR**	**95% CI**
AmbEMR	14,068	905	14,068	907	1.02	[0.93; 1.12]

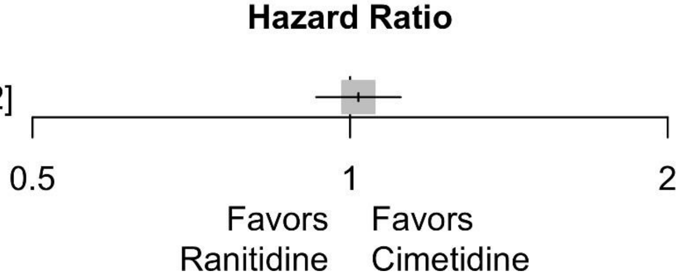

5B. Ranitidine versus Famotidine

	Ranitidine		Famotidine			
**Source**	**Total**	**Event**	**Total**	**Event**	**HR**	**95% CI**
AmbEMR	190,815	7,557	190,815	7,408	1.02	[0.98; 1.05]
CUIMC	10,817	630	10,817	638	0.97	[0.87; 1.08]
DA Germany	2,884	443	2,884	455	0.94	[0.83; 1.08]
SIDIAP	3,162	375	3,162	350	1.07	[0.92; 1.24]
NHIS-NSC	10,839	1,118	10,839	1,015	1.09	[1.00; 1.18]
**Overall**	**218,517**	**10,123**	**218,517**	**9,866**	**1.02**	**[0.98; 1.06]**

Heterogeneity: I^2 = 13.7%

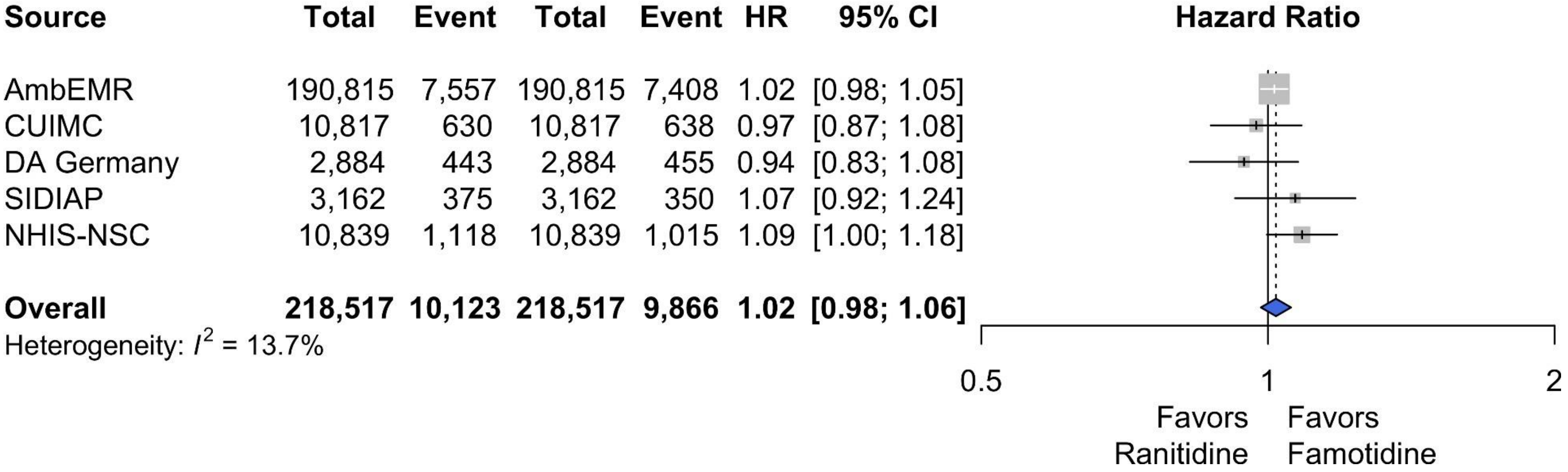

© 2023 You SC et al. *JAMA Network Open.*

5C. Ranitidine versus Nizatidine

Source	Ranitidine Total	Ranitidine Event	Nizatidine Total	Nizatidine Event	HR	95% CI
NHIS-NSC	7,683	700	7,683	648	1.05	[0.94; 1.17]

Hazard Ratio

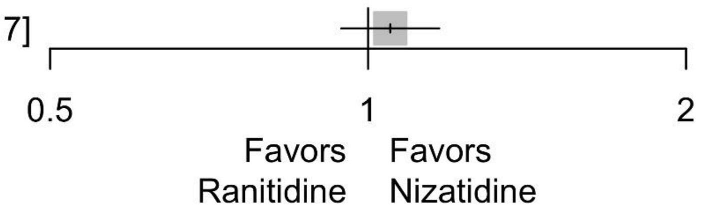

Forest plots depict HR and 95% CI for primary outcomes between ranitidine versus cimetidine (A), famotidine (B), and nizatidine (C). The summary HR were calculated through random-effects model. The hazard ratio greater than 1 means increased risk in the ranitidine group compared with other H_2RAs group. The size of data marker indicates the weight of the study. Error bars indicate 95% CIs.

Abbreviation: AmbEMR, IQVIA US Ambulatory EMR; CUIMC, Columbia University Irving Medical Center data warehouse; SIDIAP, The Information System for Research In Primary Care; NHIS-NSC, Korean National Health Insurance System-National Sample Cohort; DA Germany, IQVIA Disease Analyzer Germany; HR, hazard ratio, CI, confidence interval.

© 2023 You SC et al. *JAMA Network Open.*

eReferences

1 Datta S, Posada J, Olson G, *et al.* A new paradigm for accelerating clinical data science at Stanford Medicine. 2020; published online March 17. https://arxiv.org/abs/2003.10534v1 (accessed July 14, 2020).

2 You SC, Lee S, Cho S-Y, *et al.* Conversion of National Health Insurance Service-National Sample Cohort (NHIS-NSC) Database into Observational Medical Outcomes Partnership-Common Data Model (OMOP-CDM). *Stud Health Technol Inform* 2017; **245**: 467–70.

3 Yoon D, Ahn EK, Park MY, *et al.* Conversion and Data Quality Assessment of Electronic Health Record Data at a Korean Tertiary Teaching Hospital to a Common Data Model for Distributed Network Research. *Healthc Inform Res* 2016; **22**: 54–8.

4 Sung H, Ferlay J, Siegel RL, *et al.* Global Cancer Statistics 2020: GLOBOCAN Estimates of Incidence and Mortality Worldwide for 36 Cancers in 185 Countries. *CA Cancer J Clin* 2021; **71**: 209–49.

5 Tseng K-S, Lin C, Lin Y-S, Weng S-F. Risk of Head and Neck Cancer in Patients With Diabetes Mellitus: A Retrospective Cohort Study in Taiwan. *JAMA Otolaryngol Neck Surg* 2014; **140**: 746–53.

6 Seo HJ, Oh I-H, Yoon S-J. A Comparison of the Cancer Incidence Rates between the National Cancer Registry and Insurance Claims Data in Korea. *Asian Pac J Cancer Prev* 2012; **13**: 6163–8.

7 Kao C-H, Sun L-M, Liang J-A, Chang S-N, Sung F-C, Muo C-H. Relationship of Zolpidem and Cancer Risk: A Taiwanese Population-Based Cohort Study. *Mayo Clin Proc* 2012; **87**: 430–6.

8 Abraha I, Serraino D, Giovannini G, *et al.* Validity of ICD-9-CM codes for breast, lung and colorectal cancers in three Italian administrative healthcare databases: a diagnostic accuracy study protocol. *BMJ Open* 2016; **6**. DOI:10.1136/bmjopen-2015-010547.

9 El-Serag HB, Mason AC. Risk Factors for the Rising Rates of Primary Liver Cancer in the United States. *Arch Intern Med* 2000; **160**: 3227–30.

10 Goldberg DS, Lewis JD, Halpern SD, Weiner MG, Re VL. Validation of a coding algorithm to identify patients with hepatocellular carcinoma in an administrative database. *Pharmacoepidemiol Drug Saf* 2013; **22**: 103–7.

11 Jamal MM, Yoon EJ, Vega KJ, Hashemzadeh M, Chang KJ. Diabetes mellitus as a risk factor for gastrointestinal cancer among American veterans. *World J Gastroenterol WJG* 2009; **15**: 5274–8.

12 Tonelli M, Wiebe N, Fortin M, *et al.* Methods for identifying 30 chronic conditions: application to administrative data. *BMC Med Inform Decis Mak* 2015; **15**: 31.

13 Esposito DB, Banerjee G, Yin R, *et al.* Development and Validation of an Algorithm to Identify Endometrial Adenocarcinoma in US Administrative Claims Data. J. Cancer Epidemiol. 2019. DOI:https://doi.org/10.1155/2019/1938952.

© 2023 You SC et al. *JAMA Network Open.*

14 Lin H-W, Tu Y-Y, Lin SY, *et al.* Risk of ovarian cancer in women with pelvic inflammatory disease: a population-based study. *Lancet Oncol* 2011; **12**: 900–4.

15 Won Y-J, Jung K-W, Oh C-M, *et al.* Geographical Variations and Trends in Major Cancer Incidences throughout Korea during 1999-2013. *Cancer Res Treat* 2018; **50**: 1281–93.

16 Porter MP, Kerrigan MC, Donato BMK, Ramsey SD. Patterns of use of systemic chemotherapy for Medicare beneficiaries with urothelial bladder cancer. *Urol Oncol Semin Orig Investig* 2011; **29**: 252–8.

17 Luo R, Greenberg A, Stone CD. Outcomes of Clostridium difficile Infection in Hospitalized Leukemia Patients: A Nationwide Analysis. *Infect Control Hosp Epidemiol* 2015; **36**: 794–801.

18 Sosa JA, Hanna JW, Robinson KA, Lanman RB. Increases in thyroid nodule fine-needle aspirations, operations, and diagnoses of thyroid cancer in the United States. *Surgery* 2013; **154**: 1420–7.

19 Voss EA, Boyce RD, Ryan PB, van der Lei J, Rijnbeek PR, Schuemie MJ. Accuracy of an automated knowledge base for identifying drug adverse reactions. *J Biomed Inform* 2017; **66**: 72–81.

© 2023 You SC et al. *JAMA Network Open.*
