# Supplementary material for: Ranitidine Use and Incident Cancer in a Multinational Cohort
Source: JAMA Netw Open. 2023 Sep 19;6(9):e2333495. doi: 10.1001/jamanetworkopen.2023.33495 (PMC10509724; doi:10.1001/jamanetworkopen.2023.33495)
Supplement: Supplement 2. — Data Sharing Statement [file jamanetwopen-e2333495-s002.pdf]

Data Sharing Statement

You. Ranitidine Use and Incident Cancer in a Multinational Cohort. *JAMA Netw Open*. Published September 19, 2023. doi:10.1001/jamanetworkopen.2023.33495

Data
Data available: No

Additional Information
Explanation for why data not available: The pre-specified study protocols and open and executable source code are publicly available online (https://github.com/ohdsi-studies/RanitidineCancerRisk). The results without patient-level information are available at https://github.com/OHDSI/ShinyDeploy/tree/master/RanitidineCancerRisk with a dedicated web browser (https://data.ohdsi.org/RanitidineCancerRisk/). The patient-level data were not shared due to concerns regarding patient privacy.
